# Centrally Projecting Edinger-Westphal Nucleus in the Control of Sympathetic Outflow and Energy Homeostasis

**DOI:** 10.3390/brainsci11081005

**Published:** 2021-07-29

**Authors:** Georgina Cano, Shelby L. Hernan, Alan F. Sved

**Affiliations:** Department of Neuroscience, A210 Langley Hall, University of Pittsburgh, Pittsburgh, PA 15260, USA; shh119@pitt.edu (S.L.H.); sved@pitt.edu (A.F.S.)

**Keywords:** Edinger-Westphal nucleus, sympathetic nervous system, energy homeostasis, brown adipose tissue, white adipose tissue

## Abstract

The centrally projecting Edinger-Westphal nucleus (EWcp) is a midbrain neuronal group, adjacent but segregated from the preganglionic Edinger-Westphal nucleus that projects to the ciliary ganglion. The EWcp plays a crucial role in stress responses and in maintaining energy homeostasis under conditions that require an adjustment of energy expenditure, by virtue of modulating heart rate and blood pressure, thermogenesis, food intake, and fat and glucose metabolism. This modulation is ultimately mediated by changes in the sympathetic outflow to several effector organs, including the adrenal gland, heart, kidneys, brown and white adipose tissues and pancreas, in response to environmental conditions and the animal’s energy state, providing for appropriate energy utilization. Classic neuroanatomical studies have shown that the EWcp receives inputs from forebrain regions involved in these functions and projects to presympathetic neuronal populations in the brainstem. Transneuronal tracing with pseudorabies virus has demonstrated that the EWcp is connected polysynaptically with central circuits that provide sympathetic innervation to all these effector organs that are critical for stress responses and energy homeostasis. We propose that EWcp integrates multimodal signals (stress, thermal, metabolic, endocrine, etc.) and modulates the sympathetic output simultaneously to multiple effector organs to maintain energy homeostasis under different conditions that require adjustments of energy demands.

## 1. Introduction

The Edinger-Westphal nucleus (EW) has been classically considered synonymous with the location of the parasympathetic preganglionic neurons that project to the ciliary ganglion and contribute to the oculomotor nerve (cranial nerve III). Although EW is typically considered (but not conclusively established in rodents) as the site of these parasympathetic preganglionic neurons involved with pupillary constriction and lens accommodation, it is also now clear that EW is a much more complex and multifaceted region. More than 40 years ago, Saper et al. [1] noted that injections of horseradish peroxidase (HRP) into the spinal cord and certain brainstem regions retrogradely-labeled neurons in EW in rats, cats and monkeys. Following that observation, Loewy and Saper [2] examined projections from EW in cats using anterograde transport of tritiated amino acids, and they found a variety of brainstem and spinal cord regions receiving input from EW. They concluded that *“the traditional view of the EW nucleus as merely a parasympathetic preganglionic nucleus should be seriously questioned”*. Since then, studies using modern neuroanatomical techniques have consistently documented a large and diverse population of non-parasympathetic preganglionic neurons in EW, with EW neurons innervating multiple regions of the central nervous system (CNS). Much of this anatomical organization, across multiple species, was comprehensively reviewed by Kozicz et al. [3], leading to a proposed nomenclature based on the projection target. Thus, cholinergic parasympathetic preganglionic neurons projecting to the ciliary ganglion were designated as preganglionic EW (EWpg), whereas the rest of EW neurons, which contain several neuropeptides and project to other areas of the CNS, were designated as centrally projecting EW (EWcp). In the current review, we adopt this nomenclature, while acknowledging that EWcp represents diverse populations of neurons that can be defined based on neuroanatomical and neurochemical features [3,4,5].

The boundaries of EW, which sits in the midbrain, vary across species, as does the location of EWpg vs. EWcp (for a detailed description, see the review by Kozicz et al. [3]). Briefly, in non-human primates, birds and reptiles, EWpg is a compact nucleus and the source of cholinergic preganglionic neurons that project to the ciliary ganglion, whereas EWcp appears as a discrete structure, although some neurons are located diffusely in non-human primates [3]. Conversely, in cats, rodents and humans, EWcp conforms a circumscribed cell group located dorsomedial or dorsal (humans) to the oculomotor nucleus, whereas neurons in EWpg are scarce and diffusely distributed [3]; it is unclear whether these scattered neurons are the source of cholinergic projections to the ciliary ganglion. Although EWpg and EWcp are adjacent and intermingled to some extent in several species, these populations differ significantly in their connectivity and neurochemical profile.

Another observation that drew attention to EW as not simply the source of pupillary parasympathetic preganglionic neurons was the discovery of the neuropeptide urocortin-1 (Ucn-1), which belongs to the corticotropin releasing factor (CRF) neuropeptide superfamily [6], and the subsequent finding that EWcp contains the largest population of Ucn-1 neurons in the CNS [6,7]. The presence of Ucn-1 in EWcp is conserved phylogenetically across vertebrates, suggesting an important role in survival [8]. As discussed below, interest in Ucn-1 and its potential involvement in various brain functions has sparked the study of EWcp.

**Figure 1:**
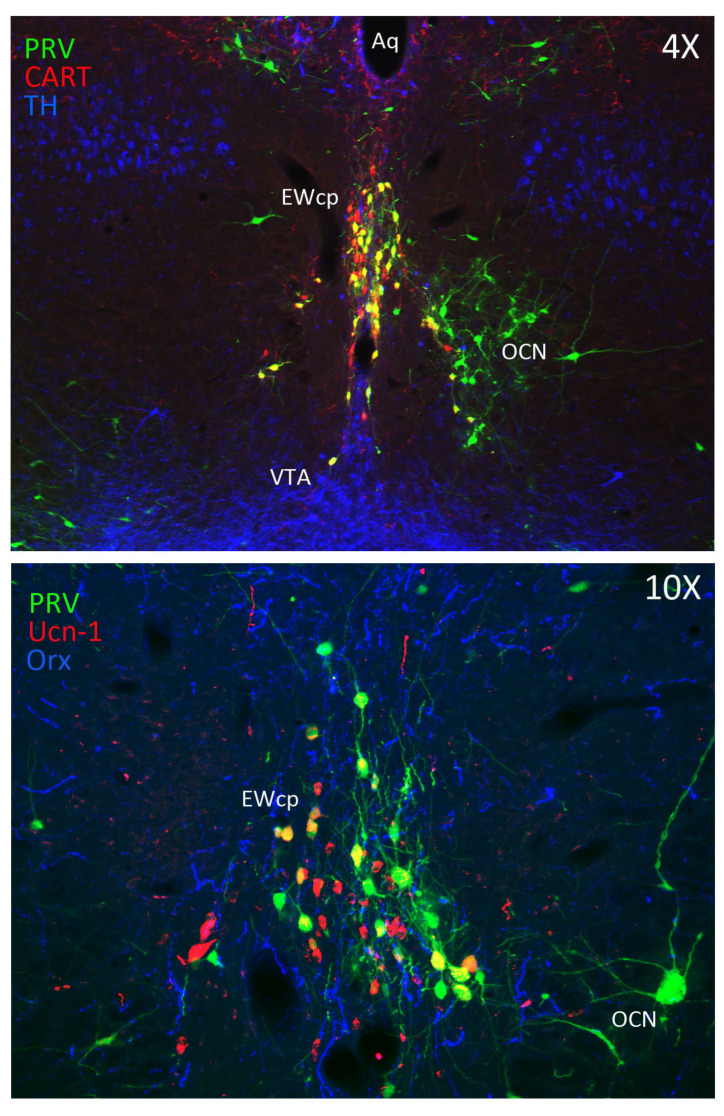
Infected neurons in the EWcp after PRV injection into BAT in rat. Brain sections were processed for triple immunofluorescence. Upper image shows infected neurons labeled in green, CART-ir neurons in red, and dopaminergic neurons (tyrosine hydroxylase-ir) in blue at low magnification (4×). Most infected neurons in EWcp are CART-ir (double-labeled in yellow), intermingled with some non-infected dopaminergic neurons (blue). Lower image shows infected neurons labeled in green, Ucn-1-ir neurons in red and Orx-ir fibers in blue at high magnification (10×). Dense Orx fibers appear in close contact with infected Ucn-1-ir neurons, which are double-labeled (yellow). Bregma level = −6.00 mm (upper image) and −5.62 mm (lower image) based on the rat brain atlas from Paxinos and Watson, 6th ed. [13]. Abbreviations: EWcp, Edinger-Westphal nucleus; Aq, aqueduct; OCN, oculomotor nucleus; TH, tyrosine hydroxylase; VTA, ventral tegmental area.

Besides Ucn-1, EWcp neurons express other neuropeptides. In rats and mice, many EWcp neurons coexpress Ucn-1, cocaine-and-amphetamine-regulated transcript (CART) and nesfatin-1 [4,9]. A separate subpopulation of EWcp neurons colocalize substance P (SP) and cholecystokinin (CCK) [10]. Most CCK neurons in EWcp are glutamatergic as they encode the vesicular glutamate transporter 2 [11], whereas Ucn-1 neurons do not contain it. A subpopulation of the vesicular glutamate transporter 2-containing neurons in EWcp does not express either Ucn-1 or CCK [11]. A small subset of non-Ucn-1 dopaminergic neurons is located in the middle of EWcp and extends ventrally [12] (Figure 1). To date, it is uncertain whether EWcp neurons express markers of GABAergic transmission.

Projections of EWcp neurons have been characterized in several species, most extensively in rats, although several key questions related to the neuroanatomical organization of EWcp remain uncertain. Although studies of afferent inputs to EWcp often use Ucn-1 as a marker for EWcp neurons, the extent to which inputs target subgroups of EWcp neurons based on their projection targets or on their neurochemical phenotype has not been fully addressed. Thus, the degree of heterogeneity of EWcp neurons and their respective projection patterns, as well as the impact of this diversity on the multiple functions attributed to EWcp, is currently unknown. This review focuses specifically on the neuroanatomical connections of EWcp to CNS areas involved in the control of the sympathetic nervous system (SNS) and considers these connections in a functional context, particularly in stress responses and energy homeostasis because both are sympathetic-mediated functions. This review is not intended to systematically cover all functions and anatomical connections of EWcp, as well as the mechanistic features of its interactions with other systems, since these aspects have been reviewed elsewhere.

## 2. Response to Stress and Other Functions of EWcp

EWcp is involved in a variety of diverse functions, such as responses to stress, pain modulation, feeding behavior and addiction. Among them, the most studied is its role in stress responses due to the initial finding that EWcp is the major source of Ucn-1 in the brain. CRF, the main hypothalamic stress-related neuropeptide, and Ucn-1 bind to and activate two G-protein coupled receptors, CRF-R1 and CRF-R2, which have different patterns of expression in the CNS and appear to be involved in different functions. Both CRF and Ucn-1 bind to CRF-R1 with high affinity, whereas Ucn-1 binds to CRF-R2 with much higher affinity than CRF (almost 40-fold higher) [6]. The observation that Ucn-1 is more potent than CRF in stimulating adrenocorticotropic hormone (ACTH) release from cultured anterior pituitary cells [6,14] prompted the suggestion that it may mediate some stress-related functions previously attributed to CRF. Ucn-1 neurons in EWcp in rats are activated (as reflected by increased Fos expression) by various acute stressors, both physical and psychogenic, such as restraint [15,16,17], foot-shock [17], cold exposure [18], dirty cage exchange [19], lipopolysaccharide injection [4,16] and ether exposure [16,20]. Moreover, EWcp activation involves the up-regulation of Ucn-1 mRNA expression by some of these stressors [15,17,20,21].

It is worth noting that although CRF neurons in the paraventricular hypothalamic nucleus (PVN) and Ucn-1 neurons in EWcp are activated by many of the same stressors, they differ in their temporal activation. Thus, Fos expression in CRF neurons in PVN increased in the first hours of stress exposure and declined after 2 h [22], whereas Fos expression in EWcp Ucn-1 neurons peaked at 2–4 h after stress exposure and lasted up to 18 h [15,21]. Cespedes et al. [17] also reported that PVN and EWcp respond differently to the same stressors. In particular, restraint stress increased Fos and CRF mRNA expression in PVN to a greater extent than foot shock, whereas the opposite was noted for Fos in EWcp (increased Fos after foot shock), though Ucn-1 mRNA expression was higher after restraint stress. The increased Fos expression in EWcp evoked by foot shock compared to restraint could be due to the activation of pathways involved in nociception, besides those related to stress per se, since EWcp is activated by pain stimuli (as explained below). Moreover, the observation that Fos in EWcp was increased after foot shock, but Ucn-1 mRNA expression was higher after restraint stress, suggests that neurotransmitters other than Ucn-1 might be involved in the nociceptive responses in EWcp neurons. Another example of the differential response of PVN and EWcp is the effect of chronic benzodiazepine administration, which did not interfere with CRF mRNA expression in PVN, but significantly increased Fos and Ucn-1 mRNA expression in EWcp [17].

Furthermore, Ucn-1 mRNA in EWcp was up-regulated in CRF-knockout mice [15] and down-regulated in CRF-overexpressing mice [23], suggesting a close reciprocal relationship between CRF and Ucn-1 systems. Ucn-1 neurons in EWcp are also activated by chronic stressors without causing habituation, as demonstrated by increased Fos expression [20,24], in clear contrast with the habituating response of PVN CRF neurons to chronic stressors. These data demonstrate that PVN and EWcp do not follow the same pattern of activation during adaptation to chronic stress conditions. Based on these observations, Kozicz and colleagues [8,25] proposed that PVN CRF-neurons and EWcp Ucn-1-neurons constitute two separate, yet functionally complementary systems, which act coordinately during acute stress responses, but are differentially recruited during chronic stress. The authors suggest that Ucn-1 neurons in the EWcp play an important role in stress adaptation, and that the increased Ucn-1 expression may represent a stress-coping mechanism. Moreover, they proposed that the delayed and prolonged activation of EWcp might contribute to the termination of stress responses in order to restore homeostasis after perturbation [8,25,26]. This idea is further supported by the effect of chronic benzodiazepine administration, known to attenuate stress responses, which increased Fos and Ucn-1 mRNA expression in EWcp without affecting CRF expression in PVN [17], as noted above.

When homeostasis has been restored after being perturbed by a stressor, the stress response needs to be terminated (adaptation). When the stress response fails to reestablish equilibrium and/or cannot be terminated, it can elicit stress-related disorders such as anxiety and major depression, which are characterized by maladaptation to chronic stress [8]. Consequently, if Ucn-1 neurons in EWcp are implicated in stress adaptation (termination of stress response), their malfunction may be involved in stress-induced disorders. Consistent with these ideas, brain-wide activity in mice subjected to the learned helplessness procedure, a widely used model of stress-induced depression-like behavior, showed much higher activation of EWcp in “resilient” mice than in “helpless” mice [27]. These observations strongly support the notion that EWcp is involved in stress coping and adaptation. In this context, Ucn-1 seems to be implicated in this adaptation since Ucn-1 knockout mice subjected to a single restraint stress session displayed a normal corticosterone response [28,29,30], but their adaptation to repeated restraint was impaired [30]. Wild-type mice adapted to repeated restraint with a 35% decrease in corticosterone levels, whereas Ucn-1 knockout mice displayed a 75% increase. Impaired adaptation to repeated stress, but not to acute stress, in these mice demonstrates that Ucn-1 has an important role in stress adaptation, including the regulation of the hypothalamic–pituitary–adrenal (HPA) axis.

Central administration of Ucn-1 in rats elicited anxiety-like behavior and increased locomotion [31,32,33,34], suggesting that Ucn-1 is an anxiogenic peptide. However, electrolytic lesions of EWcp in mice had no effect on anxiety-like behavior or locomotion [35,36]. Moreover, Ucn-1 knockout mice did not display decreased anxiety-like behavior and, instead, showed the opposite [28,29]. The discrepancy between the results from centrally administered Ucn-1 studies and those from Ucn-1 knockout mice might be due to the possibility that centrally administered Ucn-1 could activate CRF-R2 as well as CRF-R1, which are not normally accessed by endogenous Ucn-1, resulting in the reported anxiety-like behavior. It has been suggested that Ucn-1 neurons in EWcp may modulate anxiety in opposition to CRF [28]. In agreement with this idea, CRF-R2 knockout mice displayed increased anxiety-like behavior and a hypersensitive HPA axis response to stress, indicated by ACTH and corticosterone peaking 2 min after restraint stress, compared to 10 min. in control mice [37]. Bale et al. [38] have proposed that the absence of CRF-R2 might cause unopposed CRF-R1 activity in these mice, leading to increased anxiety-like behavior and enhanced stress responses. Nevertheless, CRF-R2 knockout mice have an increased number of neurons expressing Ucn-1 mRNA and increased mRNA density in rostral EWcp, which could be responsible for the augmented responses observed [37]. In this context, Coste et al. [39] reported that the initiation of the stress response in CRF-R2 knockout mice was normal, but they displayed early termination of ACTH release, although corticosterone levels remained elevated 90 min after restraint stress exposure. Moreover, stress-coping behaviors associated with decreased arousal, such as grooming in a novel open-field, were reduced in these CRF-R2 knockout mice. These observations suggest that CRF-R2 is involved in the recovery phase of the HPA axis response after stress exposure.

Conversely, ACTH and corticosterone secretion after acute restraint in Ucn-1 knockout mice did not differ from wild-type littermates [29], in agreement with a previous observation in rats that the administration of anti-Ucn-1 serum failed to block the stress-induced secretion of ACTH and corticosterone [40]. These results suggest that although endogenous Ucn-1 is a potent ACTH secretagogue, it does not seem to be involved in the regulation of the HPA axis in response to acute stress or, alternatively, it has a minor or redundant role. However, Smagin et al. [41] reported that central administration of Ucn-1 in rats activated the HPA axis, and this effect was attenuated by administration of antisense oligonucleotides to CRF-R2 mRNA. Nevertheless, in this study, the control rats were injected with a sense oligonucleotide, and it has been reported that central administration of oligonucleotides causes a non-specific (or toxic) activation of the HPA axis [42] as well as fever [43], suggesting that this might be the case in that study.

It is worth noting that Ucn-1 expression in rats seems to be sex-dependent [44], and the majority of Ucn-1 neurons in mice and rats contain estrogen receptor β (ER-β) [45,46]. Haeger et al. [47] reported decreased transcription of the human Ucn-1 promoter with estrogen activation of ER-β (and increased transcription via ER-α activation) in PC12 transfected cells. However, the authors were not able to immunohistochemically detect ER-β in EWcp, the major source of Ucn-1 in the brain [47]. In mice, there is a high density of ER-β in most EWcp Ucn-1 neurons, with no differences in ER-β and Ucn-1 mRNA expression between males and females, although the estrous phase was not considered in this study (43). In rats, BDNF, CART and ER-β colocalize in EWcp neurons (44). There were no differences in CART and BDNF mRNA expression between males and females; however, the numbers of BDNF-ir and CART-ir neurons were 16% and 19% lower, respectively, in females compared to males (44). Derks et al. [44] have shown that there is an absence of ER-α and presence of ER-β in EWcp in rats, using immunohistochemical techniques, and that most Ucn-1 neurons express ER-β. Using QRT-PCR, ER-β mRNA expression in EWcp in female rats in diestrus phase (low estrogen) was found to be 83% and 85% lower than in females in proestrus phase (high estrogen) and males, respectively. In addition, Ucn-1 mRNA expression in females in diestrus phase was found to be 75% and 91% lower than in proestrus stage females and males, respectively, indicating a sex-dependent difference in Ucn-1 biosynthesis closely associated with the estrous phase [44]. Besides the dramatic differences in mRNA expression, the number of Ucn-1 neurons in EWcp and the density of Ucn-1 in cell bodies (measured by immunohistochemistry) did not differ between male and female rats (in both diestrus and proestrus phases). Based on these results, the authors hypothesized that the rate of axonal Ucn-1 transport and secretion might be sex-dependent to the same degree as Ucn-1 biosynthesis is [44]. Since Ucn-1 plays an important role in stress adaptation and estrogens are involved in the control of sex-dependent stress adaptation, the authors proposed that the activity of EWcp Ucn-1 neurons differs between sexes and it is related to estrogen signaling via ER-β activation [44].

These results do not seem to support decreased transcription of the Ucn-1 gene by estrogen activation of ER-β in rats, as reported by Haeger et al. in transfected cells [47]. On the contrary, the results suggest the opposite, because high levels of ER-β mRNA and Ucn-1 mRNA are co-expressed. Considering the neuroprotective effect of estrogens together with the proposed role of increased Ucn-1 expression in EWcp as a stress-coping mechanism, possibly involved in stress adaptation and cessation of stress responses [8,25], then the activation of EWcp Ucn-1 neurons by estrogens would be protective and adaptive. Since ER-β (but not ER-α) are expressed in Ucn-1 neurons in EWcp in rodents, estrogen activation via ER-β probably increases Ucn-1 expression. Therefore, females in diestrus phase, with low estrogen levels and low Ucn-1 and ER-β mRNA expression in EWcp, will be less protected against the deleterious effects of stress, compared to females in proestrus phase (high estrogen levels, high Ucn-1 and ER-β mRNA expression) and males (high Ucn-1 and ER-β mRNA expression).

The sexual dimorphism of Ucn-1 neurons in EWcp might be related to the different stress response strategies adopted by males (‘fight-or-flight’) and females (‘tend-and-befriend’) [48], as well as the higher vulnerability of females to develop stress-related disorders, such as depression and anxiety, possibly because of cycling through low estrogen phases that are associated with low Ucn-1 expression in EWcp. In this context, it has been reported that Fos expression in the EWcp of rats subjected to chronic mild stressors for two weeks was increased only in males [25], further supporting the sex-dependent role of EWcp in stress adaptation. Nevertheless, mechanisms other than ER-β activation might exist in males, leading to increased Ucn-1 expression and its protective effects.

Taken together, the results summarized above provide strong, but incomplete, evidence that EWcp plays an important role in stress responses and stress adaptation.

Pain could be considered in the context of stress responses because acute pain constitutes a warning system to alert the organism towards actual or potential harmful stimuli, whether internal or external. Acute pain triggers an active response to avoid such harmful situations, which includes HPA axis activation as well as the involvement of autonomic, endocrine and behavioral responses. Pain is a powerful stressor, and the body’s response to pain shares some of the main biological mechanisms of the classic stress response.

Evidence suggests that EWcp is involved in the response to acute pain. In early studies, Lantéri-Minet et al. [49] analyzed the expression of several immediate early gene-encoded proteins after noxious visceral stimulation, and reported nociception-evoked overexpression of these proteins in what is now recognized as EWcp. Similarly, EWcp was activated after an acute visceronociceptive stimulus that causes cystitis in rats [50]. Moreover, EWcp neurons that contain CCK were sensitive to noxious stimuli [10] and projected to the trigeminal nucleus [51]. Similar to what has been reported for other stress stimuli, an acute painful stimulus such as formalin injection in the hind paw, induced the sustained activation of Ucn-1 neurons in EWcp, with a peak of Fos expression and up-regulated Ucn-1 mRNA 4 h after stimulus application [21]. In Wistar rats, the same acute pain stimulus caused Ucn-1 peptide content and Ucn-1 mRNA expression in EWcp to peak 2 h after exposure, which returned to control values at 4 h, demonstrating that acute pain stimulated both Ucn-1 transcription and translation [26]. In both cases, the EWcp response was delayed with respect to CRF activation in PVN, and EWcp neurons became activated after corticosterone levels had decreased to control values, leading to the proposal that CRF plays a major role in the initiation of the response to acute pain, whereas Ucn-1 may be involved in its termination phase and adaptation [26].

Similar to other stress responses, when the pain response functions ideally, it constitutes an adaptative trait that protects the organism. Thus, pain alerts the body on a short-term basis, and the organism deals with it for a short period of time. However, when pain persists (e.g., becomes chronic), it turns harmful and maladaptive. The observation that EWcp activation is long-lasting and delayed with respect to CRF-mediated HPA axis activation, together with the notion that EWcp activation might represent a coping mechanism [8,25] and a critical component of the response termination [26], suggests that improper functioning of EWcp could be part of a maladaptive process that leads to the emergence of chronic pain. Nevertheless, it has been reported that a chronic pain model in rats (sciatic nerve constriction for 24 days) produced changes in the activity of the limbic system but not in EWcp neurons [52], suggesting that EWcp may have an intermediary role in establishing chronic pain (by virtue of its dense projections to limbic areas), but not in perpetuating it. We are unaware of any studies to date that have directly examined the role of Ucn-1 or EWcp in chronic pain by manipulating these neurons and assessing pain responses.

EWcp has also been reported to have an important role in feeding behavior, mainly in appetite suppression. Central administration of Ucn-1 potently decreased food intake in both food-deprived and free-feeding rats [6,31,53,54]. Ucn-1 is more potent than CRF in suppressing appetite [31], but much weaker in producing anxiety-like behavior, consistent with a segregation of stress-induced responses between Ucn-1 and CRF [31]. In contrast, Ucn-1 knockout mice and wild-type littermates displayed similar basal food intake and food consumed after 24 h of food deprivation [28,29]. However, CRF-R2 knockout mice showed normal basal feeding and body weight gain, but decreased food intake following 24 h of food deprivation [37], although it is unclear if this was a purely metabolic effect or a stress response to food deprivation.

Ucn-1 and CART colocalize in the same EWcp neurons, and CART is an anorexigenic neuropeptide known to decrease feeding and induce satiety [55]. Thus, the involvement of EWcp in regulating feeding behavior could be mediated by the activation of CART neurotransmission independently of stress activation, or in combination with Ucn-1 neurotransmission during hunger suppression induced by stress. Surprisingly, electrolytic lesions of EWcp significantly reduced food and water consumption in mice that had free access to food and after food deprivation [36], suggesting that other neurotransmitters in EWcp besides Ucn-1 and CART may be involved in feeding behavior.

In response to an acute stressor, there is inhibition of all physiological activities that are non-essential for addressing that stressor (such as feeding, drinking, sleep, reproduction, immune function, etc.) and full activation of those functions that are crucial for mounting a successful fight-or-flight response (increased heart rate and blood pressure, augmented temperature, energy mobilization, etc.). One important event occurring during stress responses is hunger suppression and, in this context, EWcp neurons that contain a potent anorexigenic neuropeptide such as Ucn-1 [31] and are activated by numerous stressors appear to play a significant role. Although this hypothesis is appealing, we are unaware of any studies to date that have specifically addressed the role of EWcp in stress-induced suppression of feeding. Conversely, the role of Ucn-1 neurons in EWcp during normal feeding is unclear because of the lack of studies in freely behaving animals.

EWcp is not only involved in feeding behavior, but it also seems to sense the metabolic state in connection to stress responses. A successful stress response requires energy availability proportional to the nature of the stressor (physical vs. psychogenic; fight-or-flight response vs. adaptation) and duration (acute vs. chronic). In this context, Xu et al. [25] have proposed that EWcp receives and integrates information about stressors as well as about the peripheral metabolic status (glucose, triglycerides, etc.), and orchestrates an appropriate response by activating various neuropeptide systems. These observations suggest that EWcp might be more broadly involved in energy homeostasis in general, rather than in feeding behavior specifically.

Another reported function of EWcp is its role in addiction, particularly in alcohol consumption (for a review, see [5]). Chang et al. [56] first reported that an acute injection of ethanol increased Fos expression in EWcp. Subsequent studies, using a variety of ethanol consumption models in rats and mice, have demonstrated that EWcp is very sensitive to alcohol administration. For example, EWcp expressed Fos after alcohol involuntary administration in rats [12], voluntary consumption in mice [57], and operant self-administration with or without saccharin in rats [58]. In agreement with these observations, c-fos mRNA expression in EWcp was correlated positively with alcohol intake in chronic administration protocols [59], whereas increased Fos B expression in EWcp was observed after 7 days of alcohol free-access in mice [60]. Other drugs have induced increased Fos expression in EWcp in rats and mice, such as heroin [61], morphine [62], cocaine and methamphetamine [63].

Most alcohol-sensitive neurons in EWcp contain Ucn-1 [12,59,64], although in some mouse strains there are non-Ucn-1 neurons sensitive to alcohol [12]. In rat strains selectively bred for different alcohol sensitivities, Ucn-1 levels were higher in alcohol-preferring rats [57]. Similarly, C5BL/6J mice, which drink more alcohol than DBA/2J mice, showed significantly higher levels of Ucn-1 mRNA and higher numbers of Ucn-1-expressing neurons in EWcp [65,66].

Electrolytic lesions of EWcp in mice reduced alcohol intake and alcohol preference in a two-bottle choice [35,65,67,68]. These effects seem to be mediated by Ucn-1 neurons because Ucn-1 knockout mice displayed a similar effect [68]. Deletion of Ucn-1 or CRF-R2 in mice abolished alcohol conditioned place preference, but had no impact on the conditioned aversive effect of alcohol [68]. Ucn-1 seems to mediate the progressive escalation of alcohol intake since Ucn-1 knockout mice displayed reduced alcohol intake and preference when concentrations were increased from 10% to 20% to 40%, but showed no difference with respect to wild-type mice when the concentration remained at 10% [59]. Ucn-1 RNA interference-mediated knockdown via lentiviral injection in EWcp replicated the phenotype of Ucn-1 knockout mice on blunting alcohol intake, demonstrating the absence of developmental compensatory mechanisms in these animals [59].

Although addiction is a complex phenomenon, it can be considered in the context of the original definitions of stress and stress responses. Thus, stress is an internal or external stimulus that threaten the internal milieu, whereas a stress response is a non-specific response of the body to any demand upon it, which is accompanied by HPA axis activation [69,70]. Based on these concepts, the allostasis model of addiction has been proposed [71,72,73]. In this model, addiction is considered as a mechanism for stress adaptation because the external stimulus (drug administration) disrupts the homeostasis of the internal milieu, shifting the biological systems towards an allostatic state away from equilibrium. Subsequently, the body responds to the demand upon it (i.e., mounting a stress response). Biological systems are designed to return to the homeostatic state once the perturbation has ended. However, repeated drug administration (e.g., chronic stress) shifts the system towards the allostatic state over and over, until a point at which the system cannot adjust anymore and adapts to the allostatic state by establishing it as the “new” homeostatic state (e.g., a new “normal”) [71].

Taking together the observations described above, it seems that most known functions of Ucn-1 neurons in EWcp might be in part related, directly or indirectly, to stress responses or stress adaptation. The two main components of the stress response are activation of the SNS and the HPA axis. EWcp may be involved in the central neural pathways orchestrating both responses, but the evidence for its involvement in regulating the SNS is particularly strong. Sympathetic responses to stress involve the coordination of complex brain circuits that modulate sympathetic outflow to organs and tissues via activation of preganglionic and postganglionic neurons in the spinal cord and ganglia, respectively. Within this framework, Ucn-1 neurons in EWcp are well placed to be a key element in the central circuit that modulates SNS activity in stress responses (and most likely in other sympathetic-mediated functions). EWcp receives and integrates information from brain regions involved in multiple functions and conveys the output signal directly to the spinal cord and/or to brainstem presympathetic neurons. In the following sections, we provide evidence for the involvement of EWcp as a significant component in the central control of sympathetic-mediated functions, including metabolic regulation, by examining neuroanatomical and physiological data that support such a role.

## 3. EWcp Connections to Sympathetic Targets

**Figure 2:**
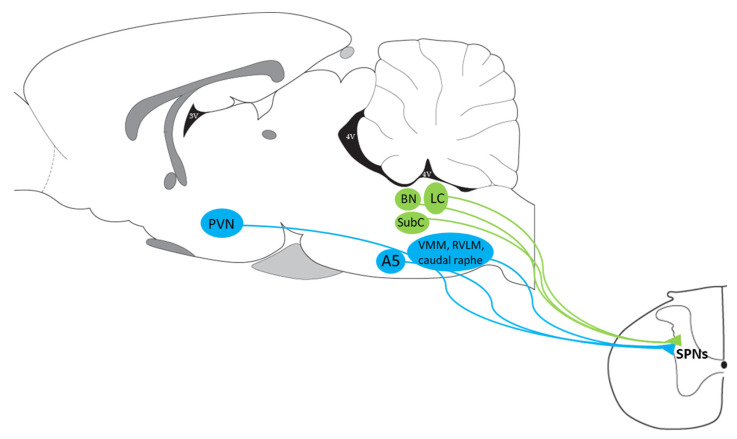
Presympathetic brain regions that project directly to the IML of the spinal cord where SPNs are located. Classical presympathetic areas are depicted in blue, whereas those in green are the regions we suggested to be added based on PRV neuronal tracing. For terms, see the list of abbreviations.

Organs and tissues are innervated by sympathetic postganglionic neurons that, in turn, are innervated by sympathetic preganglionic neurons (SPNs) located primarily in the intermediolateral cell column (IML) in the thoracic and upper lumbar segments of the spinal cord. The activity of these SPNs is controlled by descending projections from brain presympathetic areas, as well as by inputs from spinal interneurons and sensory neurons in the dorsal horn. Typically considered brain areas that directly innervate SPNs (termed presympathetic areas) include the rostral ventrolateral medulla (RVLM), ventromedial medulla (VMM), caudal raphe nuclei (comprising raphe magnus, pallidus (RPa) and obscurus), A5 cell group and PVN. We have previously suggested that the Barrington’s nucleus, the ventral part of the locus coeruleus, and the subcoeruleus nucleus should be added to this list [18,74,75,76] (Figure 2). These presympathetic areas receive and integrate inputs from complex brain circuits and convey multimodal information to SPNs, which ultimately control the sympathetic outflow to efferent organs. In addition, there is an intricate (and inadequately studied) intra-spinal circuit that affects the output of SPNs at the spinal level.

Neuroanatomical data support the notion that EWcp is part of the central network that controls sympathetic outflow to different organs and tissues, probably through connections to presympathetic areas. There are several types of anatomical data that support this: tracing studies of EWcp neuron projections, location of Ucn-1 fibers, location of receptors for Ucn-1 (CRF-R2), and virus-based retrograde trans-synaptic tracing from sympathetically innervated organs and tissues. Classical neuroanatomical tracing studies and the location of Ucn-1 and its receptors supply useful information; however, these observations must be considered as providing indirect evidence because they cannot directly connect EWcp neurons to sympathetic targets. In contrast, virus-based retrograde trans-synaptic tracing from sympathetically innervated targets provides more direct evidence connecting EWcp to sympathetic outflow.

Although the functions of EWcp mentioned in the previous section are the most studied, the anatomical connections of EWcp with other CNS systems unrelated to those functions (i.e., afferents to cerebellum, thalamus, suprachiasmatic nucleus, substantia nigra, lateral septum, superior colliculus, reticular formation, cochlear and vestibular nuclei, etc.; efferents from infralimbic and prefrontal cortex, lateral septum, ventral pallidum, habenula, cerebellum, mesencephalic reticular nucleus, zona incerta, tegmental nuclei, etc.) [77,78,79] strongly suggest that EWcp is involved in numerous functions that have not yet been identified.

The scope of this review is the link between EWcp and sympathetic control; therefore, in the following sections, we describe the neuroanatomical connections of EWcp pertaining to central pathways involved in the regulation of sympathetic outflow. We focused on stress responses and energy homeostasis because the sympathetic component is crucial in both. Considering the vast amount of afferent and efferent projections from and to EWcp described in the literature [77,78,79], those involved in the sympathetic circuitry described below only constitute a small proportion of them.

### 3.1. Evidence Based on Efferent Monosynaptic Tracing Studies

Characterizing the efferent connections of EWcp using anterograde tracer injections has been challenging because of the difficulty with restricting tracer injections into such a small, irregularly shaped nucleus that is intermingled with other neuronal groups. Few studies have addressed this challenge. A complementary approach has been the injection of retrograde tracers in CNS areas suspected of receiving projections from EWcp. Here, we focus on the evidence that such studies have provided regarding CNS regions involved in sympathetic control, primarily the spinal cord and presympathetic brain areas.

In early studies, labeled neurons were observed in EWcp after injection of the retrograde tracer HRP into the spinal cord (lower cervical to thoracic levels) in cats, rats and monkeys [1]. Nevertheless, it could not be established whether the projections from EWcp to the spinal cord terminated on SPNs or upon other spinal neurons. Following this initial observation, Loewy and Saper (1978) [2] characterized the EWcp efferent pathways using autoradiographic anterograde axonal transport of [^3^H]-amino acids after injections into EWcp in cats. Most injections included part of the ventral or ventrolateral periaqueductal gray (PAG) or the ventral tegmental area; thus, these data need to be interpretated with considerable caution. The authors reported a substantial system of descending pathways. Some of the brainstem areas that were shown to receive projections from EWcp contain presympathetic neurons, such as the VMM (including the lateral paragigantocellular nucleus, gigantocellular reticular nucleus (Gi), Gi alpha part, and Gi ventral part), as well as A5, A6 (locus coeruleus) and A7 groups, RVLM and the superior central raphe nucleus (which includes raphe magnus, RPa and raphe obscurus). The authors also reported projections to the medial parabrachial nucleus, which is involved in the control of some autonomic functions [80]. To determine whether these fibers innervated these brain regions or were merely fibers of passage, HRP was injected into raphe magnus or VMM; labeled neurons were observed in rostral EWcp and entire EWcp, respectively, which corroborated the existence of direct projections from EWcp to these brainstem nuclei containing presympathetic neurons. Descending fibers in the spinal cord originating from the region of EWcp were observed near the dorsal horn and appeared to terminate predominantly in lamina I and possibly in lamina V. The authors acknowledged that it could not be determined whether the fibers from EWcp ended preferentially on any specific spinal neuronal type. Surprisingly, no ascending efferent projections from EWcp were observed [2], in contrast to their previous report of a projection from EWcp to PVN and to the results of more recent studies using anterograde tracers [79]. The existence of a direct projection from EWcp to the spinal cord in cats was corroborated by labeling of EWcp neurons after injecting the retrograde tracer HRP into the spinal cord [81].

Further validating some of the observations from Loewy and Saper [2], retrogradely labeled neurons were observed in EWcp after injection of the retrograde tracer cholera toxin subunit B (CTB) in the nucleus reticullaris magnocellularis (analogous to the Gi/Gi ventral part in the VMM, and known to contain presympathetic neurons) in cats [82]. All retrogradely labeled neurons were SP-immunoreactive (-ir) or CRF-ir (the antibody was most likely cross-reacting with Ucn-1, which had not yet been discovered), and some were also CCK-ir. In additional studies, labeled neurons were observed throughout the length of EWcp after large injections of HRP into the cervical and lumbar spinal cord, and the majority were SP-ir [83,84], most of them were CRF-ir [85] and some of them were CCK-ir [51]. All these early studies were performed before Ucn-1 was discovered and, therefore, the proportion of retrogradely labeled neurons that were Ucn-1 in these studies remains unknown; nevertheless, neurons labeled with a CRF antibody in those studies are most likely Ucn-1-ir neurons. Klooster et al. [86] injected the anterograde tracer *Phaseolus vulgaris* lectin L (PHA-L) into EWcp in Wistar rats to identify terminal projections from EWcp to brainstem. Fibers originating from the rostral part of EWcp were observed terminating in several brainstem regions, including VMM and RVLM, which both contain presympathetic neurons. 

More recent studies by Bittencourt and colleagues aimed to systematically describe the efferent targets of EWcp in the brain and spinal cord of Long-Evans rats, using standard tracers in combination with immunohistochemistry for the phenotypic characterization of some projection targets [79]. Injection of the anterograde tracer biotinylated dextran amine (BDA) in EWcp resulted in the identification of multiple ascending and descending projections from EWcp. With respect to areas containing presympathetic neurons, BDA-labeled fibers were observed in PVN, A5 group, subcoeruleus nucleus, Barrington’s nucleus, Gi and caudal raphe (raphe magnus, RPa and raphe obscurus). BDA-labeled fibers were observed throughout the entire rostro-caudal extent of the spinal cord, distributed in the medial part of laminae VII, VIII, and IX, and reaching the ventral part of the central canal in lamina X, which showed the highest density of anterogradely labeled fibers. Some BDA-labeled fibers were also observed in laminae I to IV. Although projections to the IML region (lamina VII), where most SPNs are located, may have been observed, this extensive pattern of spinal projections highlights a potentially broader role of descending EWcp pathways.

Despite the technical challenge of selectively injecting EWcp, all these tracing studies strongly support the existence of projections from EWcp to several presympathetic areas and to the spinal cord. Nevertheless, it is unclear if these fibers establish synaptic connections or are merely fibers of passage, although the studies that include retrograde tracing from some projection targets support the existence of a real projection. Although there are many common findings across these studies, differences are also apparent, likely due to the use of diverse tracing techniques, different species (cat, rat and monkey) or, in the case of rat studies, different strains (Sprague Dawley, Wistar and Long-Evans) that are known to display different neuroanatomical projection patterns (e.g., there are substantial differences in the density of EWcp fibers in different brain regions in pigmented Long-Evans rats vs. albino Sprague Dawley rats [79]. In addition, the different functions exerted by EWcp may be mediated by spatially segregated neurons (e.g., rostro-caudally) and, therefore, anterograde tracer injections at different EWcp levels may render different patterns of projections.

### 3.2. Evidence Based on the Location of Ucn-1 Fibers

EWcp is the main source of Ucn-1 in the brain; therefore, immunohistochemical localization of Ucn-1 fibers has been used extensively to identify EWcp projections in the CNS. Bittencourt et al. [77] described the distribution of Ucn-1 fibers in Sprague Dawley rats, using a Ucn-1 antibody that was affinity purified to eliminate cross-reactivity with other neuropeptides of the CRF family. Among the presympathetic groups, Ucn-1 fibers were observed in PVN, caudal raphe (raphe magnus, RPa and raphe obscurus), VMM (lateral paragigantocellular nucleus, Gi and Gi alpha part), locus coeruleus and Barrington’s nucleus. Other presympathetic regions were not mentioned in that report and, given how the results of that study were presented, it is unclear if they were labeled for Ucn-1. In the spinal cord, Ucn-1 fibers were more abundant in the intermediate and central grey, IML (where most SPNs are located), and ventral horn. These observations support the idea of EWcp being involved in the control of sympathetic outflow via direct projections to SPNs or spinal interneurons. In the same study, injections of the retrograde tracers fast blue or diamidino yellow in the upper thoracic levels of the spinal cord labeled a substantial number of Ucn-1 neurons in EWcp, further supporting the existence of a direct projection from EWcp to the spinal cord. Nevertheless, not all studies have described this pattern of Ucn-1 fiber distribution. Kozicz et al. [87] did not report Ucn-1 descending efferent fibers to the brainstem and spinal cord in CD rats, using a Ucn-1 antibody not commonly employed in most neuroanatomical studies, although dense fibers were observed in forebrain regions such as the lateral septum. Similarly, Morin et al. [88] observed a high density of Ucn-1 fibers in the lateral septum, and scattered fibers in few forebrain regions, but not in the brainstem and spinal cord of Sprague Dawley rats, using a Ucn-1 antibody produced by them.

In mice, Weitemier et al. [89] characterized the distribution of Ucn-1 cells and fibers in two strains, C57BL/6J and DBA/2J. Although Ucn-1 fibers were more abundant in DBA/2J mice, both strains showed fibers in some areas that contain presympathetic neurons such as locus coeruleus, Gi and reticular nucleus (both part of VMM) and raphe magnus. Korosi et al. [90] described the distribution of Ucn-1 fibers in the entire spinal cord (cervical to sacral) in C57BL/6J mice and they noted that in the thoracic cord, where SPNs are located, a low density of Ucn-1 fibers was observed in laminae I-II, V and IX, whereas moderate density was reported in lamina VII (including the IML and intermediate zone) and in lamina X (around the central canal). Since SPNs are located in the IML, intermediate zone and central autonomic nucleus (localized around the central canal), these observations strongly support the idea that Ucn-1 fibers project to SPNs in the mouse spinal cord.

These studies demonstrate the existence of Ucn-1 fibers close to brain presympathetic groups and to SPNs in the spinal cord, strongly suggesting the existence of an anatomical connection between EWcp and the SNS. However, although EWcp is the main source of Ucn-1 in the CNS, it is not the sole source. In the initial characterization of Ucn-1, Ucn-1 mRNA expression in rats was primarily found in the EWcp, and to a lesser extent in the lateral superior olive and supraoptic nucleus [6]. This was corroborated by Bittencourt et al. [77] by in situ hybridization and immunohistochemistry. Moreover, additional brain sites containing Ucn-1 were found in rats pretreated with colchicine (which inhibits axonal transport causing neuropeptides to accumulate in the soma), including PVN, zona incerta, substantia nigra and ventral tegmental area [77]. Kozicz et al. [87] also reported additional Ucn-1 neurons in the supraoptic nucleus, parvicellular PVN, ventromedial hypothalamic nucleus (VMH) and substantia nigra in colchicine-pretreated rats. Similarly, Morin et al. [88] described Ucn-1 neurons in the supraoptic nucleus, lateral hypothalamus, interpeduncular nucleus and sphenoid nucleus in colchicine-pretreated rats. These additional colchicine-dependent sites of Ucn-1 expression, in addition to the EWcp, most likely contribute to the distribution of central Ucn-1 projections observed in these studies. Similarly, in naïve mice, Weitemeier et al. [89] have reported the existence of Ucn-1 neurons in EWcp, lateral superior olive, interstitial nucleus of Cajal and dorsal nucleus of the lateral lemniscus. With respect to the spinal cord, there is a possibility that some of the Ucn-1 fibers observed close to the IML arise from presympathetic neurons in PVN that project directly to SPNs and contain Ucn-1 under certain conditions [77,87,91].

Besides Ucn-1, EWcp neurons contain several neuropeptides, such as CART, CCK, SP and nesfatin-1. It is possible that non-Ucn-1 neurons in EWcp could project to other brain areas different from the ones targeted by Ucn-1 fibers and, therefore, the Ucn-1 immunostaining will be missing these projections. Taken together, these observations suggest that a better approach would be to consider anterograde tracer data in combination with Ucn-1 immunohistochemistry, or retrograde tracing that documents that the only Ucn-1-containing projection to an area is from EWcp.

### 3.3. Evidence Based on the Location of CRF-R2

Ucn-1, which is contained in the majority of EWcp neurons, binds with high affinity to CRF-R2 (in contrast to CRF, which binds with low affinity). The CNS distribution of these receptors might therefore serve as an index of Ucn-1 targets, although an indirect one at best because, as noted above, some other neurons may also express Ucn-1 and Ucn-1 may not be the only endogenous agonist for CRF-R2. Furthermore, CRF-R2 may not be the only receptor for Ucn-1, as Ucn-1 also binds to CRF-R1. The first description of the location of CRF-R2 mRNA in the brain of Sprague Dawley rats [92] reported moderate density in the medial parvocellular PVN, known to contain presympathetic neurons that project to the spinal cord, but it did not describe any CRF-R2 expression in brainstem presympathetic regions. Van Pett et al. [93] characterized the distribution of CRF-R2 mRNA in the brain of Sprague Dawley rats and C57BL/6 and NIH Swiss mice. Surprisingly, among the brain areas that contain presympathetic neurons, the CRF-R2 signal was only detected in the PVN in mice, but not in rats.

A possible explanation for the lack of CRF-R2 mRNA signal in brain sites expected to have them is that the receptors might be located on distal dendrites or presynaptic terminals, very far from the cell bodies that contain the CRF-R2 mRNA. To address this possibility, Tan et al. [94] conducted autoradiographic studies to characterize the location of CRF-R1 and CRF-R2 in the mouse brain and compared with the location of CRF-R1 and CRF-R2 mRNAs from Van Pett et al.’s study [93]. Nevertheless, these studies failed to provide evidence to support this hypothesis because the patterns of mRNA expression and binding site distribution were similar, with very few exceptions, such as CRF-R2 expression in the nucleus of the solitary tract (NTS).

The distribution of CRF-R2 mRNA in the entire spinal cord (cervical to sacral) of C57BL/6J mice was described by Korosi et al. [90]. In the thoracic cord, the highest mRNA density was observed in lamina VII (including the IML and intermediate zone) and lamina X (around the central canal), where SPNs are located. Moderate density was found in laminae IV–VI, VIII and IX, and low density in laminae I–III. The distribution of CRF-R2 mRNA matches with the location of Ucn-1 fibers in these laminae in the same mice. Moreover, immunohistochemical labeling of Ucn-1 fibers and CRF receptors with an antibody that recognizes both CRF-R1 and CRF-R2 demonstrated that Ucn-1 fibers contacted CRF-receptor containing neurons in laminae VII and X, although the neurons were not confirmed to be SPNs [90]. These observations are consistent with the notion that CRF-R2 in the spinal cord may mediate the sympathetic actions of Ucn-1.

Although data from the spinal cord supports the involvement of CRF-R2 in sympathetic control, the location of CRF-R2 in the brain is controversial. Anterograde labeled fibers from EWcp as well as Ucn-1 fibers are located in brain regions that do not express CRF-R2 [77,87], and the restricted CRF-R2 distribution does not provide support for the effects of central Ucn-1. Thus, i.c.v. administration of Ucn-1 elicited widespread activation of cell groups involved in central sympathetic control that only express CRF-R1, such as caudal raphe (raphe magnus, RPa and raphe obscurus) and VMM, or that do not express either CRF receptor, including the central amygdala (CeA), PVN and brainstem catecholaminergic groups such as locus coeruleus, RVLM and caudal ventrolateral medulla [6,95]. In the case of presympathetic regions that only express CRF-R1, Ucn-1 can activate these neurons by binding to them. For brain regions that do not express any CRF receptor, a possible explanation is that the activation of these central sympathetic groups might be secondary to the effects of Ucn-1 exerted directly on brain regions that express CRF-R2 or CRF-R1 and are anatomically connected to the former, such as the lateral parabrachial nucleus (which expresses CRF-R1) and the medial NTS (which expresses CRF-R2) [95]. On the other hand, evidence against the colocalization of Ucn-1 fibers and CRF-R2 in numerous brain regions has led to the suggestion of the existence of a novel CRF receptor subtype not yet identified that binds Ucn-1 [6,96] or, alternatively, the involvement of the CRF-binding protein, which is expressed in the brain and can bind Ucn-1 [97,98]. However, this discrepancy between the location of Ucn-1 and CRF receptors remains unresolved and raises questions regarding the signaling from Ucn-1 neurons in EWcp. Until this is resolved, using the location of CRF-R2 or the effects of centrally administered Ucn-1 as evidence for the involvement of EWcp must be considered with caution.

Taken together, the data based on anterograde and retrograde tracing of EWcp projections, as well as the localization of Ucn-1 fibers, overwhelmingly support the existence of pathways that connect EWcp to presympathetic neurons in the brainstem and possibly to SPNs or presympathetic interneurons in the spinal cord, providing a neuroanatomical basis to support the role of EWcp in sympathetic control. Nevertheless, this evidence is indirect because the synaptic contacts need to be confirmed at the electron microscope level. Most importantly, although the Ucn-1 fibers are in close apposition to neurons located in presympathetic areas, these are heterogenous populations and not all neurons in these regions project to SPNs (e.g., interneurons or brain-projecting neurons are intermixed with spinal cord-projecting neurons).

### 3.4. Evidence from Trans-Synaptic Tracing Studies with Pseudorabies Virus

More direct evidence linking EWcp with sympathetic outflow derives from studies utilizing the trans-synaptic retrograde transport of pseudorabies virus (PRV). PRV is a neurotropic herpesvirus that is transported retrogradely from neuron to neuron across synapses [99]. Due to this specific trans-synaptic passage, an attenuated strain of PRV (PRV-Bartha) has been widely used to characterize CNS pathways that control peripheral organs and tissues. Infected neurons can be identified immunohistochemically at different post-injection survival times, which allows the identification of subsequent steps of infection and permits the delineation of the hierarchical organization of central pathways. Thus, following the injection of PRV into a sympathetically innervated target, the virus is sequentially transported across synapses from postganglionic neurons (located in paravertebral and prevertebral ganglia), to SPNs (mainly in the IML in the spinal cord), to presympathetic neurons in the brain, to neurons that innervate the presympathetic neurons, with approximately 12 h intervals between successive steps of infection.

The trans-synaptic retrograde transport of PRV from many sympathetic-innervated targets has been described in numerous studies. EWcp has been reported to be infected after PRV injection into the adrenal gland [100,101], spleen [74], kidney [75], brown adipose tissue (BAT) [18,102], white adipose tissue (WAT) [103,104], pancreas after vagotomy [105], stellate ganglion (which supplies the sympathetic innervation of the heart) [106,107,108], heart (sympathetic innervation) [109] and tail artery [110]. These results clearly demonstrate that EWcp is part of the CNS circuitry that controls sympathetic outflow to multiple organs and tissues. Infection in EWcp has not been reported after PRV injection into other sympathetically innervated organs, such as the thymus [111] and bone marrow [112,113], most likely because the survival post-injection times used in these studies were short and only allowed infection of presympathetic neurons in the brain.

Based on the temporal profile of infection following the injection of PRV, EWcp does not seem to contain presympathetic neurons, except for one study. Shah et al. [101] reported infected Ucn-1 neurons in EWcp after PRV injection in the adrenal gland at the earliest survival time at which infected neurons were detected in the brain. The authors concluded that these infected EWcp neurons were presympathetic and projected directly to SPNs in the spinal cord. However, the earliest survival time used in this study was 96 h, which is much longer than the early survival times used in the majority of PRV studies. This survival time is long enough to allow several steps of infection in the brain, especially after injection into the adrenal gland, which is directly innervated by preganglionic fibers from SPNs (thus, it involves one fewer step of infection compared to other sympathetically innervated targets). Moreover, the authors reported the earliest infection in the brain not only in EWcp but also in PVN, dorsal raphe, lateral hypothalamus–perifornical area (LH-PeF), dorsomedial hypothalamic nucleus (DMH), posterior hypothalamus, PAG and red nucleus. Except for PVN, none of these brain regions has been considered a classic presympathetic group [76,80,114], further demonstrating that the shortest survival time used in this study was not short enough to distinguish between infected presympathetic and infected higher-order neurons in the brain.

Despite the existence of a direct projection from EWcp to the spinal cord described in several studies using monosynaptic tracers and the presence of Ucn-1 fibers (likely arising from EWcp) in several spinal cord laminae, nearly all PRV studies of sympathetically innervated targets in rats and mice reported that the infection in EWcp is delayed with respect to the infection of presympathetic areas. There are several possible explanations for the temporal progression of PRV infection in EWcp:

(1) The existence of a non-direct multisynaptic pathway from EWcp to the spinal cord via projections to classic presympathetic neurons located in PVN, gigantocellular reticular formation (part of VMM), caudal raphe, locus coeruleus, Barrington’s nucleus, RVLM and A5 group, known to be infected earlier and to project directly to SPNs located in the IML in the spinal cord. Supporting this pathway, Luppi et al. [82] have reported that the main projection targets from EWcp are the VMM groups (Gi, Gi ventral part and lateral paragigantocellular nucleus), and we have detected infected neurons in these groups at the earliest brain infection, especially after BAT or WAT PRV injection [18,104].
(2) EWcp projects to interneurons in the spinal cord, not directly to SPNs. This would explain the delay with respect to infection in brain presympathetic neurons because there would be an extra step of infection in the pathway at the level of the spinal cord. Supporting this possibility, we have observed that SPNs located in the IML become infected first in the spinal cord after PRV injection into several organs. Then, 6–8 h later, infected interneurons appear intermingled with infected and non-infected SPNs in the IML, intermediate zone and central autonomic nucleus [18,74], strongly suggesting that these interneurons become infected via short projections to infected SPNs. Further supporting this possibility, Dos Santos et al. [79] reported that descending fibers from EWcp were denser in lamina X around the central canal, where we have commonly observed infected interneurons [18,74].
(3) A third possibility is that EWcp neurons could have become infected via sparse axonal projections to infected SPNs, which delays trans-synaptic labeling because it is necessary to reach a viral particle threshold to start efficient replication in infected neurons [99]. The three possibilities are not mutually exclusive (e.g., EWcp can project to presympathetic neurons in the brain and to interneurons and/or SPNs in the spinal cord).

**Figure 3:**
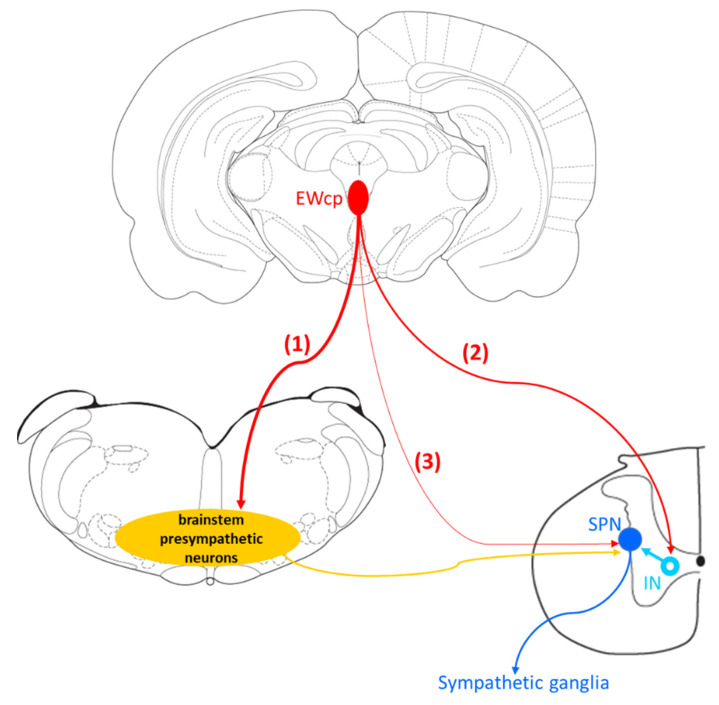
EWcp is part of the central circuit that controls sympathetic-innervated targets. There are several possible pathways for the early infection of EWcp after PRV injection in organs/tissues: (1) via indirect multisynaptic pathway through brainstem presympathetic neurons; (2) via direct projections to interneurons in the spinal cord; or (3) via sparse direct projections to SPNs in the IML. Infection of EWcp neurons at later survival times might include multisynaptic pathways. Abbreviations: IN, interneuron; SPN, sympathetic preganglionic neuron. Coronal section templates were obtained from the rat brain atlas from Paxinos and Watson, 3rd ed. [115].

In summary, results from PRV studies conclusively demonstrate that EWcp is part of the central circuit that controls the SNS outflow to many sympathetically innervated targets, most likely via an indirect multisynaptic pathway through a few brainstem regions, or perhaps via direct projections to interneurons in the spinal cord or sparse direct projections to SPNs (Figure 3). Besides providing direct evidence of the link between EWcp and sympathetic-innervated targets, this technique allows further phenotypic characterization of infected neurons (Figure 1). Moreover, PRV tracing enables the cre-dependent determination of projection patterns when used in transgenic animals.

## 4. EWcp Connections to Sympathetic Targets in Stress Responses

EWcp may serve as a key node in the central neural orchestration of SNS responses to stress. Core sympathetic responses to stress include cardiovascular adjustments (e.g., increased arterial blood pressure and heart rate, mobilization of energy stores, augmented body temperature and suppression of immune function). All these autonomic responses contribute to boosting physical and mental performance, which generate appropriate fight-or-flight responses that promote survival. As described below, there is evidence that EWcp is involved in each of these sympathetic responses.

### 4.1. Cardiovascular Function

**Figure 4:**
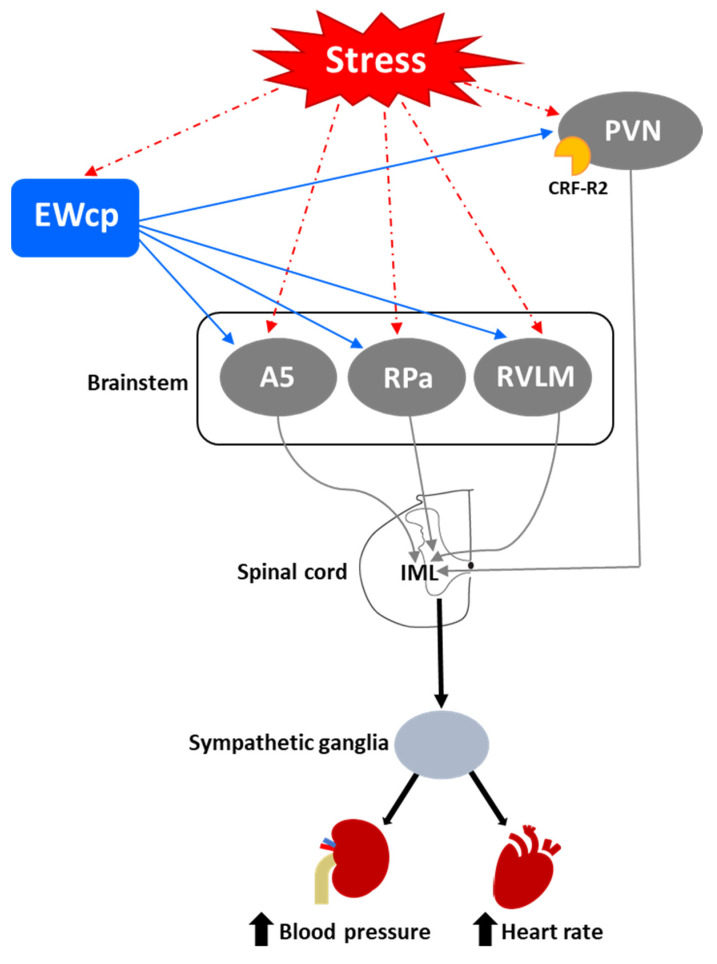
Connections of EWcp to presympathetic regions involved in stress-induced cardiovascular activity. Presympathetic neurons in brain areas involved in cardiovascular regulation, such as PVN, A5, RPa and RVLM, receive inputs from EWcp, which is also activated by stress. The medial parvocellular PVN, known to contain presympathetic neurons, shows a moderate density of CRF-R2. Stress-induced increase in sympathetic outflow to the kidneys and heart increases blood pressure and heart rate. For terms, see the list of abbreviations.

One of the first and most robust physiological effects elicited by numerous stressors is an increase in arterial blood pressure and heart rate. Presympathetic brain areas that are involved in these responses include RVLM, RPa and PVN [116], and these regions receive projections from the EWcp, some of them containing Ucn-1-ir fibers [77,79]. Other presympathetic brain regions involved in cardiovascular regulation (e.g., the A5 group) [117] also receive input from EWcp [79]. The medial parvicellular PVN, known to contain presympathetic neurons, shows a moderate density of CRF-R2 expression in rats [92] and mice [93]. Observations from the temporal progression of PRV infection strongly suggest that EWcp becomes infected via direct projection to these regions (among others) in rats injected with PRV into sympathetically innervated tissues importantly involved in cardiovascular control, including the kidneys [75], heart [109] and stellate ganglion, the site of sympathetic postganglionic neurons innervating the heart [106,107,108]. Thus, the neuroanatomical data provide a substrate for the involvement of EWcp in the cardiovascular component of the stress response (Figure 4). Supporting this hypothesis, increased arterial blood pressure and heart rate have been reported following i.c.v. injection of Ucn-1 [31], or injection of a CRF-R2 agonist into the fourth ventricle [118] or directly into RVLM [119] in rats. CRF-R2 knockout mice have elevated basal arterial blood pressure and diastolic pressure compared to wild-type mice [39], suggesting a role for CRF-R2 in cardiovascular homeostasis.

However, Wang et al. [29] reported that Ucn-1 knockout mice did not show differences in heart rate at baseline, during physical restraint or during recovery from restraint, compared to wild-type mice. Although this may be evidence that EWcp is not involved in stress-induced cardiovascular changes, the lack of an effect of Ucn-1 knockout might be due to several reasons, including differential involvement of Ucn-1 in cardiovascular control among different species, the possibility that these knockout mice might have some developmental compensatory mechanisms, the existence of redundant mechanisms supporting these responses, or the involvement of other signaling molecules from EWcp. Furthermore, air-jet stress in rats elicited increases in arterial blood pressure and heart rate, but was not reported to increase Fos expression specifically in EWcp [120], suggesting a lack of involvement of EWcp in the cardiovascular responses to stress. However, given the number of other stressful stimuli that induce Fos expression in EWcp, the lack of evidence with air-jet stress might reflect that EWcp could have been included as part of PAG, as authors reported increased Fos signal in all PAG subdivisions. Given the clear evidence that presympathetic neurons in brain areas involved in cardiovascular regulation receive inputs from EWcp and that stimulation of CRF-R2 receptors produces cardiovascular responses similar to stress, the involvement of EWcp warrants further attention in this regard.

### 4.2. Glucose Mobilization

**Figure 5:**
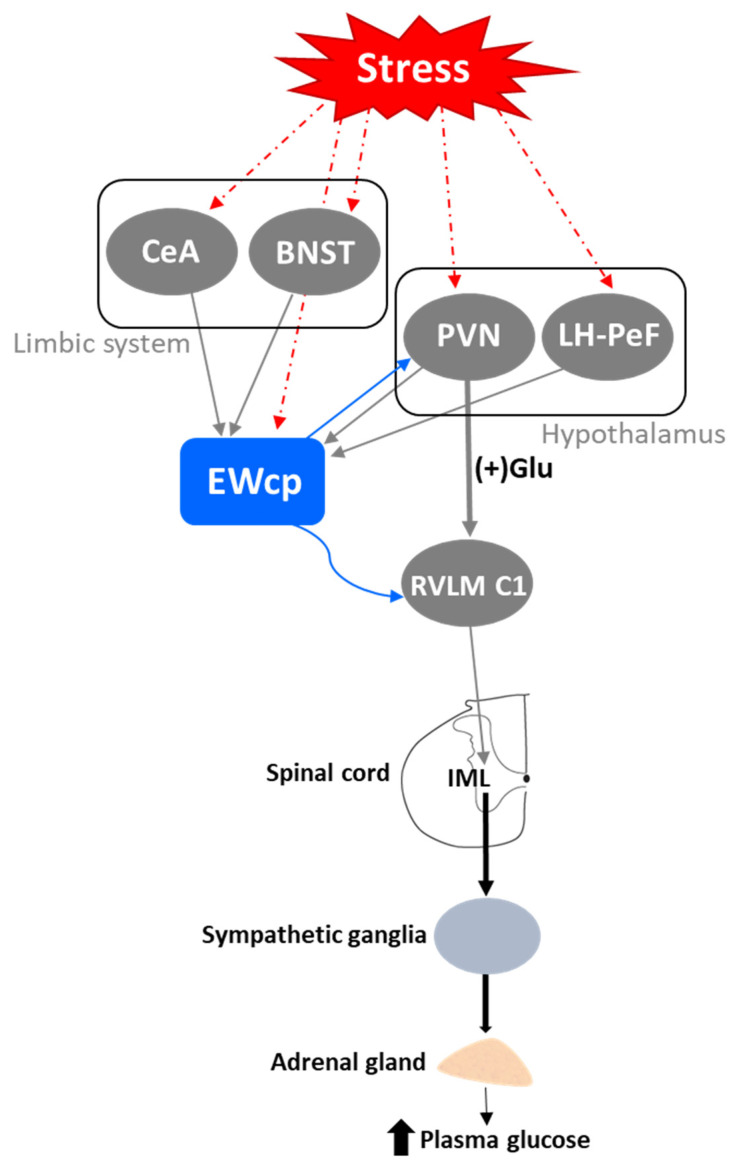
Connections of EWcp with the circuit involved in stress-induced hyperglycemia. Glutamatergic inputs from PVN excite RVLM C1 neurons, inducing glucose mobilization via projections to the spinal cord that activate the adrenal gland (adrenalectomy completely blocks hyperglycemia). EWcp, which is activated by stress, is part of the central circuit that controls the sympathetic outflow to the adrenal gland. EWcp receives projections from limbic and hypothalamic regions that are activated by stress. For terms, see the list of abbreviations.

Another component of the sympathoadrenal response to stress is the mobilization of glucose stores and an increase in blood glucose levels. This response involves the activation of RVLM, particularly the C1 neurons [121,122,123]. It also involves the activation of CRF receptors in the brain, as it is mimicked by central injection of CRF and sauvagine [124,125] and attenuated by a CRF receptor antagonist [126]. The increase in plasma glucose induced by centrally administered CRF is mediated by activation of the SNS as it is completely prevented by pretreatment with a ganglionic blocker [124]. Although the subtype of CRF receptor involved in this response has not been investigated, the greater potency of sauvagine compared to CRF (5–10 times) [125] suggests that the CRF-R2 may be involved. Zhao et al. [123] showed in mice that a variety of inputs, including from glutamatergic PVN neurons, might be involved in exciting RVLM C1 neurons required for evoking stress-induced hyperglycemia. The physical (lipopolysaccharide challenge) and psychogenic stressors (foot shock and restraint) used in this study are known to evoke a strong Fos expression in EWcp [4,15,16,17]. Although the authors did not specifically note EWcp in the list of stress-activated excitatory inputs to RVLM C1 neurons, EWcp projects to RVLM [2,86]; therefore, it is possible that they included it as ventrolateral PAG or that the EWcp projection to C1 neurons is not monosynaptic. The authors reported that the activation of RVLM C1 neurons induces hyperglycemia via descending projections to the spinal cord by virtue of activating the adrenal gland, as adrenalectomy completely blocked hyperglycemia. EWcp is part of the central circuit that controls the sympathetic outflow to the adrenal gland [100,101], and receives projections from brain areas activated by stress, including limbic (CeA and BNST) and hypothalamic (PVN and LH-PeF) regions [78]. Besides their involvement in stress responses, these hypothalamic areas are important modulators of glucose homeostasis, suggesting that the pathway: limbic system/hypothalamus -> EWcp -> RVLM -> spinal cord -> adrenal gland could be a key element of the neural circuit involved in stress-evoked hyperglycemia (Figure 5). Moreover, EWcp is also part of the central circuit that controls the sympathetic innervation of the pancreas [105], suggesting that EWcp could also be involved in the stress-induced sympathetic inhibition of insulin secretion [127], needed for elevating blood glucose levels. 

### 4.3. Hyperthermia

Stress-induced hyperthermia is a physiological response that contributes to increased physical and neural endurance by warming up muscles and the CNS, respectively. Psychogenic stress produces a rapid elevation of body temperature by increasing metabolic heat production and decreasing heat loss from the skin surface, and it is independent of prostaglandin-mediated mechanisms that induce fever. Several psychogenic stressors known to activate EWcp (e.g., dirty cage exchange, foot shock, restraint, social defeat, etc.) evoke a substantial increase in body temperature, which in rodents is mediated by augmented BAT sympathetic nerve activity and BAT activity, as well as cutaneous vasoconstriction [128,129]. β_3_-adrenoceptors mediate sympathetic thermogenesis in BAT [130] and blockade of these receptors decreases stress-induced hyperthermia [128]. 

**Figure 6:**
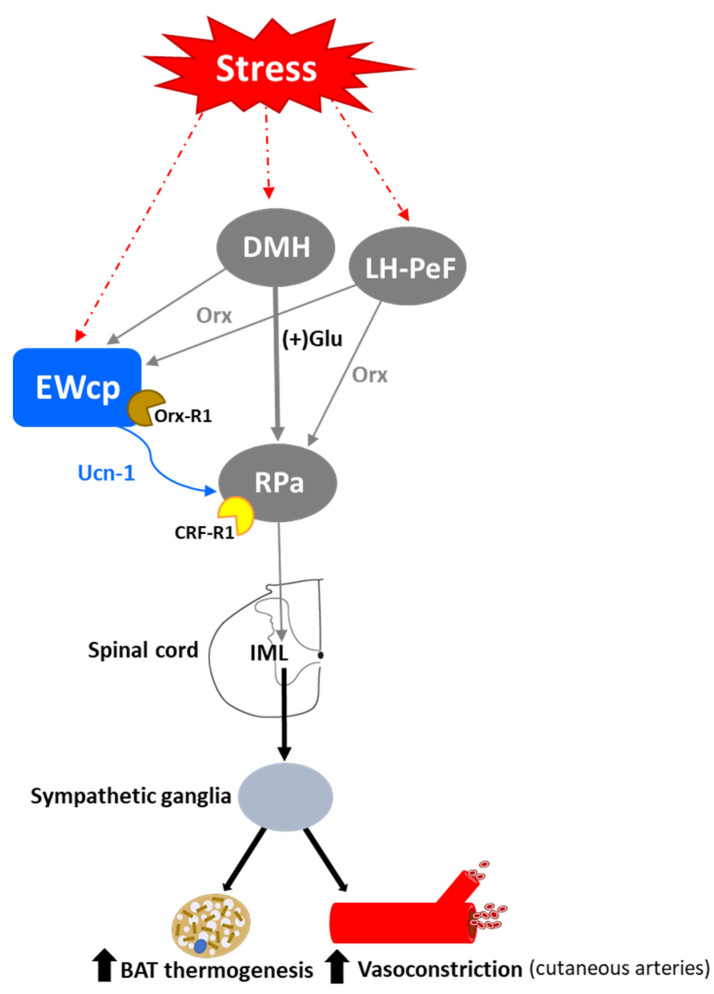
Connections of EWcp with the circuit responsible for stress-induced hyperthermia. Activation of presympathetic neurons in RPa, driven by glutamatergic inputs from DMH, is required for BAT sympathetic nerve activation and subsequent stress-induced hyperthermia. EWcp receives inputs from DMH and projects to RPa, which expresses CRF-R1, receives Ucn-1 innervation (presumably from EWcp), and becomes activated by central administration of Ucn-1. Moreover, stressors that induce hyperthermia activate Orx neurons in LH-PeF and Ucn-1 neurons in EWcp. Orx neurons directly project to RPa and EWcp, which contain Orx-R1. EWcp provides output to brainstem presympathetic regions involved in the sympathetic control of BAT and also the tail artery, which is a key element for cutaneous vasoconstriction in rodents. For terms, see the list of abbreviations.

Activation of presympathetic neurons in RPa, driven by glutamatergic inputs from DMH, is required for BAT sympathetic nerve activation and subsequent stress-induced hyperthermia [128,129]. EWcp receives inputs from DMH [78] and projects to RPa [79]. Furthermore, Ucn-1-ir fibers, presumably arising from EWcp, terminate on RPa neurons [77], which express CRF-R1 mRNA and become activated by central administration of Ucn-1 [95]. PRV injection into BAT demonstrated that RPa becomes infected earlier than DMH and EWcp [18,102], suggesting the existence of direct (DMH -> RPa and EWcp -> RPa) and indirect (DMH -> EWcp -> RPa) pathways that could be involved in the central control of BAT activity during stress responses (Figure 6). In addition, EWcp is also part of the central circuit that controls the tail artery [110], which is a key element for cutaneous vasoconstriction in rodents, also involved in stress-induced hyperthermia.

Stress-induced hyperthermia seems to be potentiated by orexin (Orx), a neuropeptide located in the LH-PeF [131]. Orx is involved in a variety of physiological functions including stress and arousal responses, as demonstrated by activation of Orx neurons and increased Orx mRNA expression evoked by numerous stressors [132,133]. Central administration of Orx augmented body temperature and motor activity [134] and increased Fos in RPa [135], which receives a direct projection from Orx neurons [136]. Activation of Orx neurons induces a long-lasting increase in body temperature via sympathetic-mediated BAT activation [136], whereas Orx injection in RPa (presympathetic neurons) produces a sustained increase in BAT sympathetic outflow and BAT thermogenesis [136]. Moreover, Orx is a key component of the central circuit that controls sympathetic innervation to multiple organs and tissues, as shown in PRV studies [137,138,139,140,141]. These observations demonstrate that Orx is an important element of the central circuit responsible for stress-induced hyperthermia mediated by the SNS via BAT activation. Importantly, Orx neurons are activated by stressors that also activate Ucn-1 neurons in EWcp. Orx-ir axon terminals densely innervate Ucn-1 neurons in EWcp (Figure 1), which express Orx receptor 1 mRNA [142]. Injections of the retrograde tracer FluoroGold into EWcp labeled numerous neurons throughout the rostro-caudal extension of LH-PeF where Orx neurons are located, whereas biotinylated dextran amine injections into LH-PeF showed anterogradely labeled fibers in close contact with Ucn1-ir cells in the EWcp [78]. Besides receiving an orexinergic input, EWcp projects to RPa [79], which is the key component to evoke an increase in body temperature via sympathetic-mediated BAT activation. PRV injection into BAT produces infection of Orx neurons in LH-PeF [137,140,141], as well as in EWcp and RPa [18,102]. These observations suggest the possibility of an additional pathway (Orx (LH-PeF) -> EWcp -> RPa) that could participate in stress-induced hyperthermia.

In summary, stressors that induce hyperthermia activate Orx neurons in LH-PeF and Ucn-1 neurons in EWcp. Orx neurons directly project to Ucn-1 neurons in EWcp, which are part of the sympathetic circuit that controls BAT activity known to be increased by stress. Taking together, these data suggest that Ucn-1 neurons in EWcp are a component of the central circuit responsible for stress-induced hyperthermia by virtue of integrating descendent inputs from areas involved in stress and thermoregulation and providing an output to brainstem presympathetic regions implicated in the sympathetic control of BAT (Figure 6).

### 4.4. Immunosuppression

Stress induces peripheral immunosuppression [143], which can be mimicked via activation of the HPA axis and the SNS by i.c.v. CRF injection [144]. Nevertheless, CRF knockout mice still show a significant stress-induced immunosuppression [145,146], suggesting that another neuropeptide of the CRF family participates in this response, and Ucn-1 is a good candidate. Indeed, i.c.v. injection of Ucn-1 in rats produced a marked decrease in the proliferative activity of splenic lymphocytes [147], which was much more potent than the effect induced by CRF. Central Ucn-1-evoked immunosuppression was mediated by sympathetic activation, and not by HPA axis activation, as it was completely abolished either by pretreatment with a ganglionic blocking agent or by a β-adrenergic receptor antagonist, but not by adrenalectomy [147].

The spleen receives a dense sympathetic innervation, and norepinephrine release from sympathetic fibers inhibits the proliferative activity of splenic immune cells via activation of noradrenergic receptors [148,149]. The increased sympathetic tone in the spleen during stress responses is controlled by a CNS circuit that includes the EWcp, as supported by the infection of EWcp neurons after PRV injection into the spleen [74]. Thus, EWcp is activated by stress and it is part of the central circuit that controls sympathetic outflow to the spleen; in addition, Ucn-1 in the brain exerts a potent peripheral immunosuppressive effect. Therefore, Ucn-1 neurons in EWcp are most likely involved in stress-induced immunosuppression via activation of the SNS.

### 4.5. Emotional Component in Psychogenic Stressors

Activation of CeA and bed nucleus of the stria terminalis (BNST) has been classically considered an important element in initiating stress responses with a psychogenic (emotional) component, such as conditioned fear, dirty cage exchange, foot shock and restraint. Activation of these limbic regions modulates the sensory, motor and autonomic responses associated with affective behavior and stress reactions. In early studies in cats, afferent projections from CeA and BNST to EWcp were identified using retrograde tracing [82]. Later, similar descending projections were also observed in rats [78]. Interestingly, it seems to be a reciprocal connection between CeA-BNST and EWcp. Ucn-1 neurons in the caudal part of EWcp project densely to CRF neurons in CeA, which contain CRF-R1 that Ucn-1 can bind with high affinity [79]. To verify that these were real projections instead of fibers of passage, CTB was injected in CeA, resulting in Ucn-1 neurons in EWcp being CTB-labeled [79]. BNST also receives projections from EWcp [79] and displays CRF-R1 and CRF-R2 mRNA expression in rats and mice [93].

**Figure 7:**
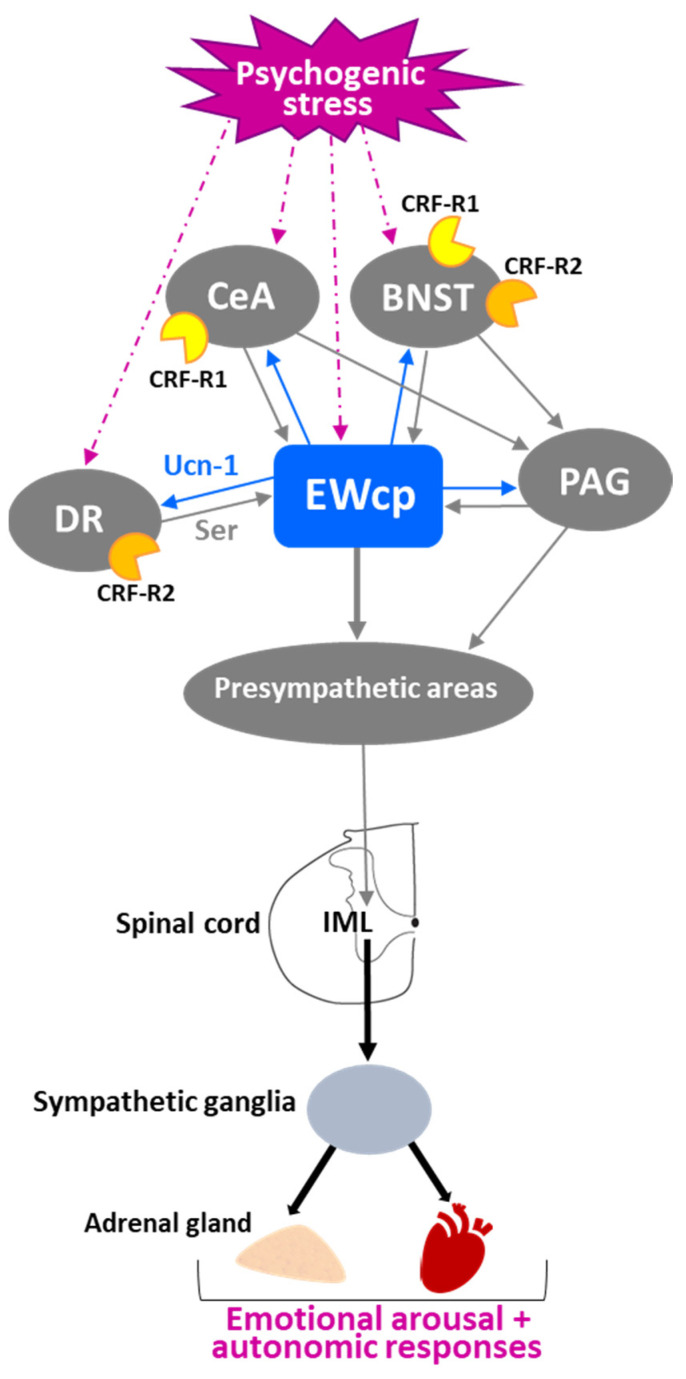
Connections of EWcp with the circuit activated by psychogenic stressors, which evokes emotional arousal and autonomic responses. One of the branches of this circuit comprises the activation of limbic regions (CeA and BNST) that project to PAG that, in turn, projects to brainstem presympathetic neurons that control sympathetic outflow. As with PAG, EWcp receives afferent projections from limbic regions and projects to brainstem presympathetic neurons. This parallelism suggests that EWcp could be a key element of an additional pathway that conveys limbic information to the autonomic brainstem, besides the classic pathway via PAG. EWcp has reciprocal connections with CeA, BNST, PAG, and with serotonergic neurons (Ser) in dorsal raphe (DR), which are activated by psychogenic stressors and express high density of CRF-R2. For terms, see the list of abbreviations.

The central regulation of the SNS depends on structures distributed throughout the brain, including limbic regions such as CeA and BNST. Activation of these regions evokes emotional arousal and autonomic responses to visceral and somatic stress stimuli, as well as to fight-or-flight stress responses. This activation is mediated via connections from limbic regions to hypothalamic areas and/or to PAG that, in turn, project to brainstem presympathetic neurons that control sympathetic outflow. Similar to PAG, EWcp receives afferent projections from limbic areas (CeA and BNST) and projects to brainstem presympathetic neurons that, in turn, project to the spinal cord. This parallelism suggests that EWcp could be a hub of an additional pathway that conveys limbic information to the autonomic brainstem, besides the classic pathway via PAG (Figure 7). Moreover, the ventral PAG and EWcp are located close to each other and their neurons are intermingled at their rostro-caudal boundaries, raising the question of whether some of the sympathetic effects attributed to activation of the limbic–PAG–presympathetic pathway might instead be caused by activation of the limbic–EWcp–presympathetic pathway. Furthermore, the reciprocal connections between EWcp and CeA-BNST [78] could be part of a negative feedback loop to modulate the strength and duration of the response to a stressor. Since EWcp response to stress is delayed with respect to the CRF response in PVN, and EWcp activation has been associated to termination of the stress response rather than initiation (attributed to CRF), the projection from EWcp to CeA-BNST might constitute a pathway to terminate the limbic activation induced by stress. This hypothesis remains to be tested.

Neuroanatomical data support the hypothesis that some functions attributed to ventral PAG might be mediated by EWcp due to their proximity and connectivity. Neurons in EWcp and ventral PAG colocalize at the same rostro-caudal levels and, in some cases, are intermingled. Studies that involved manipulations of this midbrain area, without properly identifying EWcp phenotypically, might have included both neuronal populations or might have affected EWcp more than ventral PAG. With respect to their connectivity, Farkas et al. [107] reported that five PAG regions (dorsomedial PAG, lateral PAG, ventrolateral PAG, EWcp and precommisural nucleus) exhibited infected neurons after PRV injection into the stellate ganglion (which contains the cardiac sympathetic postganglionic neurons). To identify the presympathetic neurons that receive inputs from the PAG, PHA-L injections in lateral PAG or ventrolateral PAG were combined with PRV injection into the stellate ganglion. PRV-infected neurons with PHA-L fibers and terminals in close contact were observed in the VMM (Gi alpha part, Gi ventral part and lateral paragigantocellular nucleus), raphe magnus, RVLM, locus coeruleus, A5 group and PVN. A similar pattern of efferent projections from EWcp to these presympathetic groups was reported after anterograde tracer injections [79], which suggests that some PAG subdivisions and EWcp can be involved in similar functions by virtue of projections to the same brainstem regions. Moreover, Jansen et al. [108] described the intra-PAG circuit in detail, using small PHA-L injections, and demonstrated that the same five PAG regions reported by Farkas et al. [107] (dorsomedial PAG, lateral PAG, ventrolateral PAG, EWcp and precommisural nucleus) were all reciprocally interconnected, and contained infected neurons after PRV injection into the stellate ganglion. In agreement with these findings, Da Silva et al. [78] reported large numbers of retrogradely labeled neurons in all PAG columns after FluoroGold injection into the EWcp. The existence of an intrinsic network of reciprocal connections among all PAG columns, including EWcp, has physiological consequences because the activation or inhibition of one specific column with pharmacological agents or lesions will affect neurons in the other columns and will have widespread effects throughout the entire PAG network. These data support the idea that, in certain conditions, EWcp might act as another PAG column rather than as a distinct nucleus; therefore, some functions attributed to PAG could have been exerted by EWcp. Similarly, caudal EWcp can be confused with the rostral part of dorsal raphe (DR), unless phenotypic characterization is performed, because both populations are intermingled at the same rostro-caudal level (e.g., the initial distribution of leptin receptors in the mouse brain reported by Keshan et al. [150] described the receptors in DR, but later studies demonstrated they are expressed in EWcp neurons). Since DR is involved in psychogenic stress responses and stress-related disorders, such as depression and anxiety, perhaps some effects attributed to DR involvement might be mediated by EWcp. In addition to this possibility, Ucn-1 neurons in EWcp and serotonergic neurons in DR are reciprocally connected [78,79], suggesting that the serotonergic input from DR to EWcp might have some impact on the modulation of sympathetic outflow by EWcp in conditions that increase DR activity, such as exposure to psychogenic stressors (Figure 7). Conversely, Ucn-1 input to DR, which expresses high density of CRF-R2 in rats and mice [93], might affect DR activity when EWcp is activated by stressors. The reciprocal connections between EWcp and DR, both activated by psychogenic stressors, might have a synergistic effect and/or be part of a modulatory feedback loop.

## 5. EWcp Connections to Sympathetic Targets in Thermoregulation

**Figure 8:**
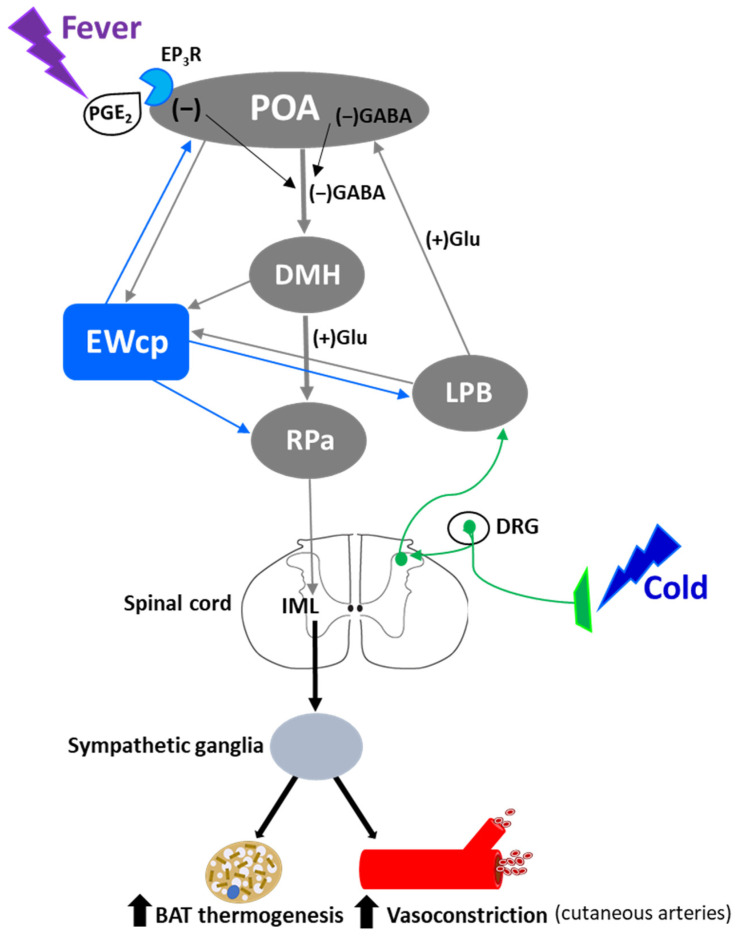
Connections of EWcp with circuits involved in non-shivering thermogenesis and fever. Inhibition of preoptic inputs to DMH, either by excitation from LPB (cold) or by prostaglandin activation of EP_3_R (fever), causes disinhibition of a sympathoexcitatory pathway mediated by DMH and RPa. EWcp receives projections from the main elements involved in these pathways (POA, DMH and LPB), and projects to presympathetic regions that are key elements for the output signal, including RPa. Increased sympathetic outflow to BAT and blood vessels (especially the tail artery in rodents) evokes augmented thermogenesis and vasoconstriction, respectively. Abbreviations: DRG, dorsal root ganglion; EP_3_R, prostaglandin EP3 receptors; LPB, lateral parabrachial nucleus; PGE_2_, prostaglandin E. For the rest of the terms, see the list of abbreviations.

As noted above, EWcp is likely involved in the brain pathways mediating stress-induced hyperthermia. Indeed, the involvement of EWcp in sympathetic thermoregulatory control may be even more general. Following exposure to external thermal stimuli, thermoregulation in mammals is achieved by several autonomic responses coordinated by a complex CNS circuit. These responses include shivering thermogenesis, vasoconstriction/vasodilation and non-shivering thermogenesis. Shivering thermogenesis is mediated by increased contraction and metabolism of skeletal muscles. Vasoconstriction in core vascular beds and vasodilation of blood vessels to the skin allows exchange of heat with the environment. In the skin (and other peripheral tissues such as the tail in rats and mice), vasoconstriction exerted by sympathetic vasoconstrictor nerves reduces blood flow and helps retain heat. Non-shivering thermogenesis is exerted by the modulation of BAT activity controlled by sympathetic innervation, which is increased during cold exposure and blunted by warm ambient temperature (for a review, see [151]). Briefly, during cold exposure, peripheral thermal signals are transmitted by cutaneous thermoreceptors to primary sensory neurons in dorsal root ganglia, which relay the information to glutamatergic sensory neurons in the dorsal horn. This information is transmitted to the lateral parabrachial nucleus (externo-lateral subdivision), which activates GABAergic interneurons in the preoptic area that, in turn, inhibit GABAergic neurons that project to DMH. This results in disinhibition of a sympathoexcitatory pathway that stimulates BAT thermogenesis via DMH glutamatergic activation of presympathetic neurons in RPa, which send descending projections to SPNs in the spinal cord that are involved in BAT innervation [151] (Figure 8).

On the other hand, fever differs markedly from other forms of hyperthermia (stress-induced hyperthermia, exercise hyperthermia, etc.) because it is controlled by a complex thermoregulatory mechanism that is prostaglandin-dependent [152,153]. Fever is an autonomic, endocrine and behavioral response, coordinated by the brain, and mediated by endogenous pyrogens (cytokines) that activate prostaglandin E2 synthesis [154]. Prostaglandin E2 inhibits GABAergic neurons in the preoptic area, by acting on prostaglandin EP3 receptors, which leads to disinhibition of the sympathoexcitatory pathway that stimulates BAT thermogenesis to support the febrile shift of the thermoregulatory set point. This pathway is similar to that activated by cold, involving glutamatergic neurons in DMH that project to presympathetic neurons in RPa [151] (Figure 8). Simultaneously, prostaglandin E2 and other centrally produced mediators stimulate the vasoconstriction of cutaneous vessels to reduce the efficacy of tail skin to dissipate body heat, orchestrating a thermoregulatory response that results in fever [154].

Neuroanatomical data from PRV studies demonstrate that EWcp is a component of the central circuit that controls the sympathetic innervation to BAT, the main organ for heat production in cold defense and fever in rodents [18,102]. Similarly, PRV injection into the wall of the tail artery of rats [110], a major thermoregulatory tissue in this species, or into skeletal muscle [100], also produced retrograde infection in EWcp. These observations suggest that EWcp is strategically placed to coordinate the sympathetic outflow to different organs involved in thermoregulation. Moreover, EWcp receives projections from several subdivisions of the preoptic area and lateral parabrachial nucleus, as well as from DMH [78], and projects to the preoptic area, lateral parabrachial nucleus and RPa [79] (Figure 8), which all are essential components of the central circuit activated during cold response or fever.

Physiological data provide further evidence of the involvement of EWcp in thermoregulation. Mice exposed to acute thermal challenges, either cold (10 °C) or heat (34 °C), show increased Fos expression in EWcp compared to controls at room temperature [64]. Similarly, rats exposed to 4 °C for 4 h displayed increased Fos expression in EWcp [18]. Lipopolysaccharide treatment, known to induce fever, evokes Fos expression in 95% of EWcp neurons that co-express Ucn-1 and CART in rats [4]. Conversely, electrolytic lesions of EWcp in mice significantly blunted ethanol-induced hypothermia [35], consistent with EWcp being part of the thermoregulatory circuit that evokes this response. In addition, EWcp lesioned mice showed a tendency to have lower basal body temperature [35].

Central administration of Ucn-1 in rats increased body temperature [154,155,156,157], which was completely prevented by ganglionic blockade [155], indicating that Ucn-1-evoked increase in body temperature is mediated by sympathetic activation. Pretreatment with a cyclooxygenase inhibitor also prevented the increase in body temperature induced by i.c.v. Ucn-1 administration and attenuated the already existing elevated body temperature when injected 30 min following Ucn-1 administration [156], demonstrating the involvement of prostaglandins (mainly prostaglandin E2) in Ucn-1-induced hyperthermia and suggesting that it involves fever-producing mechanisms. The hyperthermic effect evoked by central Ucn-1 was mediated by CRF-R1 because it was prevented by the CRF-R1 antagonists CRF 9-41 or antalarmin, whereas treatment with the CRF-R2 antagonist astressin 2B was ineffective [157]. Figueiredo et al. [154] reported that central administration of Ucn-1 in rats increased body temperature and was accompanied by elevation of tail skin temperature, an index of peripheral vasodilation. However, in these experiments, this hyperthermic response was not altered by treatment with cyclooxygenase inhibitors, the selective CRF-R1 antagonist antalarmin, or the anti-inflammatory corticosteroid dexamethasone, but it was blocked by the non-selective CRF receptor antagonist astressin. The authors concluded that Ucn-1 acting on CRF-R2 induces a hyperthermic response that is independent of inflammatory mediators, such as prostaglandin E2 and corticosteroids [154]. These results are in apparent conflict with the findings reported in the previous studies [156,157], which concluded that the hyperthermic effect of i.c.v. Ucn-1 administration was mediated by CRF-R1 and was prostaglandin E2-dependent. Nevertheless, procedural differences between the studies (e.g., ambient temperature, drug doses, etc.) preclude direct comparison of their results. Despite the discrepancies between studies regarding the receptor subtype and other mediators involved, it is clear that i.c.v. Ucn-1 increases temperature in a sympathetic-dependent manner.

Mice with genetic manipulations of Ucn-1 or its receptors have also been used to study the role of Ucn-1 in thermoregulation. However, results from studies using such mice must be considered with caution because of the possibility of compensatory mechanisms during development that could mask some functions associated with underexpressing or overexpressing the manipulated gene. Furthermore, all genetic models tested to date have altered gene expression peripherally as well as in the CNS. Nevertheless, data from studies using Ucn-1 or CRF-R2 deficient mice provide relevant information. Ucn-1 knockout mice showed no corticosterone response after 2 h of exposure at 4 °C, suggesting that cold exposure activates Ucn-1 neurons resulting in stimulation of the HPA axis [30]. However, these results suggest that the altered corticosterone response might be related to an impaired stress response rather than to a thermoregulatory effect. Indeed, these knockout mice did not show adaptation to repeated restraint stress since corticosterone levels increased instead of decreasing as observed in wildtype littermates [30].

Bale et al. [38] reported that CRF-R2 knockout mice under basal conditions did not show significant differences in food intake or body composition from wild-type littermates. However, when exposed to repeated cold stress (1 h/day × 15 days), these mice lost more weight and consumed less food than wild-type mice, and at the end of the treatment had decreased body fat and reduced feeding efficiency compared with their wild-type littermates. However, no differences in body temperature were observed before or after cold stress exposure. CRF-R2 knockout mice displayed augmented sympathetic tone to BAT and WAT as demonstrated by the substantial increase in basal uncoupling protein 1 (UCP-1) levels in BAT compared to wild-type mice, as well as by the smaller size of BAT and WAT adipocytes, accompanied with decreased triglyceride content [38]. In addition, CRF-R2 knockout mice showed a robust increase in basal BAT thermogenesis, equivalent to adrenergic-stimulated thermogenesis in wild-type mice, and also displayed little augmentation in response to β3 agonist treatment known to stimulate BAT activity [158]. These results suggest that basal BAT activity in these mice was already maximal, and further support the idea that CRF-R2 knockout mice have increased sympathetic outflow to BAT. Moreover, CRF-R2 knockout mice showed a threefold preference for warmer temperatures when subjected to a behavioral model of differential temperature selection, suggesting the possibility of body heat loss due to increased BAT thermogenesis [158]. Taken together, all these results provide evidence of the involvement of CRF-R2 in adaptive responses necessary for the regulation of energy homeostasis, and also suggest that elevated CRF-R1 activity in the absence of CRF-R2 results in increased thermogenic and metabolic rates, possibly because of larger unrestricted sympathetic outflow [158].

Observations from studies using Ucn-1- or CRF-R2-deficient mice demonstrate the lack of differences in basal conditions, but the existence of remarkable abnormal responses when mice are subjected to stimuli that challenge homeostasis. In addition, these studies validate the involvement of the Ucn-1/CRF-R2 system in thermoregulation via sympathetic mechanisms. Bale et al. [38] have proposed that an important function of the Ucn-1/CRF-R2 system is to counterbalance (or terminate) the action of the CRF/CRF-R1 system on situations that perturbate energy and metabolic homeostasis, and this compensation is exerted by modulating the sympathetic outflow to target organs. In summary, neuroanatomical and physiological data, together with observations from mice with genetic manipulations, support a role of EWcp and the Ucn-1/CRF-R2 system in thermoregulation in different conditions such as cold exposure, fever or other forms of hyperthermia. This involvement is exerted via central control of sympathetic tone to organs and tissues with thermogenic capabilities, such as BAT, WAT, blood vessels to the skin and muscles.

## 6. EWcp Connections to Sympathetic Targets in Feeding Behavior and Metabolism

### 6.1. Effects of Ucn-1 in Food Intake and Metabolic Control

Suppression of food intake was one of the first actions described for centrally administered Ucn-1 in free-feeding and food-deprived rats [6,31,53,54], and the anorexic effect of i.c.v. Ucn-1 was much more potent than that induced by CRF [31]. Low doses of Ucn-1 in rats decreased meal size without affecting meal frequency, similar to the feeding pattern induced by i.c.v. administration of the anti-appetite agent D-fenfluramine [31], suggesting that central Ucn-1 is a selective and specific appetite suppressor. Ucn-1 administered in the third ventricle decreased food intake in rats without producing a conditioned aversion to food, in contrast to CRF, which required a higher dose to produce the same degree of feeding suppression and was accompanied by conditioned food aversion [159]. Ucn-1 administered into the fourth ventricle in rats produced long-lasting feeding suppression, as well as substantial elevations in plasma glucose and corticosterone [53], comparable to i.c.v. administration [53]. In addition to the feeding effects, central Ucn-1 administration promoted energy expenditure by increasing whole body oxygen consumption [155]. Additionally, Ucn-1 injection in the lateral ventricle caused body weight loss at 24 h, cumulative up to 72 h, whereas this effect was modest after fourth ventricle injection [53]. This profile of decreased food intake accompanied by glucose mobilization and increased energy expenditure produced by Ucn-1 is similar to the response evoked by many stressors.

The feeding suppression evoked by central administration of Ucn-1 in rats seems to be mediated by CRF-R2. Pre-treatment with i.c.v. antisense oligonucleotides to CRF-R2 mRNA, but not the administration of a selective CRF-R1 antagonist, attenuated the anorectic response to Ucn-1 [41]. CRF-R2 knockout mice displayed normal baseline feeding behavior, weight gain and plasma lipids [37,38,160], but showed decreased food intake following 24 h of food deprivation (75% of wild-type levels) with no change in body weight after food deprivation or refeeding [37]. However, when subjected to high-fat diet or repeated cold exposure, CRF-R2 knockout mice showed decreased body fat and feeding efficiency compared with their wild-type littermates [38]. CRF-R2 knockout mice consumed significantly more calories than their littermates on a chronic high-fat diet, despite maintaining similar body weight, and were resistant to diet-induced insulin insensitivity associated with increased body fat content, as observed in wild-type mice [38]. Thus, metabolic changes in CRF-R2 knockout mice were observed only after a homeostatic challenge, but not in basal conditions, suggesting that CRF-R2 may have a modulatory role during stress perturbations. Coste et al. [39] reported that although basal food intake was similar in CRF-R2 knockout mice and wild-type littermates, as well as initial feeding suppression following i.c.v. Ucn-1, CRF-R2 knockout mice recovered more rapidly from i.c.v. Ucn-1 than wild-type littermates. These results suggest that the early phase of central Ucn-1-induced hypophagia is most likely mediated by CRF-R1 activation, whereas the late phase depends on CRF-R2, which seems to be essential for maintaining long-lasting feeding suppression [39]. CRF-R2 knockout mice also displayed alterations in energy homeostasis when challenged. Carlin et al. [158] reported that CRF-R2 knockout mice subjected to a chronic high-fat diet exhibited a decreased respiratory exchange ratio (reflective of increased fat metabolism) compared to wild-type mice; this effect was blocked by a CRF-R1 antagonist, suggesting that augmented CRF-R1 activity in the absence of CRF-R2 may result in increased metabolic (and thermogenic) rates, possibly because of greater sympathetic outflow [158]. In contrast to CRF-R2 knockout mice, Ucn-1 knockout mice had normal body weight and basal feeding behavior [161], as well as normal food intake and body weight after 24 h of food deprivation [28,29]. Overall, these results from CRF-R2 knockout mice are generally consistent with the notion that Ucn-1 acting on CRF-R2 contributes to feeding and metabolic responses to stress.

Ucn-1 is also involved in other metabolic responses, including changes in the expression of peripheral mitochondrial uncoupling proteins, known to mediate thermogenesis and influence energy expenditure and metabolism. UCP-1, mainly found in BAT, is considered a marker of BAT activity, whereas uncoupling proteins 2 and 3 are located in WAT and muscle, respectively [162,163]. Decreased UCP-1 gene expression in BAT [164] and increased uncoupling protein 3 gene expression in muscle [165] normally occur in response to food restriction in rats. These effects were reversed by the injection of Ucn-1 into PVN (i.e., UCP-1 gene expression in BAT increased and uncoupling protein 3 gene expression in muscle decreased) [166]. Moreover, in these rats, Ucn-1 injected into PVN increased plasma leptin levels compared to similarly food-restricted control rats [166]. These observations indicate that central Ucn-1 decreases feeding while also preventing at least some of the metabolic responses induced by fasting, such as thermogenesis inhibition and decreased plasma leptin. During food restriction, Ucn-1 injected into PVN increased thermogenesis by elevating BAT UCP-1 levels, a process mediated by increased sympathetic outflow [151]. Kotz et al. [166] noted that the induction of UCP-1 in BAT by Ucn-1 injected into PVN during fasting is consistent with the idea that feeding and thermogenic actions of other central neuropeptides are inversely related, resulting in whole body anabolic or catabolic responses, depending on the stimulus. Supporting this notion, there is a consistent and robust inverse relationship between food intake (and fat storage) and sympathetic activity, mainly in adipose tissues [167]. Neuropeptides and hormones that evoke feeding suppression (such as Ucn-1, CART, CCK, CRF, leptin, etc.) stimulate SNS activity, evoking higher energy expenditure, whereas those that increase food intake (such as NPY, Orx, MCH, ghrelin, etc.) decrease SNS activity and induce body fat deposition. Similarly, drugs that affect food intake (nicotine, caffeine, ephedrine, dexfenfluramine, etc.) have the same inverse effect on SNS activation. Low SNS activity leads to hyperphagia and can cause obesity in the absence of compensatory mechanisms and, reciprocally, most obesities are linked to low sympathetic activity in BAT and WAT. Thus, feeding inhibition by SNS activation seems to be a mechanism that helps to regulate body fat storage [167]. In this context, EWcp is part of this regulatory mechanism via Ucn-1 activation, which suppresses food intake and increases sympathetic nerve activity to BAT and WAT, augmenting thermogenesis and lipolysis, respectively.

Ucn-1-evoked feeding suppression and energy mobilization seem to be mediated by brain regions that receive projections from EWcp, although it must be noted that the site (or sites) of action is not yet certain. Administration of low doses of Ucn-1 into PVN in rats decreased food intake in several feeding paradigms, including the normal nocturnal surge in feeding as well as feeding induced by food deprivation or by neuropeptide Y (NPY) administration [168]. Currie et al. [169] reported that Ucn-1 injection into the PVN in rats at their natural dark-onset suppressed feeding for several hours and decreased the respiratory quotient (V_CO2_ production/V_O2_ consumption), reflecting a shift in metabolism from carbohydrate utilization to favoring fat oxidation [167]. These results suggest that PVN has a major role in mediating the anorectic and metabolic effects of Ucn-1. However, neuroanatomical data provide little basis for endogenous Ucn-1 action in the PVN because there is a low to moderate density of Ucn-1 fibers in different subdivisions of PVN [77] and there is no expression of CRF-R2 in rats [93]. Importantly, Ucn-1 is more potent in eliciting the suppression of feeding when administered i.c.v. compared to direct injection into the PVN, indicating that other brain sites are more sensitive to the anorectic properties of Ucn-1. Indeed, this difference can be explained by the fact that Ucn-1 injected i.c.v. can reach hypothalamic areas that are involved in the control of feeding/satiety responses and express CRF-R2, such as the arcuate nucleus (Arc), which senses metabolic signals and modulates the activity of other brain regions involved in feeding/satiety behavior [170]. Furthermore, Ucn-1 administered into the fourth ventricle still decreased feeding and glucose mobilization following supra-collicular transection, indicating that the PVN is not necessary for these responses [171]. In fact, the observation that injection of Ucn-1 into NTS in rats decreased food intake [53], coupled with the finding that NTS was the only brainstem region displaying increased Fos expression following fourth ventricle injection of Ucn-1 in decorticate rats, makes the NTS a likely site of action. Neuroanatomical data support this idea because there is a moderate density of Ucn-1 fibers [77] and moderate CRF-R2 expression in NTS in rats [93].

Taken together, these data further support a role of Ucn-1 neurons, presumably in EWcp, in feeding and metabolic control. Again, these metabolic actions of central Ucn-1 are consistent with the metabolic responses evoked by many stressors. Available data support the notion that the decrease in food intake mediated by Ucn-1 via CRF-R activation is caused by the stimulation/inhibition of anorexigenic/orexigenic hypothalamic pathways. However, whether this effect is related exclusively to stress responses or might also be independent is difficult to assess because these experimental manipulations (i.c.v. Ucn-1, knockout mice, etc.) do not mimic a real situation in freely behaving animals (i.e., flooding brain regions close to ventricles with Ucn-1 with an i.c.v. injection does not happen naturally).

In the next section, we discuss how EWcp fits in the CNS circuit that controls food intake and energy homeostasis and hypothesize that EWcp might link the well-characterized “hunger–satiety” hypothalamic circuit with brainstem presympathetic neurons that modulate the output to BAT and WAT activity, which ultimately control energy homeostasis.

### 6.2. Involvement of the SNS in Metabolic Control and Energy Homeostasis

The CNS plays a key role in the regulation and integration of food intake, energy expenditure, thermogenesis, glucose mobilization and fat metabolism. The coordination of systems implicated in regulating caloric intake and energy storage vs. energy utilization involves central neural circuits that can regulate all these parameters. Control of adipose tissue activity, both WAT and BAT, is essential to this. After integrating information about environmental conditions and the animal’s energy state, these interconnected neural pathways have to simultaneously coordinate sympathetic outflow to both BAT and WAT, as they are critical effectors of the output response. For example, during cold exposure, sympathetic stimulation of BAT induces heat production, using circulating triglycerides and glucose as fuel [130]. In times of energy demand, sympathetic stimulation of WAT induces lipolysis and the energy stored in white adipocytes is mobilized into free fatty acids that generate ATP [172]. Conversely, BAT thermogenesis can be activated after excessive caloric intake or when dietary fat exceeds the normal required amounts, helping to maintain metabolic homeostasis and prevent obesity. Indeed, reduced BAT sympathetic tone and decreased energy expenditure contributes to body weight dysregulation and obesity [173]. In this context, EWcp is not only part of these coordinated circuits, but it also regulates the sympathetic innervation of BAT and WAT. As expanded below, EWcp plays an important role in the maintenance of energy homeostasis under conditions that require the adjustment of energy expenditure by virtue of modulating food intake, thermogenesis in BAT, lipolysis/lipogenesis in WAT, and glucose mobilization/storage (including the regulation of insulin secretion from pancreas).

**Figure 9:**
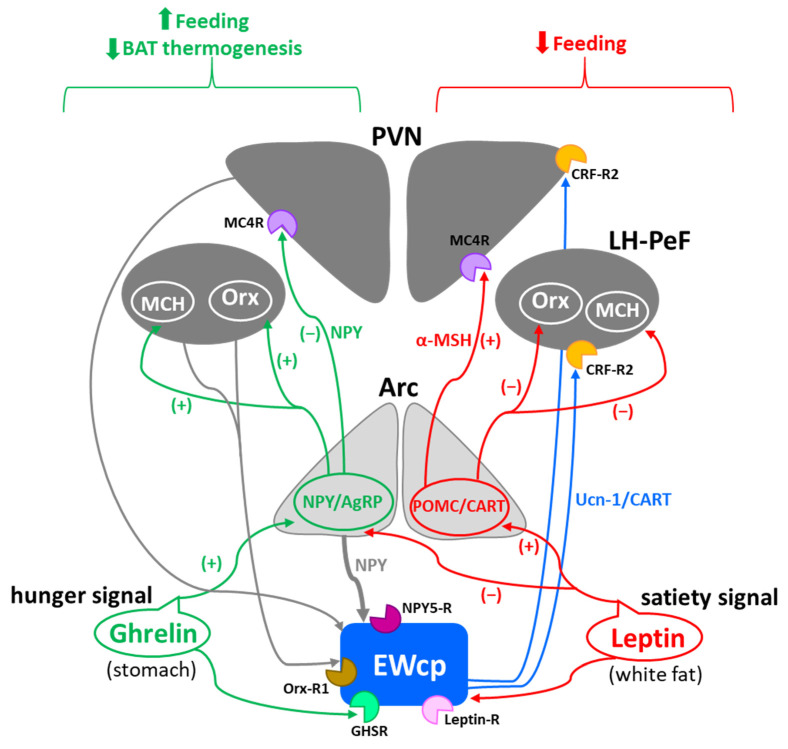
Connections of EWcp with the hunger–satiety circuit. EWcp receives a substantial projection from Arc and has reciprocal connections with PVN and LH-PeF. EWcp neurons express receptors for leptin (satiety signal) and ghrelin (hunger signal), and for the main neurotransmitters involved in this pathway (NPY, Orx, and probably MCH), suggesting that it could modulate energy expenditure by virtue of interacting with key elements of the hunger–satiety circuit. For the sake of clarity, hunger and satiety pathways are depicted unilaterally, as well as EWcp afferent and efferent projections, although all these pathways are bilateral. For terms, see the list of abbreviations.

Feeding depends on food availability and the animal’s nutritional state, and this information is conveyed to the brain by peripheral signals through humoral and sensory pathways. These signals are integrated by the hypothalamus, which generates a “hunger” or “satiety” response, and the resultant output is conveyed to brainstem circuits that regulate the autonomic (sympathetic input to BAT, WAT and pancreas) and behavioral responses (actively seeking food, somatic motor input to masticatory muscles for food consumption, etc.) [172,174]. Briefly, key components of the hypothalamic circuitry controlling feeding include the following elements. Ghrelin, a hormone released from the fasted stomach, conveys an “empty stomach” signal to the hypothalamic arcuate nucleus (Arc), located close to the median eminence, and activates NPY/agouti-related peptide (AgRP) neurons in Arc that project to PVN and LH-PeF. Both NPY and AgRP elicit a “hunger response” by increasing appetite and food intake via inhibition of PVN neurons that express melanocortin-4 receptors (MC4R) [175,176] and activation of melanin-concentrating hormone (MCH) and Orx neurons in LH-PeF. These mechanisms ultimately decrease energy expenditure (through inhibition of BAT thermogenesis) and stimulate feeding. In contrast, in well-fed animals, the WAT-derived hormone leptin (released in proportion to the amount of peripherally stored fat) conveys a “satiety” signal to Arc, activating pro-opiomelanocortin (POMC)/CART neurons that project to PVN and LH-PeF. Leptin also inhibits NPY/AgRP neurons in Arc previously activated by ghrelin. Both α-melanocyte-stimulating hormone (α-MSH) (generated from POMC cleavage) and CART inhibit food intake via activation of PVN and inhibition of MCH and Orx neurons in LH-PeF. This α-MSH satiety signal is mediated by MC4R. NPY from Arc can inhibit MC4R-expressing PVN neurons, counteracting the excitatory effect of α-MSH [176]. Each of these components of the hypothalamic hunger–satiety circuit is anatomically connected to EWcp, strongly supporting its involvement in the control of feeding behavior (Figure 9).

Although the hypothalamic systems involved in feeding and metabolic control have been extensively characterized, less is known about the pathway(s) that conveys the energy status signals to presympathetic neuronal groups that regulate the sympathetic outflow to effector organs, which ultimately control metabolism and energy expenditure. Nakamura et al. [177] have described a central pathway that conveys “hunger” signals from the hypothalamus to brainstem presympathetic neurons and motoneurons in mice, thus coordinating autonomic and feeding motor systems to inhibit BAT thermogenesis and promote feeding under conditions of caloric intake and storage. The authors proposed that during fasting, NPY released from Arc neurons activates a group of PVN neurons (that do not express MC4R). These neurons transmit excitatory signals to NTS, which provides excitatory inputs to GABAergic neurons in the intermediate/parvicellular reticular nuclei in the medulla. Stimulation of these neurons, which project to presympathetic neurons in raphe nuclei, evokes inhibition of BAT thermogenesis, and also activates glutamatergic neurons that elicit mastication and promote feeding via direct projections to the motor trigeminal nucleus [177].

**Figure 10:**
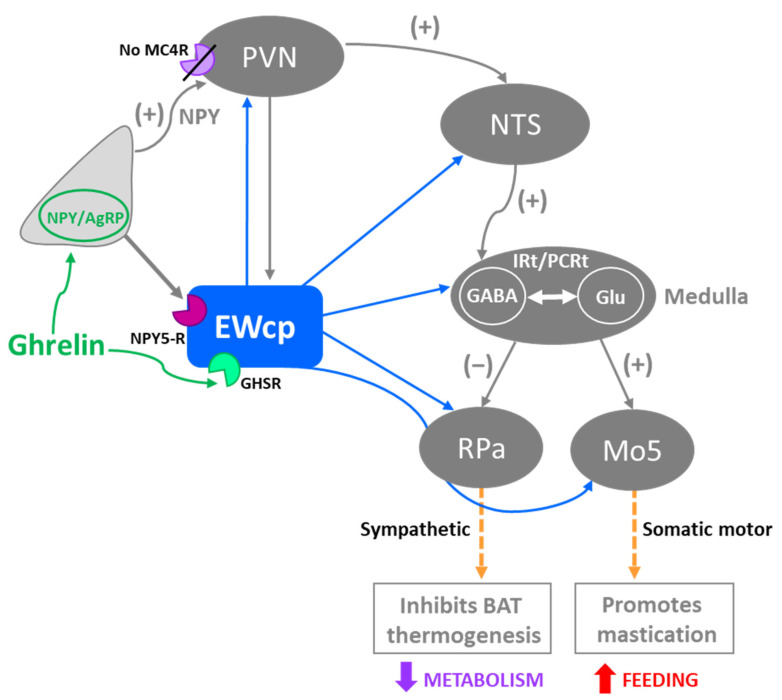
Connections of EWcp with the circuit proposed by Nakamura et al. [177]. The proposed circuit conveys hypothalamic “hunger” signals to brainstem presympathetic neurons and motoneurons to coordinate sympathetic and somatic motor systems to inhibit BAT thermogenesis and promote feeding, respectively, under conditions of caloric intake and storage. EWcp expresses ghrelin receptors (GHSR) and itis directly connected to all brain regions involved in the proposed circuit, suggesting that it might be part of a putative feedback circuit that terminates this response, since one of the main functions of EWcp is feeding suppression. Abbreviations: IRt/PCRt, intermediate and parvicellular reticular nuclei; Mo5, motor trigeminal nucleus. For the rest of the terms, see the list of abbreviations.

We hypothesize that EWcp might be a component of the “hunger” pathway proposed by Nakamura et al. [177]. In particular, EWcp receives direct projections from Arc and from all parvicellular subdivisions of PVN [78], and projects to the intermediate/parvicellular reticular nuclei, as well as to NTS [79]. In addition, moderately dense Ucn-1 projections, presumably from EWcp, were observed in all subdivisions of the raphe nuclei [77], which contain preganglionic neurons known to control BAT activity [151] (Figure 10). With respect to nuclei involved in orofacial control, dense Ucn-1 projections were observed in the facial and spinal trigeminal nuclei, as well as moderately dense projections in somatosensory groups related to the trigeminal nerve (mesencephalic, principal sensory and spinal), and also in motor nuclei of the trigeminal and facial nerves [77]. Unfortunately, PRV tracing studies from orofacial muscles are inconclusive with respect to EWcp involvement because injections were only performed after the tissue was sympathectomized. Thus, PRV injection into the submandibular gland demonstrated that EW (most likely EWpg) is involved in the control of salivation via polysynaptic projection [178]. However, EWpg infection was not reported after PRV injection in other orofacial muscles controlling the lingual musculature [179] or the masseter muscle involved in mastication [180] after sympathetic denervation. 

EWcp might be part of additional pathways that control energy homeostasis under certain conditions. EWcp is ideally placed to be an intermediary node in the neural circuit that conveys the “hunger” and “satiety” signals from the hypothalamus to brainstem presympathetic neurons (or to the spinal cord), which control the sympathetic outflow not only to BAT but also to other organs involved in energy homeostasis such as WAT, adrenal gland and pancreas. In addition, neuroanatomical data (i.e., existence of Ucn-1 fibers) suggest that EWcp might also innervate and regulate the activity of motoneurons involved in chewing and feeding behavior. We propose that EWcp receives and integrates multimodal information (thermal, metabolic, circadian, stress, etc.) via direct afferent projections from various hypothalamic and forebrain regions and modulates the sympathetic output to effector organs to maintain energy homeostasis in different situations. In the next sections, we review anatomical and physiological data in support of this hypothesis.

### 6.3. EWcp and Ucn-1 Are Part of the CNS Circuit That Controls Sympathetic Outflow Involved in Metabolism and Energy Homeostasis

As already noted, studies using PRV to trace multisynaptic neuroanatomical connections have demonstrated that EWcp is part of the CNS circuit that controls the sympathetic innervation of BAT and WAT [18,102,103,104]. However, it is unknown if the same EWcp neurons receive and integrate inputs from diverse brain regions or, conversely, different EWcp subpopulations receive discrete inputs from distinct brain nuclei. Nevertheless, neurons in multiple brain regions are double-infected after the simultaneous injection of two different isogenic PRV in BAT and WAT in hamsters and rats [104,181,182] including the majority of Ucn-1/CART neurons in EWcp [141], demonstrating that EWcp Ucn-1/CART neurons are involved in the control of both types of adipose tissue simultaneously. These observations provide an anatomical basis for the coordination of BAT and WAT activity, EWcp being among the brain regions involved in the circuitry contributing to this coordinated control.

Another important element in metabolism is glucose availability, which depends, in part, on plasma insulin levels. Insulin secretion from the pancreas is influenced by the autonomic nervous system; thus, feeding-induced parasympathetic activity stimulates insulin secretion, whereas stress-induced sympathetic activation inhibits it [127]. EWcp is also part of the central circuit that controls the sympathetic innervation of the pancreas [105]; therefore, EWcp might be able to modulate the pancreas activity, including insulin secretion, under specific conditions. Similarly, EWcp is part of the central circuit that controls the sympathetic outflow to the adrenal gland [100,101]. The adrenal medulla produces most of the body’s epinephrine, which plays an important metabolic role in mobilizing energy stores (glucose and free fatty acids) to recover from hypoglycemia and in preparation for glucose demands during physical activity [183], in addition to other actions of adrenomedullary catecholamines. Epinephrine secretion from the adrenal medulla to counteract hypoglycemia is mediated by the activation of presympathetic hypoglycemia-responsive neurons in RVLM that project to adrenal SPNs in the spinal cord [184]. EWcp projects to RVLM, although it is unclear if it innervates the hypoglycemia-responsive neurons or other subpopulations of RVLM neurons, such as the barosensitive neurons. All these data support a potential role for EWcp in metabolic control in general, through the modulation of sympathetic output to several organs known to be involved.

In addition to EWcp, studies using PRV to trace neuronal pathways connected to BAT, WAT, adrenal gland and pancreas have demonstrated that brain regions known to be crucial in the control of feeding and metabolism (e.g., Arc, PVN, LH-PeF, etc.) also became infected and, therefore, are part of the CNS circuit controlling these tissues. However, it cannot be inferred that these hypothalamic nuclei became infected by direct projections to EWcp because several presympathetic neuronal groups were also infected simultaneously or earlier, providing several possible routes of infection. Nevertheless, studies with monosynaptic tracers demonstrated that most of these areas involved in metabolic control are directly connected to EWcp, in some cases bidirectionally [78,79].

#### 6.3.1. Connections with the Lateral Hypothalamus: MCH and Orx Neurons

As noted above, one brain area that is critically involved in food intake and energy homeostasis is the LH-PeF, particularly neurons that contain MCH or Orx as neurotransmitters (for a review, see [132,185]). MCH is a potent orexigenic neuropeptide that increases food intake and weight gain (mainly fat mass) (for a review, see [185]). Orx neurons become activated and promote food-seeking behavior during prolonged fasting, restricted feeding and hypoglycemia. Once food intake occurs, Orx neurons become inactive (for a review, see [132,186]). LH-PeF is reciprocally connected with EWcp. Injection of a retrograde tracer into EWcp labeled numerous neurons throughout the rostro-caudal extension of LH-PeF [78], whereas anterograde tracer injections into LH-PeF showed labeled fibers in EWcp in close contact with Ucn-1 neurons [78]. In a reciprocal manner, retrograde tracer injections into LH-PeF labeled Ucn-1 neurons in EWcp [79], whereas moderate densities of Ucn-1 fibers were observed in LH-PeF [77]. Moreover, LH-PeF neurons express moderate amounts of CRF-R2 mRNA in rats and mice [93]. Dense anterograde-labeled fibers from EWcp were found in close apposition to MCH neurons in LH-PeF [79]. Furthermore, Able et al. [187] reported MCH receptors in PAG and in reunions nucleus in rat, although these receptors appear to be in EW instead. In addition, Orx neurons from LH-PeF project to EWcp, and Orx fibers were shown to form dense plexuses around EWcp Ucn-1/CART neurons that express Orx receptor mRNA [142]. Furthermore, infected MCH and Orx neurons were observed in LH-PeF after PRV injection into BAT and WAT in rats and hamsters [137,141,188,189], although it is still unknown whether these particular neurons were infected via projections to EWcp or to other infected areas. Nevertheless, given the extensive reciprocal projections between both regions, it is most likely that these neuronal groups are components of the same circuits (Figure 9).

These neuroanatomical data suggest that MCH and Orx neurons in LH-PeF might modulate the activity of Ucn-1 neurons in EWcp involved in the control of food intake by direct activation of MCH and/or Orx receptors. Since Ucn-1 colocalizes with CART and nesfatin-1 in EWcp neurons, these projections can also modulate the release and activity of these neuropeptides. The reciprocal innervation between LH-PeF and EWcp might be part of a regulatory feedback loop under certain conditions such as stress responses or fasting.

#### 6.3.2. Connections with the Arcuate Nucleus: POMC/CART and NPY/AgRP Neurons

As noted above, another brain region established to play a key role in energy homeostasis is Arc [170], particularly POMC and CART neurons, which inhibit appetite and food intake via activation of PVN and inhibition of MCH and Orx neurons in LH-PeF. Another subpopulation of neurons in Arc coexpress the orexigenic neuropeptides NPY and AgRP, which increase appetite and food intake via inhibition of PVN and activation of MCH and Orx neurons in LH-PeF [170] (Figure 9). As demonstrated by retrograde labeling from EWcp, many Arc neurons directly project to EWcp [78]. Gaszner et al. [190] reported the existence of close appositions between NPY terminals and Ucn-1 neurons in rat and human EWcp. In addition, most Ucn-1 neurons expressed NPY receptor Y5 (NPY5R), whereas a few Ucn-1 neurons expressed the Y1 receptor [190]. Moreover, NPY i.c.v. administration induced strong Fos expression in Ucn-1 neurons, as well as the up-regulation of Ucn-1 mRNA in EWcp 2 h after NPY injection. Thus, NPY seems to exert a stimulatory action on Ucn-1 neurons in EWcp via NPY5R receptor activation. Although a projection from Arc to EWcp is clear, a reciprocal connection from EWcp to Arc does not appear to exist, based on the absence of anterograde labeling in Arc from EWcp [79] and also the paucity of Ucn-1 fibers in Arc [77]. These observations suggest the existence of a projection from Arc to EWcp, which could modulate the anorectic response evoked by Ucn-1.

Besides its involvement with appetite and food intake, NPY from Arc seems to be implicated in the regulation of adipose tissue activity. In rats, sustained but modest overexpression of NPY in Arc neurons caused excessive body weight gain and visceral fat deposit, as well as hyperleptinemia [191]. Moreover, it also altered the WAT phenotype by increasing the percentage of large vs. small adipocytes and by decreasing the levels of the differentiation adipogenic marker PPAR-γ2 [191], suggesting dysfunctional adipocytes. Adipogenesis is regulated via sympathetic signaling to adipose tissue, and EWcp is part of the central circuit that controls that pathway. As mentioned above, EWcp receives a direct projection from Arc, expresses NPY5R receptors, and it becomes activated by NPY. These observations suggest that the connection Arc (NPY) -> EWcp (NPY5R) could be involved in the modulation of the sympathetic tone to WAT, which will impact its activity.

With respect to BAT, Shi et al. [192] demonstrated that there is a functional relationship between NPY signaling from Arc and BAT thermogenesis via modulation of sympathetic outflow. The authors generated a mouse model in which NPY was selectively reintroduced into the Arc of NPY-deficient mice. This manipulation decreased BAT temperature and markedly reduced (threefold) BAT UCP1 mRNA expression, which could be reversed after surgical sympathetic denervation of BAT. Catecholaminergic downstream targets of Arc NPY signaling showed decreased tyrosine hydroxylase mRNA and protein expression in PVN, locus coeruleus, NTS and RVLM (C1/A1 neurons). The authors suggested that reduced catecholamine synthesis in these brain regions could lead to diminished sympathetic outflow to BAT and, consequently, decreased thermogenesis [192]. EWcp receives a projection from Arc and projects to all these presympathetic catecholaminergic groups; thus, EWcp could be part of this circuitry that conveys NPY signaling from Arc to modulate sympathetic outflow to BAT and, therefore, regulate its activity.

With respect to other neuropeptides expressed in Arc neurons, POMC is a precursor of several cleaved products (melanocortins), which can bind to five G-protein coupled receptors (MC1R–MC5R) [193]. α-MSH is the most potent anorexigenic neuropeptide of the melanocortin system and it is the primary endogenous agonist of MC4R [194], whereas AgRP is orexigenic and acts as an endogenous antagonist of MC4R [195]. MC4R activation by α-MSH decreased fat stores by reducing food intake and increasing energy expenditure. Conversely, mice lacking MC4R displayed severe obesity, hyperphagia, hyperglycemia and blunted thermogenic responses to increased dietary fat [196,197,198]. Recovery of MC4R signaling in PVN neurons attenuated food intake in obese MC4R knockout mice, but energy expenditure was not restored, suggesting that other brain regions are responsible for this effect [198] and that divergent melanocortin pathways control food intake and energy expenditure through MC4Rs signaling. EWcp might be one of these other brain regions involved in the control of energy expenditure via melanocortin pathways.

**Figure 11:**
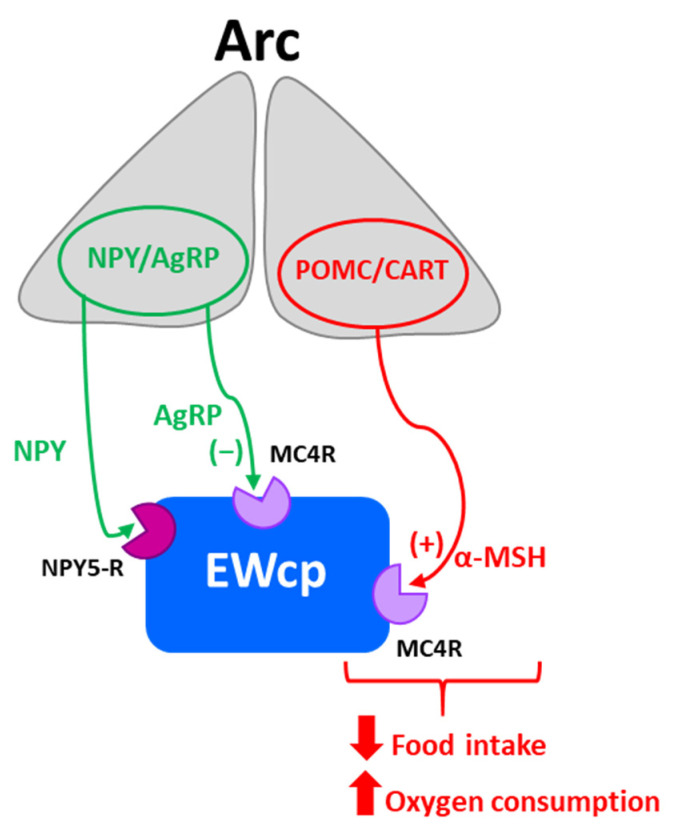
Connections of EWcp with melanocortin pathways from Arc. EWcp receives α-MSH (anorexigenic) and AgRP (orexigenic) projections from Arc and expresses MC4R. Fasting decreases α-MSH signal and increases AgRP signal in EWcp, whereas MC4R blockade in EWcp mimics the effects of fasting. α-MSH injection into EWcp decreased food intake and increased oxygen consumption. These data support the involvement of MC4R signaling in EWcp in the control of food intake and metabolism. For terms, see the list of abbreviations.

α-MSH and AgRP are mainly expressed in two distinct neuronal subpopulations in Arc. MC4R, which predominantly mediate the melanocortin satiety signaling and anorexigenic effect, are expressed in multiple CNS sites, including the preoptic area, PVN and LH-PeF [199,200], and also in EWcp [201]. Furedi et al. [201] reported the existence of α-MSH-ir and AgRP-ir fibers in close contact to Ucn-1 neurons in EWcp that express MC4R in rats (Figure 11). Fasting for 48 h decreased the α-MSH-ir signal and increased the AgRP-ir signal in EWcp, as well as augmented Fos B and Ucn-1 expression. Furthermore, injection of an MC4R blocker in EWcp mimicked the effects of fasting on Fos B and Ucn-1 expression, whereas α-MSH injection into EWcp decreased food intake in both free-fed rats and rats fed after fasting [201]. α-MSH injected into EWcp augmented oxygen consumption and initially evoked hyperthermia, which was rapidly counteracted by heat loss induced by tail vasodilation [201], and EWcp is part of the CNS circuit controlling the sympathetic activation of the tail artery [110]. These results depict an unexpected pairing of metabolic and thermoregulatory responses in the opposite direction from those observed in response to cold, fever and stress, in which hyperthermia and hypermetabolism are coordinated. Alternatively, α-MSH injection into EWcp could activate different subpopulations of neurons that do not become activated simultaneously in the animal and that are involved in different aspects of energy homeostasis (e.g., a subpopulation of EWcp neurons that decreases food intake via projections to VMM vs. a subpopulation of neurons that activates thermogenesis via projections to RPa). All these data support the involvement of MC4R signaling in EWcp in the control of food intake and metabolism, as well as in thermoregulation.

Melanocortins are also involved in WAT lipid mobilization. Central administration of a synthetic analog of α-MSH (melanotan II) decreased WAT mass by 50% compared to paired-fed rats, suggesting that this effect was caused by active lipolysis [202]. Central administration of melanotan II not only reduced food intake in both lean and obese rats (it was more potent in obese rats), but also elevated oxygen consumption and decreased the respiratory quotient in a dose-dependent manner [203]. Conversely, central administration of an MC4R antagonist increased both food intake and the body weight of lean rats, but had little effect in obese rats [203]. These observations demonstrate that central MC4R activation induces weight loss by inhibiting food intake, as well as by increasing energy expenditure and promoting WAT lipolysis. This effect on lipid mobilization is mediated by modulation of the sympathetic drive to WAT. PRV injection into WAT in hamsters demonstrated that numerous brain regions involved in the control of WAT activity express MC4R, comprising the classic areas involved in feeding behavior (Arc, PVN, LH-PeF, etc.) and thermogenesis (preoptic area, DMH, lateral parabrachial nucleus, caudal raphe, RVLM, etc.), plus additional regions including EWcp in which 78% of PRV-infected neurons expressed MC4R [103]. Considering the lipolytic effect of α-MSH and analogs injected into EWcp detailed above and that increased sympathetic outflow to WAT promotes lipid mobilization, the anatomical data linking MC4R expressing neurons in EWcp with the sympathetic circuit that controls WAT strongly support a role of EWcp in melanocortin-induced lipolysis in WAT. Besides the direct effect of MC4R activation in EWcp, α-MSH and AgRP could interact with other neuronal groups that express MC4R and project to EWcp, such as the preoptic area, PVN, Arc, LH-PeF and lateral parabrachial nucleus, known to contribute to the regulation of feeding and energy expenditure. Most likely, activation of MC4R neurons in different brain regions would be recruited depending on the specific environmental conditions and the animal’s nutritional state.

#### 6.3.3. Connections with the Paraventricular Hypothalamic Nucleus

Among PVN functions, some parvicellular subdivisions are involved in the control of food intake via connections with Arc and LH-PeF, and in the regulation of SNS activity by virtue of direct projections to SPNs or projections to brainstem presympathetic neurons. Retrograde labeling studies demonstrated that moderate numbers of neurons from all parvicellular subdivisions of PVN project to EWcp [78]. On the other hand, only sparse anterograde labeling of fibers from EWcp has been observed in PVN [79], consistent with sparse density of Ucn-1 fibers in PVN [77].

Despite the paucity of Ucn-1 innervation of the PVN, coupled to the low expression of CRF-R1 and almost no CRF-R2 [93], Ucn-1 injection into PVN has been reported to decrease food intake and metabolism. Ucn-1 injected into PVN also blocked the robust hyperphagia and increased RQ elicited by NPY injection into PVN [169]. Similarly, injection of NPY and/or ghrelin into the PVN stimulated food intake, promoted carbohydrate oxidation, and inhibited fat utilization; these effects were blocked by pretreatment with Ucn-1 injected into PVN [204]. Moreover, Ucn-1 injected into PVN increased plasma leptin and UCP-1 mRNA in BAT [166]. These observations suggest that Ucn-1 in PVN can modulate food intake and metabolic responses that are evoked by NPY. However, the lack of CRF receptor expression in PVN raises the possibility that Ucn-1 could be acting on CRF-R2 in PVN that might be located presynaptically, possibly on NPY terminals from Arc or in MCH and/or Orx terminals from LH-PeF.

Food intake needs to be terminated before the animal engages in overeating. The hyperphagia elicited by NPY in PVN is long-lasting [205], most likely due to the activation of metabotropic receptors. Nakamura and Nakamura [174] have proposed that there must be a mechanism to terminate NPY-mediated hunger signals in PVN after food consumption reaches a certain point, and these authors have proposed that Arc could provide negative feedback to avoid overeating. In this context, Ucn-1 from EWcp could also be part of this feedback loop to inhibit food intake. In a hypothetical scenario, NPY from Arc inhibits PVN and stimulates LH-PeF to increase food intake and, at some point, NPY might also activate Ucn-1 neurons in EWcp via NPY5R, which also receive inputs from Orx and MCH neurons from LH-PeF (that are activated by NPY) (Figure 9). All these diverse signals can be integrated in EWcp and build up until reaching a threshold, at which point Ucn-1 inputs could activate PVN and/or inhibit LH-PeF neurons to terminate the feeding response. Thus, EWcp might be an element of the feeding termination feedback pathway mediated by Arc, proposed by Nakamura and Nakamura [174], by virtue of integrating several hypothalamic inputs and, subsequently, conveying the output signal to hypothalamic and brainstem regions. Therefore, the potent anorectic effect of Ucn-1 from EWcp could be a component of a feedback pathway that detects adequate levels of food intake and ceases it to avoid excessive consumption.

#### 6.3.4. Connections with the Limbic System

Stress impacts food intake and metabolism. Some limbic regions, such as CeA and BNST, are part of the central circuits involved in stress responses and feeding behavior simultaneously, and EWcp is reciprocally linked to these regions. Retrograde tracer injections into CeA labeled numerous Ucn-1 neurons in caudal EWcp, whereas anterograde tracing from EWcp showed dense projections observed around CRF neurons in CeA and BNST [79]. Retrograde tracing from EWcp demonstrated labeled neurons in CeA and BNST [78]. Ucn-1 fibers were observed in CeA and BNST [77]. CeA expresses a low density of CRF-R1 mRNA, whereas BNST shows a high density of both CRF-R1 and CRF-R2 mRNA expression in rats and mice [93]. The bidirectional connections between limbic areas and EWcp might constitute a circuit that integrates both stress and feeding behavior signals to generate an output from EWcp to presympathetic neurons, which modulates the activity of effector organs via sympathetic innervation. Furthermore, these anatomical connections between the limbic system and EWcp suggest that EWcp might play a role in eating-related disorders linked to stress.

#### 6.3.5. Lack of Connections with the Ventromedial Hypothalamic Nucleus

Numerous studies have reported that manipulating the activity of the VMH impacts feeding behavior and BAT activity [206,207,208]. Moreover, the VMH expresses a high density of leptin receptors [150,209], as well as very high expression of CRF-R2 mRNA in both rats and mice [87,93]. Da Silva et al. [78] showed moderate numbers of neurons in the anterior and central subdivisions of VMH projecting to EWcp; however, Canteras et al. [210] reported a notable absence of anterograde labeled fibers in EWcp after PHA-L injection into dorsal or ventral VMH. Furthermore, VMH does not receive a substantial input from EWcp [79]. Thus, neuroanatomical data do not support a VMH–EWcp direct interaction. Although it is accepted that VMH modulates SNS activity [206,207,211], early anatomical studies failed to report direct VMH efferents within classic autonomic preganglionic centers in the brainstem [1,210]. Similarly, subsequent PRV tracing studies from BAT and WAT in rats showed that infection in VMH was notably absent [18,104,137,140,182], supporting the idea that VMH does not connect to the central circuits that control the sympathetic innervation of BAT and WAT, including EWcp neurons, in rats. Nevertheless, PRV injected in BAT or WAT in hamsters produced infection in VMH [103,181], suggesting that there could be species-specific differences in the central circuit(s) that controls adipose tissue activity. In rats, VMH is not part of the CNS circuit that controls the sympathetic innervation to other organs such as the spleen [74], kidney [75,212], adrenal gland [100], heart [109] and pancreas [105]. Thus, how the VMH connects to the circuit that controls BAT and WAT activity in rats remains a puzzling issue.

Despite the observation that VMH receives very sparse Ucn-1 innervation [77], some studies have examined the impact of injecting Ucn-1 into VMH. Ohata et al. [213] reported that Ucn-1 injection targeting VMH in rats inhibited food and water intake over 3 h, whereas the injection of antibodies against rat Ucn-1 into VMH potentiated food and water intake [213]; nevertheless, careful examination of that report shows that injections missed the VMH and were instead in the ventral aspect of DMH. That study also reported that injections of Ucn-1 into PVN or LH-PeF had no effect on feeding, but those injections were also outside their target; as described above (in 6c3), Ucn-1 injected into PVN had substantial effects on food intake and metabolism. However, it has been reported that mice with specific CRF-R2 knockdown in VMH using shRNA interference displayed increased food intake under basal conditions and after overnight fast [214]. After the discovery of urocortin-3 (Ucn-3) [215], which is a CRF-R2 selective endogenous ligand, the function of CRF-R2 in VMH has been re-examined. Ucn-3 is involved in several metabolic functions, such as feeding suppression, control of blood glucose levels, and thermoregulation (for a review, see [216]). Ucn-3 neurons are found predominantly in two areas: the median preoptic area and an unnamed region lateral to anterior PVN, extending rostrally into posterior BNST [217]; this second population is the major Ucn-3 afferent input to VMH, which shows very dense Ucn-3 nerve fibers and terminals [217]. Injection of Ucn-3 into VMH in rats suppressed feeding and elevated glucose and insulin plasma levels [218], and also decreased the quantity (feeding bout size) and duration (prolonged post-meal interval) of food intake over 3–4 h post-infusion [219]. A subset of CRF-R2 expressing glutamatergic neurons in VMH project to Arc, and the stimulation of CRF-R2 in VMH increased POMC mRNA expression in Arc, suggesting the existence of a direct excitatory pathway from VMH to Arc [218]. All these anatomical and functional data, together with the fact that VMH expresses high levels of CRF-R2 in rats and mice [93], suggest that the effects on food intake and glycemic control induced by activation of CRF-R2 in VMH are mediated by Ucn-3, which seems to be the endogenous ligand for CRF-R2 in this brain region. The VMH Ucn-3/CRF-R2 system seems to be part of a pathway that regulates certain aspects of energy homeostasis, but it is unclear at present how it may be connected with Ucn-1 neurons in EWcp or to presympathetic neurons involved in the control of BAT and WAT activity.

### 6.4. EWcp Expresses Receptors Involved in Hunger–Satiety Endocrine Signaling

EWcp neurons express several receptors to signaling molecules involved in energy homeostasis, including leptin and ghrelin. As expanded upon in the following sections, in each of these instances, evidence is available to suggest the involvement of EWcp in the actions of these peptides in energy homeostasis.

#### 6.4.1. Leptin Receptors in EWcp

The adipocyte-derived hormone leptin is essential for maintaining energy homeostasis because it regulates body weight, food intake and energy expenditure by signaling the brain (for a review, see [172]). Mutations of the leptin gene result in severe obesity as well as impaired thermogenesis and lipolysis [220,221,222], whereas leptin treatment reduces food intake and rapidly depletes adipose mass, mainly WAT [223]. The effects of leptin on energy expenditure are mediated by increased sympathetic outflow to BAT, which activates thermogenesis [224,225,226], and to WAT, which stimulates lipolysis [221]. Importantly, these effects are entirely mediated by the SNS because they can be abolished by the blockade or disruption of sympathetic inputs to these tissues (for a review, see [221,227]).

Leptin targets (neuronal populations expressing leptin receptors) such as the preoptic area and DMH [150] are essential components of the brain circuit that controls BAT thermogenesis. BAT presympathetic neurons in RPa were activated by i.v. leptin administration, which evoked an increase in BAT sympathetic nerve activity and BAT thermogenesis [226], most likely via direct projections from the preoptic area and DMH neurons that express leptin receptors. Similarly, hypothalamic neuronal groups that are part of the circuit controlling feeding, such as Arc, PVN and LH-PeF, also express leptin receptors [150]. Therefore, thermoregulation and food intake are both regulated by leptin signaling, apparently through distinct neuronal pathways that have been well documented (for review, see [172]). However, nutritional status, body temperature and energy expenditure must be closely interconnected to generate the appropriate output response under specific environmental conditions (e.g., ambient temperature, food availability, etc.). In this context, EWcp is strategically placed to play a role in mediating the effects of leptin signaling because EWcp expresses leptin receptors, both the short and long forms [209], in at least half of the Ucn-1/CART neurons in rats and mice [228,229]. Furthermore, PRV studies have demonstrated that EWcp neurons that express leptin receptors are involved in the central control of BAT activity in mice [102], and Ucn-1/CART neurons in EWcp are double-labeled when isogenic strains of PRV are injected into BAT and WAT simultaneously in rats [141].

Leptin and Ucn-1, apparently from EWcp, appear to interact to suppress feeding. Systemic leptin administration increased Ucn-1 content in EWcp [229], whereas fasting increased Ucn-1 mRNA and decreased leptin plasma levels [228]. Conversely, high fat diet, which increases leptin levels, decreased Ucn-1 mRNA expression in EWcp in rats [230]. Ucn-1 and leptin seem to exert a synergistic effect on appetite because co-administration of leptin and Ucn-1 at doses that are ineffective alone substantially suppressed feeding [231,232]. Leptin also increased CART mRNA expression in EWcp in rats [233], and the number of CART neurons in leptin-deficient (ob/ob) mice was reduced [233]. Leptin seems to modulate BAT and WAT activity via Ucn-1/CART neurons in EWcp. Injection of the leptin-conjugated neurotoxin saporin into the EWcp that killed leptin receptor-expressing neurons (loss of 50% of Ucn-1/CART neurons in EWcp) caused a significant increase in the weight of both BAT and WAT [25], supporting a role of leptin in EWcp in regulating the sympathetic outflow to both adipose tissues.

The effect of leptin on central Ucn-1 expression could be mediated via direct activation of leptin receptors in Ucn-1/CART neurons in EWcp. Leptin administration to mouse brain slices containing EWcp induced STAT3 phosphorylation in many Ucn-1 neurons 30 min after application, suggesting that leptin can directly induce the slow activation of these neurons [229]. Similarly, in vivo systemic administration of leptin also induced STAT3 phosphorylation in EWcp neurons expressing leptin receptors [229]. However, leptin administration to brain slices containing EWcp inhibited the electrical activity of 80% of patch-clamped Ucn-1 neurons [229]; these results show two different mechanisms of action of leptin on Ucn-1 neurons in EWcp, a rapid and reversible inhibition of their action potential firing and a slow activation of the STAT3 pathway, the latter suggesting a long-term effect of the response of Ucn-1 neurons to leptin activation. Nevertheless, leptin infusion into EWcp did not induce changes in the amount of Ucn-1 and CART [233]. Thus, although it is clear that leptin can influence EWcp, additional studies are needed to further clarify the effects and the role of leptin receptors in EWcp.

Despite the presence of leptin receptors in EWcp, the effects of circulating leptin on EWcp neurons and, subsequently, on adipose tissue activity could also be indirect via afferent projections to EWcp from other nuclei whose neurons express leptin receptors. After PRV injection in BAT in mice, infected neurons expressing leptin receptors were observed not only in EWcp, but also in areas that project directly to EWcp, such as PVN, Arc, LH-PeF, DMH and preoptic area [102]. All these brain regions are part of the BAT thermoregulatory circuit and are activated by cold or lipopolysaccharide-induced fever [234,235], as well as part of feeding pathways involved in the regulation of WAT activity [236]. For example, deletion of the leptin receptor gene in POMC/AgRP neurons in Arc decreased the sympathetic innervation to adipose tissue suggesting that leptin, by signaling specific central sites, not only modulates the sympathetic outflow to adipose tissues but also dynamically changes the architecture of the sympathetic innervation of these tissues [222]. There is also the possibility that some leptin receptors located in the EWcp region are presynaptic, and leptin modulates the release of other neurotransmitters in EWcp. Conversely, leptin receptors can be expressed presynaptically in axon terminals from EWcp neurons, and the activation of these receptors could modulate the release of Ucn-1/CART in projection targets such as PVN, LH-PeF or Arc.

An interesting observation pertaining to the effect of leptin on EWcp is that it seems to be dependent on energy status. Thus, in rats under basal conditions, leptin stimulates CART production in EWcp, but during acute restraint stress, which normally activates EWcp, leptin administration blunted the stress-evoked activation of EWcp and decreased CART mRNA expression [233]. In contrast to leptin, fasting does not affect stress-evoked EWcp activation. These results suggest that the activation of CART neurons from EWcp evoked by stress depends on the energy state of the animal [233]. It has been proposed that leptin-sensitive central pathways (i.e., containing leptin receptor-expressing neurons) may provide an integrative mechanism for the anorexic and thermoregulatory actions of leptin to maintain energy homeostasis under different conditions [227]. In this context, the fact that EWcp plays an important role in stress adaptation responses and in the control of energy expenditure, together with the expression of leptin receptors, makes it a good candidate to be a central component of an integrative leptin-sensitive circuit controlling energy homeostasis.

#### 6.4.2. Ghrelin Receptors in EWcp

Ghrelin is a hormone secreted by the gastrointestinal tract [237,238], which is involved in glucose metabolism and body weight regulation via increased food intake and stimulation of anabolic processes [239,240] (for a review, see [25]). Ghrelin plasma levels are an important indicator of energy deficiency [241] and are closely related to leptin levels. Central ghrelin administration blocked leptin-evoked feeding suppression [239], whereas fasting decreased plasma leptin but increased ghrelin [242], suggesting that ghrelin and leptin might play complementary and opposite roles in feeding regulation.

Ghrelin actions can be linked to EWcp. Ghrelin receptor (growth hormone secretagogue receptor; GHSR) mRNA, which is widely expressed in the brain [241], is found in the EWcp of rats and mice in moderate and very high densities, respectively [241]. In rats, GHSR is expressed in approximately 40% of CCK neurons in EWcp, as well as in some non-CCK neurons [241]. Furthermore, in a GHSR–GFP reporter mouse, 90% of GHSR-expressing neurons in EWcp co-express Ucn-1 [243]. I.c.v. administration of Ucn-1 in food-deprived rats decreased food intake, ghrelin plasma levels and pre-proghrelin mRNA levels in the gastrointestinal tract, whereas i.c.v. administration of a CRF-R2 antagonist restored plasma ghrelin and food intake [244]. These results suggest that Ucn-1 inhibits both ghrelin secretion and ghrelin-evoked food intake via CRF-R2 activation.

Centrally, ghrelin induces a positive energy balance not only by increasing food intake, but also through decreasing energy expenditure by suppressing BAT thermogenesis via inhibition of sympathetic outflow to BAT, which affects adiposity and body weight [245,246]. The PVN and Arc, which express GHSR, may be the sites at which ghrelin acts to inhibit BAT activity since microinjection of ghrelin in PVN or Arc suppressed noradrenaline release in BAT [247]. Nevertheless, EWcp also expresses GHSR, receives inputs from both PVN and Arc, is the main source of Ucn-1 known to inhibit ghrelin secretion, and is part of the CNS circuit that controls the sympathetic innervation of BAT. These observations suggest that ghrelin might inhibit Ucn-1 neurons in EWcp, directly or via afferents from PVN or Arc, to suppress BAT thermogenic activity and subsequently decrease energy expenditure.

With respect to WAT, i.c.v. administration of ghrelin increased adiposity by up-regulating lipogenic genes in WAT, which promote triglyceride synthesis and glucose uptake in adipocytes, and by down-regulating the expression of lipolytic enzymes genes [246,248]. These effects were independent from ghrelin-induced hyperphagia and were mediated by the SNS because i.c.v. ghrelin administration increased body weight gain in wild-type mice, but it had no effect on triple ß1-ß2-ß3-adrenoceptor knockout mice [246]. Again, this action of ghrelin on WAT may involve pathways through EWcp.

In addition to its role in metabolic functions, ghrelin is involved in stress responses, and it appears to reduce anxiety under chronic stress conditions [243,249]. Under basal conditions, ghrelin receptor knockout mice express more Fos in EWcp in general, and in Ucn-1 neurons in particular, than wild-type littermates. Acute stress activated ~80% of neurons expressing GHSR in EWcp (and these coexpressed Ucn-1) in wild-type mice, but failed to do so in ghrelin knockout mice, although these mice appeared more anxious after acute restraint stress [243]. These observations suggest that ghrelin interacts with Ucn-1 neurons in the EWcp to evoke the appropriate response to acute stress by reducing anxiety. It has been proposed that increases in ghrelin levels during stress exposure might be an endogenous stress-coping mechanism, and increased ghrelin levels may be required to prevent excessive anxiety [250]. The interaction between ghrelin and EWcp Ucn-1 neurons, both of which are implicated in the control of metabolism and stress responses, is consistent with EWcp being involved in the coordination of energy utilization with stress. Indeed, in this context, EWcp could play an important role in metabolic disorders associated with stress, such as bulimia or anorexia nervosa.

### 6.5. EWcp Contains Other Neuropeptides Involved in Energy Homeostasis

The role of central Ucn-1 in feeding suppression has been extensively documented. However, electrolytic lesions of EWcp significantly reduce, not increase, food intake in free-feeding mice and after food deprivation [36], suggesting that other neurotransmitters in EWcp besides Ucn-1 are involved in feeding behavior. EWcp neurons express neuropeptides known to be involved in stress responses and energy homeostasis, such as CART [4], CCK [10,51,83], SP [83], brain-derived neurotrophic factor (BDNF) [46,251] and nesfatin-1 [252,253]. As detailed below, some of these neuropeptides are co-expressed in the same neurons.

In contrast with Ucn-1, which is mainly located in EWcp, these neuropeptides are widely expressed in the CNS. This precludes the use of knockout mice or i.c.v. administration experiments to elucidate their role specifically from EWcp neurons and its projection targets. These neuropeptides colocalize in several EWcp subpopulations (for a review of the detailed distribution of subpopulations, see [5]), suggesting that they could be co-released. All CART neurons in EWcp colocalize with Ucn-1, although some Ucn-1 neurons lateral to EWcp do not contain CART [4]. Both neuropeptides are present in fiber terminals and are packed together into the same secretory vesicles [254]. Since both neuropeptides are involved in stress and feeding, it has been proposed that they could act synergistically in the regulation of responses to stress or environmental challenges, and modulate behaviors such as food intake [4]. Nevertheless, it has been reported that chronic stressors differentially affect Ucn-1 and CART neuronal activation in the rat EWcp [24]. Moreover, Ucn-1 axon terminals were observed in the periventricular part of the lateral septum, whereas CART axons terminated in the medial part of this nucleus, with only a partial overlap of both neuropeptides [4]. These observations raise the possibility that Ucn-1 and CART terminals arising from the same neurons do not colocalize in the same axon terminals and could contact distinct postsynaptic targets [4] and exert different effects. Nonetheless, each of these neuropeptides is expressed in EWcp neurons and each has been functionally linked to stress and/or energy homeostasis.

CART is a major neurotransmitter widely expressed in the CNS, involved in the regulation of several important functions such as food intake, body weight maintenance, energy homeostasis, stress responses, reward and addiction (for a review, see [55]). The role of EWcp CART neurons in energy homeostasis and their involvement in the control of sympathetic outflow and adipose tissue activity has been addressed in other portions of this review.

BDNF is another peptide that extensively co-localizes with Ucn-1 in EWcp in rats [255] and humans [251], and BDNF, CART and ER-β colocalize completely in EWcp in rats [46]. BDNF is involved in stress responses and stress-related disorders in a sex-dependent manner. BDNF neurons in EWcp were activated (Fos) by acute restraint stress in rats [256]. Maternal separation (a model of stress-induced depression) increased the content of Ucn-1 and BDNF in EWcp, but it blunted the Fos response to acute restraint stress; this was accompanied by reduced corticosterone responses in females but not in males [256], suggesting that EWcp might play a role in sex differences observed in stress-induced mood disorders. Interestingly, Ucn-1 and BDNF mRNA expression in EWcp was altered in suicide victims in a sex-dependent manner (males had 11 times more Ucn-1 mRNA and 4 times less BDNF mRNA in EWcp than females), suggesting that EWcp might contribute to the pathogenesis of major depression [251], which is more prevalent in females.

Nesfatin-1 also co-localizes extensively with Ucn-1 in EWcp. Nesfatin-1 is a protein fragment derived from the precursor nucleobindin-2 and it has been characterized as a satiety molecule [257]. In rats and mice, nesfatin-1 is expressed in several hypothalamic regions that regulate food intake (PVN, supraoptic nucleus, Arc, LH-PeF and zona incerta), and in brain nuclei connected to autonomic outflow and known to be activated by various stressors, such as EWcp, lateral parabrachial nucleus, locus coeruleus, ventrolateral medulla, medullary raphe nuclei and dorsal vagal complex, as well as in SPNs in the spinal cord [252,253,257,258,259]. Thus, nesfatin-1 distribution in the rodent CNS supports a role in centrally mediated actions involved in autonomic regulation [259]. Nesfatin-1 colocalizes with 90% of Ucn-1/CART neurons in EWcp in mice [9]. In rats, almost all EWcp neurons that colocalize Ucn-1, CART and nesfatin-1 also contain leptin receptors [228]. Nevertheless, Brailoiu et al. [252] have reported that nesfatin-1 also colocalizes with choline acetyltransferase in EW neurons, although not necessarily in motoneurons. In other brain regions, nesfatin-1 colocalizes with several neurotransmitters involved in feeding control such as MCH in LH-PeF (but not Orx) and POMC in Arc (but not NPY) [252,253]. CART colocalizes substantially in most brain regions that express nesfatin-1 (e.g., EWcp, locus coeruleus, raphe obscurus, RPa, NTS and SPNs in IML).

Nesfatin-1 displays a substantial anorectic activity. Several studies have shown that central administration of nesfatin-1 in rats and mice reduced dark phase food intake and body weight, and that this effect was faster and more sustained when injected into the fourth ventricle, suggesting the involvement of brainstem regions in this response [257] (for a review, see [260]). Conversely, central injection of the antisense oligonucleotide against the nucleobindin 2 gene increased food intake and body weight gain [257]. In addition to its role in food intake, nesfatin-1 is involved in stress responses. Acute restraint stress evoked Fos expression in EWcp neurons and doubled the Ucn-1, CART and nesfatin-1 mRNA content compared to controls [9]. Both acute and chronic stress increased Fos expression in EWcp neurons co-expressing CART and nesfatin-1 [24], whereas i.c.v. administration of nesfatin-1 elicited a strong anxiolytic response, suggesting a role in stress adaptation [261]. These observations demonstrate that nesfatin-1 is involved in the integrated control of feeding and energy expenditure, as well as in stress responses, possibly involving EWcp.

CCK, a gastrointestinal tract hormone that is also a neuropeptide in the brain (including EWcp) [262], is involved in the modulation of several central functions such as the control of food intake (anorectic action) [263], as well as anxiety and panic disorders [264]. SP, a neuropeptide of the tachykinin family that preferentially binds to the neurokinin 1 receptor, is colocalized in a subpopulation of EWcp neurons [10] distinct from the Ucn-1/CART subpopulation [5]. SP is present in limbic brain regions that are linked to stress, anxiety and depression (for a review, see [265,266]). More recently, SP has been implicated in alcohol addiction (for a review, see [5]).

Most studies on EWcp functions have focused on Ucn-1 as the main neurotransmitter because of its almost exclusive location in this nucleus and, to a lesser extent, on CART. Several reviews have addressed the role of the other neuropeptides expressed in EWcp in stress responses, food intake and addiction [5,8,25], suggesting that EWcp plays an integrative role. To the best of our knowledge, there are no studies addressing the effect of these neuropeptides specifically from EWcp on the modulation of sympathetic outflow and on BAT thermogenic and WAT lipolytic activities. Nevertheless, in the case of BDNF and nesfatin-1, their colocalization with Ucn-1 and/or CART and the existence of receptors for these neurotransmitters in some brain regions involved in energy homeostasis and/or autonomic control [267,268] suggest that projections from EWcp containing these neuropeptides might modulate the post-synaptic actions of Ucn-1 and/or CART if they were co-released on hypothalamic or presympathetic neurons. Further studies are needed to elucidate whether these neuropeptides arising from EWcp could also be individually released and exert specific actions independently of Ucn-1 and CART.

## 7. Proposed Model: EWcp as an Integrative Center of Multimodal Signals and Modulator of Sympathetic Output to Multiple Effector Organs to Maintain Energy Homeostasis under Different Conditions

**Figure 12:**
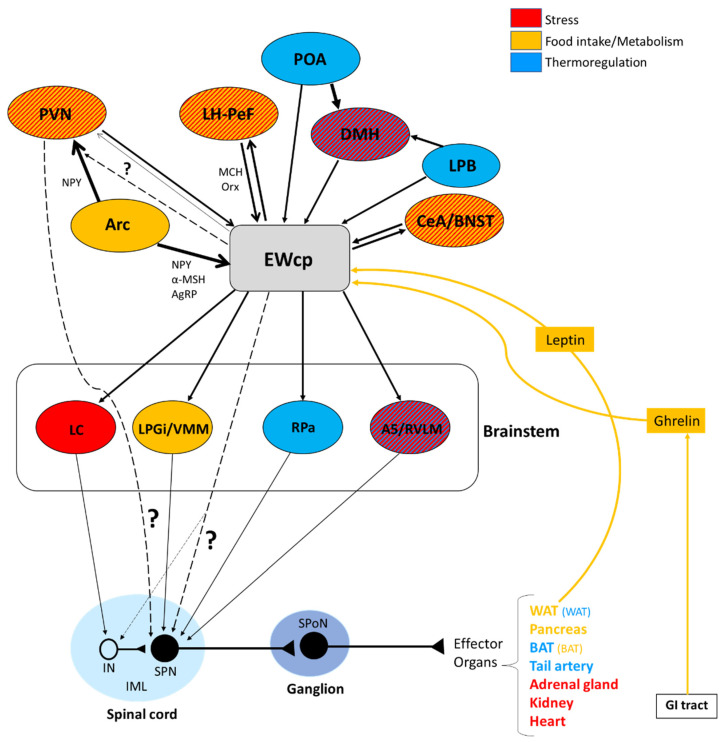
Proposed model for EWcp as an integrator of multimodal signals and modulator of sympathetic output to multiple effector organs to maintain energy homeostasis in rodents under different conditions. Brain regions and effector organs are color coded by the main function with which they are involved. Brain regions that are implicated in two functions are depicted by two colors. Similarly, effector organs involved in two functions are noted in large fonts for main functions and in smaller fonts for secondary roles. Arrow thickness represents projection density based on literature. Dashed lines (with question marks) represent hypothetical pathways based on neuroanatomical data, but without physiological information yet to support their role in the EWcp circuit. For example, the effect of Ucn-1 from EWcp in PVN could occur directly or via presynaptic activation of CRF-R2 on NPY terminals from Arc. In addition, it is unknown if this effect of Ucn-1 in PVN might affect the activity of presympathetic neurons known to project directly to SPNs in the spinal cord. The direct projection from EWcp to the spinal cord (to SPNs or interneurons) has been reported in classical tracing studies; nevertheless, transneuronal retrograde tracing with PRV does not support this direct projection (or if it exists, it is very sparse). For clarity purpose, the figure only includes relevant neuroanatomical connections from/to EWcp in the brain, excluding most connections among other brain regions. Abbreviations: GI, gastrointestinal; IN, interneurons; LPB, lateral parabrachial nucleus; LPGi, lateral paragigantocellular nucleus; POA, preoptic area; SPoN, sympathetic postganglionic neurons. For the rest of the terms, see the list of abbreviations.

Based on the neuroanatomical and physiological data described in this review, we propose a hypothetical model that embraces the EWcp as an essential hub for several pathways involved in energy homeostasis, mainly under stress conditions that require adjustment of energy demands (cold, flight-or-fight responses, fasting, etc.). In particular, we suggest that EWcp may be central in the integration of decreased feeding and increased energy utilization associated with stress. In this model, EWcp is a central integrator of multiple hunger and satiety signals from hypothalamic areas (Arc, PVN and LH-PeF), stress signals from limbic regions (CeA and BNST), signals from thermoregulatory pathways (preoptic area, DMH and lateral parabrachial nucleus), and hormonal signals (leptin, ghrelin). These diverse inputs convey information to EWcp about the animal’s metabolic state, as well as the energy demands required under specific conditions, particularly stress. After processing and integrating these multiple signals, the resultant output from EWcp (via brainstem and/or spinal cord) modulates the sympathetic outflow to BAT (to regulate thermogenesis) and WAT (to regulate lipolysis/lipogenesis) depending on the metabolic status and the environmental conditions. Moreover, EWcp is also part of the CNS circuitry that controls the sympathetic outflow to organs impacting cardiovascular adjustment, such as the heart, kidneys and certain blood vessels (tail artery), as well as to the adrenal gland, implicated in epinephrine release and β-adrenergic receptor-mediated mobilization of energy sources, and to the pancreas, responsible for insulin secretion that controls plasma glucose levels. All these mechanisms impact the metabolic state and, consequently, energy homeostasis. Figure 12 shows this hypothetical model in which EWcp integrates multimodal signals and modulates the sympathetic outflow to different effector organs and tissues.

## 8. Final Considerations

Besides its role in feeding/metabolic control and addiction, EWcp is involved in several important functions that are ultimately related to different aspects of stress responses, as well as stress coping and adaptation. Kozicz and colleagues [8,25] have proposed that CRF/CRF-R1 and Ucn-1/CRF-R2 constitute two separate but complementary systems that act coordinately during acute stress responses, but are differentially recruited during chronic stress. These authors propose that a possible role of EWcp is to coordinate stress responses with energy availability, since a successful stress response requires energy in accordance with the severity and nature of the stressor (and length of stress exposure). In addition to this role, it has been proposed that an important function of EWcp and the Ucn-1/CRF-R2 system is to counterbalance and terminate the action of the CRF/CRF-R1 system in order to restore energy and metabolic homeostasis after being perturbated by a stressor [38,158]. This compensation by the Ucn-1/CRF-R2 system seems to be achieved by modulating the sympathetic outflow to target organs [38].

The literature supports this hypothetical framework for this function of EWcp. Here, we have provided a neuroanatomical structure for the involvement of EWcp as a critical component of the CNS circuit controlling sympathetic outflow in general, as well as being polysynaptically linked to sympathetic-innervated effector organs that are essential for stress responses and energy homeostasis. In particular, we think that the role of EWcp in the control of BAT and WAT activities in a coordinated fashion might play an important role in ensuring appropriate energy utilization under different conditions, including stress responses. Moreover, EWcp is not only part of the central circuit that controls adipose tissue activity; it is also an important element of central feeding pathways, providing a substrate for the coordination of systems involved in energy expenditure and caloric intake. This coordination might confer the proper adjustment of energy demands in different nutritional states and environmental situations.

From a clinical perspective, the involvement of EWcp in stress adaptation responses and energy expenditure suggests that it might be a target for novel therapeutic approaches to treat metabolic disorders linked to stress, such as bulimia nervosa and anorexia. Moreover, EWcp could be a potential double-target for anti-obesity therapies, since sympathetic-mediated stimulation of WAT mobilization and long-lasting appetite suppression are the hallmarks of effective anti-obesity treatments, and EWcp is actively involved in both mechanisms.

The available literature concerning EWcp demonstrates that this nucleus is involved in various essential functions, some of them related to ensuring survival. The vast pattern of efferent and afferent projections from and to EWcp, respectively, and the likelihood that EWcp represents heterogenous neuronal populations based on their neuroanatomical connections, strongly supports its involvement in many more functions that have not yet been identified. Moreover, the phenotypically distinct subpopulations in EWcp expressing different neuropeptides further support this hypothesis that EWcp might be involved in a panoply of different functions. However, there are remarkable gaps in the field of EWcp research that need to be addressed. The lack of recordings from EWcp neurons in freely moving animals, as well as experiments involving optogenetic or chemogenetic manipulations, is quite remarkable, especially when compared to what has been achieved in other brain regions. This seems to be due, in part, to the widespread old conception held by most neuroscientists that EW is solely related to pupillary control, and the fact that the distinction between EWcp and EWpg is not widely acknowledged. Although manipulating EWcp is technically challenging because of its location, small size and rostro-caudal extension, it is feasible with contemporary techniques, particularly with optogenetic and chemogenetic tools targeting specific subpopulations of EWcp neurons based on phenotype. In order to move this field forward, future studies using these available methodologies are urgently needed to better understand the functional profile of EWcp, instead of relying on hypotheses based on indirect approaches.

Ultimately, these direct approaches will be needed to address the hypothesis that the sympathetic response modulated by the CNS, including EWcp, ensures adaptive responses necessary for the regulation of energy homeostasis. In this context, the role of EWcp in this coordinated sympathetic-driven mechanism deserves further attention.

## Data Availability

Not applicable.

## Abbreviations

α-MSH	alpha-melanocyte-stimulating hormone
ACTH	adrenocorticotropic hormone
AgRP	agouti-related peptide
Arc	arcuate nucleus
BAT	brown adipose tissue
BDA	biotinylated dextran amine
BDNF	brain-derived neurotrophic factor
BNST	bed nucleus of the stria terminalis
CART	cocaine- and amphetamine-regulated transcript
CCK	cholecystokinin
CeA	central amygdala
CNS	central nervous system
CRF	corticotropin-releasing factor
CRF-R1	corticotropin-releasing factor receptor 1
CRF-R2	corticotropin-releasing factor receptor 2
CTB	cholera toxin subunit B
DMH	dorsomedial hypothalamic nucleus
ER	estrogen receptor
EW	Edinger–Westphal nucleus
EWcp	centrally projecting Edinger–Westphal nucleus
EWpg	preganglionic Edinger–Westphal nucleus
GHSR	growth hormone secretagogue receptor
Gi	gigantocellular reticular nucleus
HPA	hypothalamic–pituitary–adrenal axis
HRP	horseradish peroxidase
IML	intermediolateral cell column
LH-PeF	lateral hypothalamus–perifornical area
MC4R	melanocortin-4 receptors
MCH	melanin-concentrating hormone
NPY	neuropeptide Y
NTS	nucleus of the solitary tract
Orx	orexin
PAG	periaqueductal gray
PHA-L	Phaseolus vulgaris lectin L
POMC	pro-opiomelanocortin
PRV	pseudorabies virus
PVN	paraventricular hypothalamic nucleus
RPa	raphe pallidus
RVLM	rostral ventrolateral medulla
SNS	sympathetic nervous system
SP	substance P
SPNs	sympathetic preganglionic neurons
Ucn-1	urocortin-1
Ucn-3	urocortin-3
UCP-1	uncoupling protein 1
VMH	ventromedial hypothalamic nucleus
VMM	ventromedial medulla
WAT	white adipose tissue

## References

[1] Saper C., Loewy A., Swanson L., Cowan W. (1976). Direct hypothalamo-autonomic connections. Brain Res..

[2] Loewy A., Saper C. (1978). Edinger-Westphal nucleus: Projections to the brain stem and spinal cord in the cat. Brain Res..

[3] Kozicz T., Bittencourt J., May P.J., Reiner A., Gamlin P., Palkovits M., Horn A., Toledo C.A., Ryabinin A.E. (2010). The Edinger-Westphal nucleus: A historical, structural, and functional perspective on a dichotomous terminology. J. Comp. Neurol..

[4] Kozicz T. (2003). Neurons colocalizing urocortin and cocaine and amphetamine-regulated transcript immunoreactivities are induced by acute lipopolysaccharide stress in the Edinger-Westphal nucleus in the rat. Neuroscience.

[5] Zuniga A., Ryabinin A.E. (2020). Involvement of Centrally Projecting Edinger–Westphal Nucleus Neuropeptides in Actions of Addictive Drugs. Brain Sci..

[6] Vaughan J., Donaldson C.J., Bittencourt J., Perrin M.H., Lewis K.A., Sutton S., Chan R., Turnbull A.V., Lovejoy D., Rivier C. (1995). Urocortin, a mammalian neuropeptide related to fish urotensin I and to corticotropin-releasing factor. Nat. Cell Biol..

[7] Yamamoto H., Maeda T., Fujimura M., Fujimiya M. (1998). Urocortin-like immunoreactivity in the substantia nigra, ventral tegmental area and Edinger-Westphal nucleus of rat. Neurosci. Lett..

[8] Kozicz T. (2007). On the role of urocortin 1 in the non-preganglionic Edinger–Westphal nucleus in stress adaptation. Gen. Comp. Endocrinol..

[9] Okere B., Xu L., Roubos E.W., Sonetti D., Kozicz T. (2010). Restraint stress alters the secretory activity of neurons co-expressing urocortin-1, cocaine- and amphetamine-regulated transcript peptide and nesfatin-1 in the mouse Edinger-Westphal nucleus. Brain Res..

[10] Innis R.B., Aghajanian G.K. (1986). Cholecystokinin-containing and nociceptive neurons in rat edinger-westphal nucleus. Brain Res..

[11] Zhang Z., Zhong P., Hu F., Barger Z., Ren Y., Ding X., Li S., Weber F., Chung S., Palmiter R.D. (2019). An Excitatory Circuit in the Perioculomotor Midbrain for Non-REM Sleep Control. Cell.

[12] Bachtell R.K., Tsivkovskaia N.O., Ryabinin A.E. (2002). Alcohol-Induced c-Fos Expression in the Edinger-Westphal Nucleus: Pharmacological and Signal Transduction Mechanisms. J. Pharmacol. Exp. Ther..

[13] Paxinos G., Watson C. (2009). The Rat Brain in Stereotaxic Coordinates: Compact Sixth Edition.

[14] Asaba K., Makino S., Hashimoto K. (1998). Effect of urocortin on ACTH secretion from rat anterior pituitary in vitro and in vivo: Comparison with corticotropin-releasing hormone. Brain Res..

[15] Weninger S.C., Peters L.L., Majzoub J.A. (2000). Urocortin Expression in the Edinger-Westphal Nucleus Is Up-Regulated by Stress and Corticotropin-Releasing Hormone Deficiency1. Endocrinology.

[16] Gaszner B., Csernus V., Kozicz T. (2004). Urocortinergic neurons respond in a differentiated manner to various acute stressors in the Edinger-Westphal nucleus in the rat. J. Comp. Neurol..

[17] Cespedes I.C., de Oliveira A.R., da Silva J.M., da Silva A.V., Sita L.V., Bittencourt J.C. (2010). mRNA expression of corticotropin-releasing factor and urocortin 1 after restraint and foot shock together with alprazolam administration. Peptides.

[18] Cano G., Passerin A.M., Schiltz J.C., Card J., Morrison S.F., Sved A.F. (2003). Anatomical substrates for the central control of sympathetic outflow to interscapular adipose tissue during cold exposure. J. Comp. Neurol..

[19] Cano G., Mochizuki T., Saper C.B. (2008). Neural Circuitry of Stress-Induced Insomnia in Rats. J. Neurosci..

[20] Korosi A., Schotanus S., Olivier B., Roubos E.W., Kozicz T. (2005). Chronic ether stress-induced response of urocortin 1 neurons in the Edinger–Westphal nucleus in the mouse. Brain Res..

[21] Kozicz T., Li M., Arimura A. (2001). The activation of urocortin immunoreactive neurons in the Einger-Westphal nucleus following stress in rats. Stress.

[22] Viau V., Sawchenko P.E. (2002). Hypophysiotropic neurons of the paraventricular nucleus respond in spatially, temporally, and phenotypically differentiated manners to acute vs. repeated restraint stress: Rapid publication. J. Comp. Neurol..

[23] Kozicz T., Korosi A., Korsman C., Tilburg-Ouwens D., Groenink L., Veening J., Van Der Gugten J., Roubos E., Olivier B. (2004). Urocortin expression in the Edinger-Westphal nucleus is down-regulated in transgenic mice over-expressing neuronal corticotropin-releasing factor. Neuroscience.

[24] Xu L., Bloem B., Gaszner B., Roubos E., Kozicz T. (2010). Stress-related changes in the activity of cocaine- and amphetamine-regulated transcript and nesfatin neurons in the midbrain non-preganglionic Edinger–Westphal nucleus in the rat. Neuroscience.

[25] Xu L., Scheenen W.J., Roubos E.W., Kozicz T. (2012). Peptidergic Edinger–Westphal neurons and the energy-dependent stress response. Gen. Comp. Endocrinol..

[26] Rouwette T., Klemann K., Gaszner B., Scheffer G., Roubos E., Scheenen W., Vissers K., Kozicz T. (2011). Differential responses of corticotropin-releasing factor and urocortin 1 to acute pain stress in the rat brain. Neuroscience.

[27] Kim Y., Perova Z., Mirrione M.M., Epradhan K., Henn F.A., Shea S., Eosten P., Eli B. (2016). Whole-Brain Mapping of Neuronal Activity in the Learned Helplessness Model of Depression. Front. Neural Circuits.

[28] Vetter D.E., Li C., Zhao L., Contarino A., Liberman M.C., Smith G.W., Marchuk Y., Koob G.F., Heinemann S.F., Vale W. (2002). Urocortin-deficient mice show hearing impairment and increased anxiety-like behavior. Nat. Genet..

[29] Wang X., Su H., Copenhagen L.D., Vaishnav S., Pieri F., Shope C.D., Brownell W.E., De Biasi M., Paylor R., Bradley A. (2002). Urocortin-Deficient Mice Display Normal Stress-Induced Anxiety Behavior and Autonomic Control but an Impaired Acoustic Startle Response. Mol. Cell. Biol..

[30] Zalutskaya A.A., Arai M., Bounoutas G.S., Abou-Samra A.B. (2007). Impaired adaptation to repeated restraint and decreased response to cold in urocortin 1 knockout mice. Am. J. Physiol. Metab..

[31] Spina M., Merlo-Pich E., Chan R.K., Basso A.M., Rivier J., Vale W., Koob G.F. (1996). Appetite-suppressing effects of urocortin, a CRF-related neuropeptide. Science.

[32] Moreau J.-L., Kilpatrick G., Jenck F. (1997). Urocortin, a novel neuropeptide with anxiogenic-like properties. NeuroReport.

[33] Slawecki C., Somes C., Rivier J., Ehlers C. (1999). Neurophysiological effects of intracerebroventricular administration of urocortin. Peptides.

[34] Spina M., Merlo-Pich E., Akwa Y., Balducci C., Basso A., Zorrilla E., Britton K., Rivier J., Vale W., Koob G. (2002). Time-dependent induction of anxiogenic-like effects after central infusion of urocortin or corticotropin-releasing factor in the rat. Psychopharmacology.

[35] Bachtell R.K., Weitemier A.Z., Ryabinin A.E. (2004). Lesions of the Edinger-Westphal nucleus in C57BL/6J mice disrupt ethanol-induced hypothermia and ethanol consumption. Eur. J. Neurosci..

[36] Weitemier A.Z., Ryabinin A.E. (2005). Lesions of the Edinger-Westphal nucleus alter food and water consumption. Behav. Neurosci..

[37] Bale T.L., Contarino A., Smith G.W., Chan R., Gold L.H., Sawchenko P.E., Koob G.F., Vale W.W., Lee K.-F. (2000). Mice deficient for corticotropin-releasing hormone receptor-2 display anxiety-like behaviour and are hypersensitive to stress. Nat. Genet..

[38] Bale T.L., Anderson K.R., Roberts A.J., Lee K.-F., Nagy T.R., Vale W.W. (2003). Corticotropin-Releasing Factor Receptor-2-Deficient Mice Display Abnormal Homeostatic Responses to Challenges of Increased Dietary Fat and Cold. Endocrinology.

[39] Coste S.C., Kesterson R.A., Heldwein K.A., Stevens S.L., Heard A.D., Hollis J.H., Murray S., Hill J.K., Pantely G.A., Hohimer A.R. (2000). Abnormal adaptations to stress and impaired cardiovascular function in mice lacking corticotropin-releasing hormone receptor-2. Nat. Genet..

[40] Turnbull A.V., Vaughan J., Rivier J.E., Vale W.W., Rivier C. (1999). Urocortin is not a significant regulator of intermittent electrofootshock-induced adrenocorticotropin secretion in the intact male rat. Endocrinology.

[41] Smagin G.N., Howell L.A., Ryan D.H., De Souza E.B., Harris R.B.S. (1998). The role of CRF2 receptors in corticotropin-releasing factor-and urocortin-induced anorexia. NeuroReport.

[42] Skutella T., Stöhr T., Probst J., Ramalho-Ortigao F., Holsboer F., Jirikowski G. (1994). Antisense Oligodeoxynucleotides for In Vivo Targeting of Corticotropin-Releasing Hormone mRNA: Comparison of Phosphorothioate and 3?-Inverted Probe Performance. Horm. Metab. Res..

[43] Schöbitz B., Pezeshki G., Probst J.C., Reul J.M., Skutella T., Stöhr T., Holsboer F., Spanagel R. (1997). Centrally administered oligodeoxynucleotides in rats: Occurrence of non-specific effects. Eur. J. Pharmacol..

[44] Derks N.M., Gaszner B., Roubos E.W., Kozicz L.T. (2010). Sex differences in urocortin 1 dynamics in the non-preganglionic Edinger–Westphal nucleus of the rat. Neurosci. Res..

[45] Derks N.M., Roubos E.W., Kozicz T. (2007). Presence of estrogen receptor beta in urocortin 1-neurons in the mouse non-preganglionic Edinger-Westphal nucleus. Gen. Comp. Endocrinol..

[46] Derks N.M., Gaszner B., Bernhardt K., Roubos E.W., Kozicz T. (2009). Sex-specific expression of BDNF and CART in the midbrain non-preganglionic Edinger–Westphal nucleus in the rat. Peptides.

[47] Haeger P., Andrés M.E., Forray M.I., Daza C., Araneda S., Gysling K. (2006). Estrogen receptors alpha and beta differentially regulate the transcriptional activity of the Urocortin gene. J. Neurosci..

[48] Taylor S.E., Klein L.C., Lewis B.P., Gruenewald T.L., Gurung R.A., Updegraff J.A. (2000). Biobehavioral responses to stress in females: Tend-and-befriend, not fight-or-flight. Psychol. Rev..

[49] Lantéri-Minet M., Isnardon P., De Pommery J., Menétrey D. (1993). Spinal and hindbrain structures involved in visceroception and visceronociception as revealed by the expression of Fos, Jun and Krox-24 proteins. Neuroscience.

[50] Bon K., Lantéri-Minet M., De Pommery J., Michiels J.F., Menétrey D. (1997). Cyclophosphamide cystitis as a model of visceral pain in rats: Minor effects at mesodiencephalic levels as revealed by the expression of c-fos, with a note on Krox-24. Exp. Brain Res..

[51] Maciewicz R., Phipps B., Grenier J., Poletti C. (1984). Edinger-Westphal nucleus: Cholecystokinin immunocytochemistry and projections to spinal cord and trigeminal nucleus in the cat. Brain Res..

[52] Rouwette T., Vanelderen P., De Reus M., Loohuis N.O., Giele J., Van Egmond J., Scheenen W., Scheffer G.J., Roubos E., Vissers K. (2012). Experimental neuropathy increases limbic forebrain CRF. Eur. J. Pain.

[53] Grill H.J., Markison S., Ginsberg A., Kaplan J.M. (2000). Long-term effects on feeding and body weight after stimulation of forebrain or hindbrain CRH receptors with urocortin. Brain Res..

[54] Cullen M.J., Ling N., Foster A.C., Pelleymounter M.A. (2001). Urocortin, corticotropin releasing factor-2 receptors and energy balance. Endocrinology.

[55] Lau J., Herzog H. (2014). CART in the regulation of appetite and energy homeostasis. Front. Neurosci..

[56] Chang S.L., Patel N.A., Romero A.A. (1995). Activation and desensitization of Fos immunoreactivity in the rat brain following ethanol administration. Brain Res..

[57] Turek V.F., Tsivkovskaia N.O., Hyytiä P., Harding S.E., Lê A.D., Ryabinin A.E. (2005). Urocortin 1 expression in five pairs of rat lines selectively bred for differences in alcohol drinking. Psychopharmacol..

[58] Weitemier A.Z., Woerner A., Bäckström P., Hyytiä P., Ryabinin A.E. (2001). Expression of c-Fos in Alko alcohol rats responding for ethanol in an operant paradigm. Alcohol. Clin. Exp. Res..

[59] Giardino W., Rodriguez E.D., Smith M.L., Ford M.M., Galili D., Mitchell S.H., Chen A., Ryabinin A.E. (2017). Control of chronic excessive alcohol drinking by genetic manipulation of the Edinger-Westphal nucleus urocortin-1 neuropeptide system. Transl. Psychiatry.

[60] Ozburn A.R., Mayfield R.D., Ponomarev I., Jones T.A., Blednov Y.A., Harris R.A. (2012). Chronic self-administration of alcohol results in elevated DeltaFosB: Comparison of hybrid mice with distinct drinking patterns. BMC Neurosci..

[61] Singh M.E., McGregor I.S., Mallet P.E. (2006). Perinatal exposure to delta(9)-tetrahydrocannabinol alters heroin-induced place conditioning and fos-immunoreactivity. Neuropsychopharmacology.

[62] Singh M., Verty A., Price I., McGregor I., Mallet P. (2004). Modulation of morphine-induced Fos-immunoreactivity by the cannabinoid receptor antagonist SR 141716. Neuropsychopharmacology.

[63] Spangler E., Cote D.M., Anacker A.M., Mark G.P., Ryabinin A.E. (2009). Differential sensitivity of the perioculomotor urocortin-containing neurons to ethanol, psychostimulants and stress in mice and rats. Neuroscience.

[64] Bachtell R., Tsivkovskaia N.O., Ryabinin A.E. (2003). Identification of temperature-sensitive neural circuits in mice using c-Fos expression mapping. Brain Res..

[65] Weitemier A.Z., Ryabinin A.E. (2005). Brain Region-Specific Regulation of Urocortin 1 Innervation and Corticotropin-Releasing Factor Receptor Type 2 Binding by Ethanol Exposure. Alcohol. Clin. Exp. Res..

[66] Giardino W., Cote D.M., Li J., Ryabinin A.E. (2012). Characterization of Genetic Differences within the Centrally Projecting Edinger-Westphal Nucleus of C57BL/6J and DBA/2J Mice by Expression Profiling. Front. Neuroanat..

[67] Ryabinin A.E., Weitemier A.Z. (2006). The urocortin 1 neurocircuit: Ethanol-sensitivity and potential involvement in alcohol consumption. Brain Res. Rev..

[68] Giardino W., Cocking D.L., Kaur S., Cunningham C.L., Ryabinin A.E. (2011). Urocortin-1 within the Centrally-Projecting Edinger-Westphal Nucleus Is Critical for Ethanol Preference. PLoS ONE.

[69] Selye H. (1936). A Syndrome produced by Diverse Nocuous Agents. Nat. Cell Biol..

[70] Selye H. (1956). The Stress of Life.

[71] Koob G.F., Le Moal M. (2001). Drug addiction, dysregulation of reward, and allostasis. Neuropsychopharmacology.

[72] Koob G.F., Moal M.L., Schulkin J. (2004). Drug Addiction and Allostasis. Allostasis, Homeostasis, and the Costs of Physiological Adaptation.

[73] Koob G.F., Le Moal M. (2008). Addiction and the Brain Antireward System. Annu. Rev. Psychol..

[74] Cano G., Sved A.F., Rinaman L., Rabin B.S., Card J.P. (2001). Characterization of the central nervous system innervation of the rat spleen using viral transneuronal tracing. J. Comp. Neurol..

[75] Cano G., Card J., Sved A.F. (2004). Dual viral transneuronal tracing of central autonomic circuits involved in the innervation of the two kidneys in rat. J. Comp. Neurol..

[76] Sved A.F., Cano G., Card J.P. (2001). Neuroanatomical specificity of the circuits controlling sympathetic outflow to different targets. Clin. Exp. Pharmacol. Physiol..

[77] Bittencourt J.C., Vaughan J., Arias C., Rissman R.A., Vale W.W., Sawchenko P.E. (1999). Urocortin expression in rat brain: Evidence against a pervasive relationship of urocortin-containing projections with targets bearing type 2 CRF receptors. J. Comp. Neurol..

[78] Da Silva A.V., Torres K.R., Haemmerle C.A., Céspedes I.C., Bittencourt J.C. (2013). The Edinger-Westphal nucleus II: Hypothalamic afferents in the rat. J. Chem. Neuroanat..

[79] Júnior E.D.D.S., Da Silva A.V., Da Silva K.R., Haemmerle C., Batagello D.S., Da Silva J.M., Lima L.B., Da Silva R.J., Diniz G., Sita L.V. (2015). The centrally projecting Edinger–Westphal nucleus—I: Efferents in the rat brain. J. Chem. Neuroanat..

[80] Saper C.B., Stornetta R.L. (2015). Central Autonomic System. The Rat Nervous System.

[81] Sugimoto T., Itoh K., Mizuno N. (1978). Direct projections from the Ediger-Westphal nucleus to the cerebellum and spinal cord in the cat: An HRP study. Neurosci. Lett..

[82] Luppi P.H., Sakai K., Fort P., Salvert D., Jouvet M. (1988). The nuclei of origin of monoaminergic, peptidergic, and cholinergic afferents to the cat nucleus reticularis magnocellularis: A double-labeling study with cholera toxin as a retrograde tracer. J. Comp. Neurol..

[83] Maciewicz R., Phipps B., Foote W., Aronin N., DiFiglia M. (1983). The distribution of substance P-containing neurons in the cat Edinger-Westphal nucleus: Relationship to efferent projection systems. Brain Res..

[84] Phipps B., Maciewicz R., Sandrew B., Poletti C., Foote W. (1983). Edinger-Westphal neurons that project to spinal cord contain substance P. Neurosci. Lett..

[85] Chung R., Mason P., Strassman A., Maciewicz R. (1987). Edinger-Westphal nucleus: Cells that project to spinal cord contain corticotropin-releasing factor. Neurosci. Lett..

[86] Klooster J., Beckers H., Vrensen G., Van Der Want J. (1993). The peripheral and central projections of the Edinger-Westphal nucleus in the rat. A light and electron microscopic tracing study. Brain Res..

[87] Kozicz T., Yanaihara H., Arimura A. (1998). Distribution of urocortin-like immunoreactivity in the central nervous system of the rat. J. Comp. Neurol..

[88] Morin S., Ling N., Liu X.-J., Kahl S., Gehlert D. (1999). Differential distribution of urocortin- and corticotropin-releasing factor-like immunoreactivities in the rat brain. Neuroscience.

[89] Weitemier A., Tsivkovskaia N., Ryabinin A. (2005). Urocortin 1 distribution in mouse brain is strain-dependent. Neuroscience.

[90] Korosi A., Kozicz T., Richter J., Veening J.G., Olivier B., Roubos E.W. (2007). Corticotropin-releasing factor, urocortin 1, and their receptors in the mouse spinal cord. J. Comp. Neurol..

[91] Hara Y., Ueta Y., Isse T., Kabashima N., Shibuya I., Hattori Y., Yamashita H. (1997). Increase of urocortin-like immunoreactivity in the rat hypothalamo-neurohypophysial system after salt loading and hypophysectomy. Neurosci. Lett..

[92] Chalmers D., Lovenberg T., De Souza E. (1995). Localization of novel corticotropin-releasing factor receptor (CRF2) mRNA expression to specific subcortical nuclei in rat brain: Comparison with CRF1 receptor mRNA expression. J. Neurosci..

[93] Van Pett K., Viau V., Bittencourt J.C., Chan R.K., Li H.-Y., Arias C., Prins G.S., Perrin M., Vale W., Sawchenko P.E. (2000). Distribution of mRNAs encoding CRF receptors in brain and pituitary of rat and mouse. J. Comp. Neurol..

[94] Tan L.A., Vaughan J.M., Perrin M.H., Rivier J.E., Sawchenko P.E. (2017). Distribution of corticotropin-releasing factor (CRF) receptor binding in the mouse brain using a new, high-affinity radioligand, [125I]-PD-Sauvagine. J. Comp. Neurol..

[95] Bittencourt J.C., Sawchenko P.E. (2000). Do Centrally Administered Neuropeptides Access Cognate Receptors? An Analysis in the Central Corticotropin-Releasing Factor System. J. Neurosci..

[96] Donaldson C.J., Sutton S.W., Perrin M.H., Corrigan A.Z., Lewis K.A., Rivier J.E., Vaughan J.M., Vale W.W. (1996). Cloning and characterization of human urocortin. Endocrinology.

[97] Potter E., Behan D.P., Linton E.A., Lowry P.J., Sawchenko P.E., Vale W.W. (1992). The central distribution of a corticotropin-releasing factor (CRF)-binding protein predicts multiple sites and modes of interaction with CRF. Proc. Natl. Acad. Sci. USA.

[98] Behan D.P., De Souza E.B., Lowry P.J., Potter E., Sawchenko P., Vale W.W. (1995). Corticotropin Releasing Factor (CRF) Binding Protein: A Novel Regulator of CRF and Related Peptides. Front. Neuroendocr..

[99] Card J., Enquist L.W. (2014). Transneuronal Circuit Analysis with Pseudorabies Viruses. Curr. Protoc. Neurosci..

[100] Kerman I.A., Akil H., Watson S.J. (2006). Rostral Elements of Sympatho-motor Circuitry: A Virally Mediated Transsynaptic Tracing Study. J. Neurosci..

[101] Shah N.S., Pugh P.C., Nam H., Rosenthal D.T., van Wijk D., Gaszner B., Kozicz T., Kerman I.A. (2013). A subset of presympathetic-premotor neurons within the centrally projecting Edinger–Westphal nucleus expresses urocortin-1. J. Chem. Neuroanat..

[102] Zhang Y., Kerman I.A., Laque A., Nguyen P., Faouzi M., Louis G.W., Jones J.C., Rhodes C., Münzberg H. (2011). Leptin-Receptor-Expressing Neurons in the Dorsomedial Hypothalamus and Median Preoptic Area Regulate Sympathetic Brown Adipose Tissue Circuits. J. Neurosci..

[103] Song C.K., Jackson R.M., Harris R.B.S., Richard D., Bartness T.J. (2005). Melanocortin-4 receptor mRNA is expressed in sympathetic nervous system outflow neurons to white adipose tissue. Am. J. Physiol. Integr. Comp. Physiol..

[104] Cano G., Hernan S.L., Allen H.R., Richie A.G., Ukasik D.R., Tupone D., Sved A.F. Central circuitry involved in the control of brown and white adipose tissue in normal rats and obese rats fed with high energy diet from early age. Proceedings of the 48th Annual Meeting of the Society for Neuroscience.

[105] Jansen A., Hoffman J., Loewy A. (1997). CNS sites involved in sympathetic and parasympathetic control of the pancreas: A viral tracing study. Brain Res..

[106] Jansen A., Wessendorf M., Loewy A. (1995). Transneuronal labeling of CNS neuropeptide and monoamine neurons after pseudorabies virus injections into the stellate ganglion. Brain Res..

[107] Farkas E., Jansen A.S., Loewy A.D. (1998). Periaqueductal gray matter input to cardiac-related sympathetic premotor neurons. Brain Res..

[108] Jansen A.S., Farkas E., Mac Sams J., Loewy A.D. (1998). Local connections between the columns of the periaqueductal gray matter: A case for intrinsic neuromodulation. Brain Res..

[109] Ter Horst G.J., Hautvast R.W., De Jongste M.J., Korf J. (1996). Neuroanatomy of cardiac activity-regulating circuitry: A transneuronal retrograde viral labelling study in the rat. Eur. J. Neurosci..

[110] Smith J.E., Jansen A.S., Gilbey M.P., Loewy A.D. (1998). CNS cell groups projecting to sympathetic outflow of tail artery: Neural circuits involved in heat loss in the rat. Brain Res..

[111] Trotter R.N., Stornetta R.L., Guyenet P.G., Roberts M.R. (2007). Transneuronal mapping of the CNS network controlling sympathetic outflow to the rat thymus. Auton. Neurosci..

[112] Dénes A., Boldogkoi Z., Uhereczky G., Hornyák A., Rusvai M., Palkovits M., Kovács K. (2005). Central autonomic control of the bone marrow: Multisynaptic tract tracing by recombinant pseudorabies virus. Neuroscience.

[113] Wee N.K., Lorenz M.R., Bekirov Y., Jacquin M.F., Scheller E.L. (2019). Shared Autonomic Pathways Connect Bone Marrow and Peripheral Adipose Tissues Across the Central Neuraxis. Front. Endocrinol..

[114] Strack A., Sawyer W., Hughes J., Platt K., Loewy A. (1989). A general pattern of CNS innervation of the sympathetic outflow demonstrated by transneuronal pseudorabies viral infections. Brain Res..

[115] Paxinos G., Watson C. (1997). The Rat Brain in Stereotaxic Coordinates: Compact Third Edition.

[116] Dampney R.A.L. (2015). Central mechanisms regulating coordinated cardiovascular and respiratory function during stress and arousal. Am. J. Physiol. Integr. Comp. Physiol..

[117] Kanbar R., DePuy S.D., West G.H., Stornetta R.L., Guyenet P.G. (2011). Regulation of visceral sympathetic tone by A5 noradrenergic neurons in rodents. J. Physiol..

[118] Wang L.A., Nguyen D.H., Mifflin S.W. (2018). CRHR2 (Corticotropin-Releasing Hormone Receptor 2) in the Nucleus of the Solitary Tract Contributes to Intermittent Hypoxia-Induced Hypertension. Hypertension.

[119] Milner T.A., Reis D.J., Pickel V.M., Aicher S.A., Giuliano R. (1993). Ultrastructural localization and afferent sources of corticotropin-releasing factor in the rat rostral ventrolateral medulla: Implications for central cardiovascular regulation. J. Comp. Neurol..

[120] Furlong T.M., McDowall L.M., Horiuchi J., Polson J.W., Dampney R.A.L. (2014). The effect of air puff stress on c-Fos expression in rat hypothalamus and brainstem: Central circuitry mediating sympathoexcitation and baroreflex resetting. Eur. J. Neurosci..

[121] Madden C.J., Stocker S., Sved A.F. (2006). Attenuation of homeostatic responses to hypotension and glucoprivation after destruction of catecholaminergic rostral ventrolateral medulla neurons. Am. J. Physiol. Integr. Comp. Physiol..

[122] Guyenet P.G., Stornetta R.L., Bochorishvili G., DePuy S.D., Burke P.G., Abbott S.B. (2013). C1 neurons: The body’s EMTs. Am. J. Physiol. Regul. Integr. Comp. Physiol..

[123] Zhao Z., Wang L., Gao W., Hu F., Zhang J., Ren Y., Lin R., Feng Q., Cheng M., Ju D. (2017). A Central Catecholaminergic Circuit Controls Blood Glucose Levels during Stress. Neuron.

[124] Brown M.R., Fisher L.A., Spiess J., Rivier C., Rivier J., Vale W. (1982). Corticotropin-Releasing Factor: Actions on the Sympathetic Nervous System and Metabolism. Endocrinology.

[125] Brown M.R., Fisher L.A., Spiess J., Rivier J., Rivier C., Vale W. (1982). Comparison of the biologic actions of corticotropin-releasing factor and sauvagine. Regul. Pept..

[126] Brown M.R., Fisher L.A., Webb V., Vale W.W., Rivier J.E. (1985). Corticotropin-releasing factor: A physiologic regulator of adrenal epinephrine secretion. Brain Res..

[127] Kiba T. (2004). Relationships between the autonomic nervous system and the pancreas including regulation of regeneration and apoptosis: Recent developments. Pancreas.

[128] Kataoka N., Hioki H., Kaneko T., Nakamura K. (2014). Psychological Stress Activates a Dorsomedial Hypothalamus-Medullary Raphe Circuit Driving Brown Adipose Tissue Thermogenesis and Hyperthermia. Cell Metab..

[129] Nakamura K. (2015). Neural circuit for psychological stress-induced hyperthermia. Temperature.

[130] Cannon B., Nedergaard J. (2004). Brown Adipose Tissue: Function and Physiological Significance. Physiol. Rev..

[131] Zhang W., Sunanaga J., Takahashi Y., Mori T., Sakurai T., Kanmura Y., Kuwaki T. (2010). Orexin neurons are indispensable for stress-induced thermogenesis in mice. J. Physiol..

[132] Giardino W.J., de Lecea L. (2014). Hypocretin (orexin) neuromodulation of stress and reward pathways. Curr. Opin. Neurobiol..

[133] Tyree S.M., Borniger J.C., De Lecea L. (2018). Hypocretin as a Hub for Arousal and Motivation. Front. Neurol..

[134] Yoshimichi G., Yoshimatsu H., Masaki T., Sakata T. (2001). Orexin-A regulates body temperature in coordination with arousal status. Exp. Biol. Med..

[135] Berthoud H.R., Patterson L.M., Sutton G.M., Morrison C., Zheng H. (2005). Orexin inputs to caudal raphe neurons involved in thermal, cardiovascular, and gastrointestinal regulation. Histochem. Cell Biol..

[136] Tupone D., Madden C.J., Cano G., Morrison S.F. (2011). An Orexinergic Projection from Perifornical Hypothalamus to Raphe Pallidus Increases Rat Brown Adipose Tissue Thermogenesis. J. Neurosci..

[137] Oldfield B., Giles M., Watson A., Anderson C., Colvill L., McKinley M. (2002). The neurochemical characterisation of hypothalamic pathways projecting polysynaptically to brown adipose tissue in the rat. Neuroscience.

[138] Krout K.E., Mettenleiter T.C., Loewy A.D. (2003). Single cns neurons link both central motor and cardiosympathetic systems: A double-virus tracing study. Neuroscience.

[139] Kerman I.A., Bernard R., Rosenthal D., Beals J., Akil H., Watson S.J. (2007). Distinct populations of presympathetic-premotor neurons express orexin or melanin-concentrating hormone in the rat lateral hypothalamus. J. Comp. Neurol..

[140] Oldfield B.J., Allen A.M., Davern P., Giles M.E., Owens N.C. (2007). Lateral hypothalamic ‘command neurons’ with axonal projections to regions involved in both feeding and thermogenesis. Eur. J. Neurosci..

[141] Cano G., Hernan S.L., Richie A.G., Allen H., Stanzani A., Tupone D., Sved A.F. Neuropeptidergic profile of hypothalamic neurons involved in the control of brown and white adipose tissue in normal rats and obese rats fed with high energy diet from early age. Proceedings of the 49th Annual Meeting of the Society for Neuroscience.

[142] Emmerzaal T., Doelen R.V., Roubos E., Kozicz T. (2013). Orexinergic innervation of urocortin1 and cocaine and amphetamine regulated transcript neurons in the midbrain centrally projecting Edinger–Westphal nucleus. J. Chem. Neuroanat..

[143] Bellinger D.L., Lorton D. (2014). Autonomic regulation of cellular immune function. Auton. Neurosci..

[144] Irwin M., Hauger R.L., Brown M., Britton K.T. (1988). CRF activates autonomic nervous system and reduces natural killer cytotoxicity. Am. J. Physiol..

[145] Venihaki M., Majzoub J.A. (1999). Animal Models of CRH Deficiency. Front. Neuroendocr..

[146] Jacobson L., Muglia L.J., Weninger S.C., Pacák K., Majzoub J.A. (2000). CRH deficiency impairs but does not block pituitary-adrenal responses to diverse stressors. Neuroendocrinology.

[147] Okamoto S., Ishikawa I., Kimura K., Saito M. (1998). Potent suppressive effects of urocortin on splenic lymphocyte activity in rats. NeuroReport.

[148] Kenney M.J., Ganta C.K. (2014). Autonomic Nervous System and Immune System Interactions. Compr. Physiol..

[149] Kenney M., Larsen B., McMurphy R., Mason D., Fels R. (2014). Dexmedetomidine and regulation of splenic sympathetic nerve discharge. Auton. Neurosci..

[150] Leshan R.L., Björnholm M., Münzberg H., Myers M.G. (2006). Leptin Receptor Signaling and Action in the Central Nervous System. Int. J. Obes..

[151] Morrison S.F. (2016). Central neural control of thermoregulation and brown adipose tissue. Auton. Neurosci..

[152] Roth J., Rummel C., Barth S.W., Gerstberger R., Hübschle T. (2009). Molecular Aspects of Fever and Hyperthermia. Immunol. Allergy Clin. N. Am..

[153] Stitt J.T. (1979). Fever versus hyperthermia. Fed. Proc..

[154] Figueiredo M., Fabricio A., Machado R., Melo M., Soares D., Souza G. (2010). Increase of core temperature induced by corticotropin-releasing factor and urocortin: A comparative study. Regul. Pept..

[155] De Fanti B.A., Martínez J. (2002). Central urocortin activation of sympathetic-regulated energy metabolism in Wistar rats. Brain Res..

[156] Telegdy G., Adamik A., Toth G. (2006). The action of urocortins on body temperature in rats. Peptides.

[157] Telegdy G., Adamik A. (2008). Involvement of CRH receptors in urocortin-induced hyperthermia. Peptides.

[158] Carlin K.M., Vale W.W., Bale T.L. (2006). Vital functions of corticotropin-releasing factor (CRF) pathways in maintenance and regulation of energy homeostasis. Proc. Natl. Acad. Sci. USA.

[159] Benoit S.C., Thiele T.E., Heinrichs S., Rushing P.A., Blake K.A., Steeley R.J. (2000). Comparison of central administration of corticotropin-releasing hormone and urocortin on food intake, conditioned taste aversion, and c-Fos expression☆. Peptides.

[160] Kishimoto T., Radulovic J., Radulovic M., Lin C.R., Schrick C., Hooshmand F., Hermanson O., Rosenfeld M.G., Spiess J. (2000). Deletion of Crhr2 reveals an anxiolytic role for corticotropin-releasing hormone receptor-2. Nat. Genet..

[161] Chaker B., Samra T.A., Datta N.S., Abou-Samra A.B. (2013). Altered Responses to Cold Environment in Urocortin 1 and Corticotropin-Releasing Factor Deficient Mice. Physiol. J..

[162] Boss O., Samec S., Paoloni-Giacobino A., Rossier C., Dulloo A., Seydoux J., Muzzin P., Giacobino J.-P. (1997). Uncoupling protein-3: A new member of the mitochondrial carrier family with tissue-specific expression. FEBS Lett..

[163] Fleury C., Neverova M., Collins S., Raimbault S., Champigny O., Levi-Meyrueis C., Bouillaud F., Seldin M.F., Surwit R.S., Ricquier D. (1997). Uncoupling protein-2: A novel gene linked to obesity and hyperinsulinemia. Nat. Genet..

[164] Champigny O., Ricquier D. (1990). Effects of Fasting and Refeeding on the Level of Uncoupling Protein mRNA in Rat Brown Adipose Tissue: Evidence for Diet-Induced and Cold-Induced Responses. J. Nutr..

[165] Boss O., Samec S., Kühne F., Bijlenga P., Assimacopoulos-Jeannet F., Seydoux J., Giacobino J.-P., Muzzin P. (1998). Uncoupling Protein-3 Expression in Rodent Skeletal Muscle Is Modulated by Food Intake but Not by Changes in Environmental Temperature. J. Biol. Chem..

[166] Kotz C.M., Wang C., Levine A.S., Billington C.J. (2002). Urocortin in the hypothalamic PVN increases leptin and affects uncoupling proteins-1 and -3 in rats. Am. J. Physiol. Integr. Comp. Physiol..

[167] Bray G. (2000). Reciprocal relation of food intake and sympathetic activity: Experimental observations and clinical implications. Int. J. Obes..

[168] Wang C., Mullet M.A., Glass M.J., Billington C.J., Levine A.S., Kotz C.M. (2001). Feeding inhibition by urocortin in the rat hypothalamic paraventricular nucleus. Am. J. Physiol. Integr. Comp. Physiol..

[169] Currie P.J., Coscina D.V., Bishop C., Coiro C.D., Koob G.F., Rivier J., Vale W. (2001). Hypothalamic paraventricular nucleus injections of urocortin alter food intake and respiratory quotient. Brain Res..

[170] Elmquist J.K., Coppari R., Balthasar N., Ichinose M., Lowell B.B. (2005). Identifying hypothalamic pathways controlling food intake, body weight, and glucose homeostasis. J. Comp. Neurol..

[171] Daniels D., Markison S., Grill H.J., Kaplan J.M. (2004). Central Structures Necessary and Sufficient for Ingestive and Glycemic Responses to Urocortin I Administration. J. Neurosci..

[172] Caron A., Lee S., Elmquist J.K., Gautron L. (2018). Leptin and brain-adipose crosstalks. Nat. Rev. Neurosci..

[173] Nedergaard J., Bengtsson T., Cannon B. (2011). New Powers of Brown Fat: Fighting the Metabolic Syndrome. Cell Metab..

[174] Nakamura K., Nakamura Y. (2018). Hunger and Satiety Signaling: Modeling Two Hypothalamomedullary Pathways for Energy Homeostasis. BioEssays.

[175] Lu D., Willard D., Patel I.R., Kadwell S., Overton L., Kost T., Luther M., Chen W., Woychik R., Wilkison W.O. (1994). Agouti protein is an antagonist of the melanocyte-stimulating-hormone receptor. Nat. Cell Biol..

[176] Ghamari-Langroudi M., Srisai D., Cone R.D. (2010). Multinodal regulation of the arcuate/paraventricular nucleus circuit by leptin. Proc. Natl. Acad. Sci. USA.

[177] Nakamura Y., Yanagawa Y., Morrison S.F., Nakamura K. (2017). Medullary Reticular Neurons Mediate Neuropeptide Y-Induced Metabolic Inhibition and Mastication. Cell Metab..

[178] Jansen A., Ter Horst G., Mettenleiter T., Loewy A. (1992). CNS cell groups projecting to the submandibular parasympathetic preganglionic neurons in the rat: A retrograde transneuronal viral cell body labeling study. Brain Res..

[179] Fay R.A., Norgren R. (1997). Identification of rat brainstem multisynaptic connections to the oral motor nuclei using pseudorabies virus: III. Lingual muscle motor systems. Brain Res. Rev..

[180] Giaconi E., Deriu F., Tolu E., Cuccurazzu B., Yates B.J., Billig I. (2005). Transneuronal tracing of vestibulo-trigeminal pathways innervating the masseter muscle in the rat. Exp. Brain Res..

[181] Nguyen N.L.T., Barr C.L., Ryu V., Cao Q., Xue B., Bartness T.J. (2017). Separate and shared sympathetic outflow to white and brown fat coordinately regulates thermoregulation and beige adipocyte recruitment. Am. J. Physiol. Integr. Comp. Physiol..

[182] Wiedmann N., Stefanidis A., Oldfield B.J. (2017). Characterization of the central neural projections to brown, white, and beige adipose tissue. FASEB J..

[183] Verberne A.J.M., Korim W.S., Sabetghadam A., Llewellyn-Smith I.J. (2016). Adrenaline: Insights into its metabolic roles in hypoglycaemia and diabetes. Br. J. Pharmacol..

[184] Verberne A.J.M., Sartor D.M. (2010). Rostroventrolateral medullary neurons modulate glucose homeostasis in the rat. Am. J. Physiol. Metab..

[185] Lee J., Raycraft L., Johnson A.W. (2021). The dynamic regulation of appetitive behavior through lateral hypothalamic orexin and melanin concentrating hormone expressing cells. Physiol. Behav..

[186] Barson J., Leibowitz S.F. (2017). Orexin/Hypocretin System: Role in Food and Drug Overconsumption. Int. Rev. Neurobiol..

[187] Able S.L., Ivarsson M., Fish R.L., Clarke T.L., McCourt C., Duckworth J.M., Napier C., Katugampola S.D. (2009). Localisation of melanin-concentrating hormone receptor 1 in rat brain and evidence that sleep parameters are not altered despite high central receptor occupancy. Eur. J. Pharmacol..

[188] Stanley S., Pinto S., Segal J., Pérez C.A., Viale A., DeFalco J., Cai X., Heisler L.K., Friedman J.M. (2010). Identification of neuronal subpopulations that project from hypothalamus to both liver and adipose tissue polysynaptically. Proc. Natl. Acad. Sci. USA.

[189] Adler E.S., Hollis J.H., Clarke I.J., Grattan D.R., Oldfield B.J. (2012). Neurochemical Characterization and Sexual Dimorphism of Projections from the Brain to Abdominal and Subcutaneous White Adipose Tissue in the Rat. J. Neurosci..

[190] Gaszner B., Korosi A., Palkovits M., Roubos E.W., Kozicz T. (2006). Neuropeptide Y activates urocortin 1 neurons in the nonpreganglionic Edinger-Westphal nucleus. J. Comp. Neurol..

[191] Sousa-Ferreira L., Garrido M., Nascimento-Ferreira I., Nobrega C., Santos-Carvalho A., Álvaro A.R., Rosmaninho-Salgado J., Kaster M., Kügler S., De Almeida L.P. (2011). Moderate Long-Term Modulation of Neuropeptide Y in Hypothalamic Arcuate Nucleus Induces Energy Balance Alterations in Adult Rats. PLoS ONE.

[192] Shi Y.-C., Lau J., Lin Z., Zhang H., Zhai L., Sperk G., Heilbronn R., Mietzsch M., Weger S., Huang X.-F. (2013). Arcuate NPY Controls Sympathetic Output and BAT Function via a Relay of Tyrosine Hydroxylase Neurons in the PVN. Cell Metab..

[193] Mountjoy K.G., Robbins L.S., Mortrud M.T., Cone R.D. (1992). The cloning of a family of genes that encode the melanocortin receptors. Science.

[194] Ericson M.D., Lensing C., Fleming K.A., Schlasner K.N., Doering S.R., Haskell-Luevano C. (2017). Bench-top to clinical therapies: A review of melanocortin ligands from 1954 to 2016. Biochim. Biophys. Acta Mol. Basis Dis..

[195] Ollmann M.M., Wilson B.D., Yang Y.-K., Kerns J.A., Chen Y., Gantz I., Barsh G.S. (1997). Antagonism of Central Melanocortin Receptors in Vitro and in Vivo by Agouti-Related Protein. Science.

[196] Huszar D., Lynch C.A., Fairchild-Huntress V., Dunmore J.H., Fang Q., Berkemeier L.R., Gu W., Kesterson R.A., Boston B.A., Cone R.D. (1997). Targeted Disruption of the Melanocortin-4 Receptor Results in Obesity in Mice. Cell.

[197] Butler A., Marks D.L., Fan W., Kuhn C.M., Bartolome M.V., Cone R.D. (2001). Melanocortin-4 receptor is required for acute homeostatic responses to increased dietary fat. Nat. Neurosci..

[198] Balthasar N., Dalgaard L., Lee C.E., Yu J., Funahashi H., Williams T., Ferreira M., Tang V., McGovern R.A., Kenny C.D. (2005). Divergence of Melanocortin Pathways in the Control of Food Intake and Energy Expenditure. Cell.

[199] Tatro J.B., Entwistle M.L. (1994). Heterogeneity of brain melanocortin receptors suggested by differential ligand binding in situ. Brain Res..

[200] Gelez H., Poirier S., Facchinetti P., Allers K.A., Wayman C., Bernabé J., Alexandre L., Giuliano F. (2010). Neuroanatomical distribution of the melanocortin-4 receptors in male and female rodent brain. J. Chem. Neuroanat..

[201] Füredi N., Nagy Á., Mikó A., Berta G., Kozicz T.L., Pétervári E., Balaskó M., Gaszner B. (2017). Melanocortin 4 receptor ligands modulate energy homeostasis through urocortin 1 neurons of the centrally projecting Edinger-Westphal nucleus. Neuropsychopharmacology.

[202] Raposinho P.D., White R.B., Aubert M.L. (2003). The melanocortin agonist Melanotan-II reduces the orexigenic and adipogenic effects of neuropeptide Y (NPY) but does not affect the NPY-driven suppressive effects on the gonadotropic and somatotropic axes in the male rat. J. Neuroendocr..

[203] Hwa J.J., Ghibaudi L., Gao J., Parker E.M. (2001). Central melanocortin system modulates energy intake and expenditure of obese and lean Zucker rats. Am. J. Physiol. Integr. Comp. Physiol..

[204] Currie P.J., Coiro C.D., Duenas R., Guss J.L., Mirza A., Tal N. (2011). Urocortin I inhibits the effects of ghrelin and neuropeptide Y on feeding and energy substrate utilization. Brain Res..

[205] Stanley B.G., Leibowitz S.F. (1985). Neuropeptide Y injected in the paraventricular hypothalamus: A powerful stimulant of feeding behavior. Proc. Natl. Acad. Sci. USA.

[206] Perkins M.N., Rothwell N.J., Stock M.J., Stone T. (1981). Activation of brown adipose tissue thermogenesis by the ventromedial hypothalamus. Nat. Cell Biol..

[207] Niijima A., Rohner-Jeanrenaud F., Jeanrenaud B. (1984). Role of ventromedial hypothalamus on sympathetic efferents of brown adipose tissue. Am. J. Physiol..

[208] Kageyama H., Osaka T., Kageyama A., Kawada T., Hirano T., Oka J., Miura M., Namba Y., Ricquier D., Shioda S. (2003). Fasting increases gene expressions of uncoupling proteins and peroxisome proliferator-activated receptor-gamma in brown adipose tissue of ventromedial hypothalamus-lesioned rats. Life Sci..

[209] Scott M.M., Lachey J.L., Sternson S.M., Lee C.E., Elias C.F., Friedman J.M., Elmquist J.K. (2009). Leptin targets in the mouse brain. J. Comp. Neurol..

[210] Canteras N.S., Simerly R.B., Swanson L.W. (1994). Organization of projections from the ventromedial nucleus of the hypothalamus: A Phaseolus vulgaris-leucoagglutinin study in the rat. J. Comp. Neurol..

[211] Marsh A.J., Fontes M.A., Killinger S., Pawlak D.B., Polson J.W., Dampney R.A. (2003). Cardiovascular Responses Evoked by Leptin Acting on Neurons in the Ventromedial and Dorsomedial Hypothalamus. Hypertension.

[212] Schramm L.P., Strack A.M., Platt K.B., Loewy A.D. (1993). Peripheral and central pathways regulating the kidney: A study using pseudorabies virus. Brain Res..

[213] Ohata H., Suzuki K., Oki Y., Shibasaki T. (2000). Urocortin in the ventromedial hypothalamic nucleus acts as an inhibitor of feeding behavior in rats. Brain Res..

[214] Chao H., DiGruccio M., Chen P., Li C. (2012). Type 2 Corticotropin-Releasing Factor Receptor in the Ventromedial Nucleus of Hypothalamus Is Critical in Regulating Feeding and Lipid Metabolism in White Adipose Tissue. Endocrinology.

[215] Lewis K., Li C., Perrin M.H., Blount A., Kunitake K., Donaldson C., Vaughan J., Reyes T.M., Gulyas J., Fischer W. (2001). Identification of urocortin III, an additional member of the corticotropin-releasing factor (CRF) family with high affinity for the CRF2 receptor. Proc. Natl. Acad. Sci. USA.

[216] Chen P., Van Hover C., Lindberg D., Li C. (2013). Central urocortin 3 and type 2 corticotropin-releasing factor receptor in the regulation of energy homeostasis: Critical involvement of the ventromedial hypothalamus. Front. Endocrinol..

[217] Li C., Vaughan J., Sawchenko P.E., Vale W.W. (2002). Urocortin III-Immunoreactive Projections in Rat Brain: Partial Overlap with Sites of Type 2 Corticotrophin-Releasing Factor Receptor Expression. J. Neurosci..

[218] Chen P., Vaughan J., Donaldson C., Vale W., Li C. (2010). Injection of Urocortin 3 into the ventromedial hypothalamus modulates feeding, blood glucose levels, and hypothalamic POMC gene expression but not the HPA axis. Am. J. Physiol. Metab..

[219] Fekete É.M., Inoue K., Zhao Y., Rivier J.E., Vale W.W., Szücs A., Koob G.F., Zorrilla E.P. (2006). Delayed Satiety-Like Actions and Altered Feeding Microstructure by a Selective Type 2 Corticotropin-Releasing Factor Agonist in Rats: Intra-Hypothalamic Urocortin 3 Administration Reduces Food Intake by Prolonging the Post-Meal Interval. Neuropsychopharmacology.

[220] Friedman J.M., Halaas J.L. (1998). Leptin and the regulation of body weight in mammals. Nat. Cell Biol..

[221] Zeng W., Pirzgalska R., Pereira M.M., Kubasova N., Barateiro A., Seixas E., Lu Y.-H., Kozlova A., Voss H., Martins G. (2015). Sympathetic Neuro-adipose Connections Mediate Leptin-Driven Lipolysis. Cell.

[222] Wang P., Loh K.H., Wu M., Morgan D.A., Schneeberger M., Yu X., Chi J., Kosse C., Kim D., Rahmouni K. (2020). A leptin–BDNF pathway regulating sympathetic innervation of adipose tissue. Nat. Cell Biol..

[223] Halaas J.L., Gajiwala K.S., Maffei M., Cohen S.L., Chait B.T., Rabinowitz D., Lallone R.L., Burley S., Friedman J.M. (1995). Weight-reducing effects of the plasma protein encoded by the obese gene. Science.

[224] Haynes W.G., Morgan D.A., Walsh S.A., Mark A.L., Sivitz W.I. (1997). Receptor-mediated regional sympathetic nerve activation by leptin. J. Clin. Investig..

[225] Scarpace P.J., Matheny M. (1998). Leptin induction of UCP1 gene expression is dependent on sympathetic innervation. Am. J. Physiol. Metab..

[226] Morrison S.F. (2004). Activation of 5-HT1A receptors in raphe pallidus inhibits leptin-evoked increases in brown adipose tissue thermogenesis. Am. J. Physiol. Integr. Comp. Physiol..

[227] Rezai-Zadeh K., Münzberg H. (2013). Integration of sensory information via central thermoregulatory leptin targets. Physiol. Behav..

[228] Xu L., Bloem B., Gaszner B., Roubos E.W., Kozicz T. (2009). Sex-specific effects of fasting on urocortin 1, cocaine- and amphetamine-regulated transcript peptide and nesfatin-1 expression in the rat Edinger-Westphal nucleus. Neuroscience.

[229] Xu L., Scheenen W.J.J.M., Leshan R.L., Patterson C.M., Elias C.F., Bouwhuis S., Roubos E.W., Myers M.G., Kozicz T. (2011). Leptin Signaling Modulates the Activity of Urocortin 1 Neurons in the Mouse Nonpreganglionic Edinger-Westphal Nucleus. Endocrinology.

[230] Legendre A., Papakonstantinou E., Roy M.-C., Richard D., Harris R.B.S. (2007). Differences in response to corticotropin-releasing factor after short- and long-term consumption of a high-fat diet. Am. J. Physiol. Integr. Comp. Physiol..

[231] Pan W., Tu H., Hsuchou H., Daniel J., Kastin A.J. (2007). Unexpected Amplification of Leptin-Induced Stat3 Signaling by Urocortin: Implications for Obesity. J. Mol. Neurosci..

[232] Pan W., Kastin A.J. (2008). Urocortin and the brain. Prog. Neurobiol..

[233] Xu L., Janssen D., Van Der Knaap N., Roubos E.W., Leshan R.L., Myers M.G., Gaszner B., Kozicz T. (2014). Integration of stress and leptin signaling by CART producing neurons in the rodent midbrain centrally projecting Edinger-Westphal nucleus. Front. Neuroanat..

[234] DiMicco J.A., Zaretsky D. (2007). The dorsomedial hypothalamus: A new player in thermoregulation. Am. J. Physiol. Integr. Comp. Physiol..

[235] Morrison S.F., Nakamura K., Madden C.J. (2008). Central control of thermogenesis in mammals. Exp. Physiol..

[236] Blaszkiewicz M., Townsend K.L. (2016). Adipose Tissue and Energy Expenditure: Central and Peripheral Neural Activation Pathways. Curr. Obes. Rep..

[237] Date Y., Kojima M., Hosoda H., Sawaguchi A., Mondal M.S., Suganuma T., Matsukura S., Kangawa K., Nakazato M. (2000). Ghrelin, a novel growth hormone-releasing acylated peptide, is synthesized in a distinct endocrine cell type in the gastrointestinal tracts of rats and humans. Endocrinology.

[238] Kojima M., Hosoda H., Date Y., Nakazato M., Matsuo H., Kangawa K. (1999). Ghrelin is a growth-hormone-releasing acylated peptide from stomach. Nat. Cell Biol..

[239] Nakazato M., Murakami N., Date Y., Kojima M., Matsuo H., Kangawa K., Matsukura S. (2001). A role for ghrelin in the central regulation of feeding. Nat. Cell Biol..

[240] Wren A.M., Small C.J., Abbott C.R., Dhillo W., Seal L.J., Cohen M.A., Batterham R., Taheri S., Stanley S.A., Ghatei M.A. (2001). Ghrelin Causes Hyperphagia and Obesity in Rats. Diabetes.

[241] Zigman J.M., Jones J.E., Lee C.E., Saper C.B., Elmquist J.K. (2005). Expression of ghrelin receptor mRNA in the rat and the mouse brain. J. Comp. Neurol..

[242] Luque R.M., Huang Z.H., Shah B., Mazzone T., Kineman R.D. (2007). Effects of leptin replacement on hypothalamic-pituitary growth hormone axis function and circulating ghrelin levels in ob/ob mice. Am. J. Physiol. Metab..

[243] Spencer S., Xu L., Clarke M.A., Lemus M., Reichenbach A., Geenen B., Kozicz T., Andrews Z.B. (2012). Ghrelin Regulates the Hypothalamic-Pituitary-Adrenal Axis and Restricts Anxiety After Acute Stress. Biol. Psychiatry.

[244] Yakabi K., Noguchi M., Ohno S., Ro S., Onouchi T., Ochiai M., Takabayashi H., Takayama K., Harada Y., Sadakane C. (2011). Urocortin 1 reduces food intake and ghrelin secretion via CRF2 receptors. Am. J. Physiol. Metab..

[245] Yasuda T., Masaki T., Kakuma T., Yoshimatsu H. (2003). Centrally administered ghrelin suppresses sympathetic nerve activity in brown adipose tissue of rats. Neurosci. Lett..

[246] Theander-Carrillo C., Wiedmer P., Cettour-Rose P., Nogueiras R., Perez-Tilve D., Pfluger P., Castaneda T.R., Muzzin P., Schurmann A., Szanto I. (2006). Ghrelin action in the brain controls adipocyte metabolism. J. Clin. Investig..

[247] Mano-Otagiri A., Ohata H., Iwasaki-Sekino A., Nemoto T., Shibasaki T. (2009). Ghrelin suppresses noradrenaline release in the brown adipose tissue of rats. J. Endocrinol..

[248] Perez-Tilve D., Heppner K., Kirchner H., Lockie S.H., Woods S.C., Smiley D.L., Tschöp M., Pfluger P. (2011). Ghrelin-induced adiposity is independent of orexigenic effects. FASEB J..

[249] Lutter M., Sakata I., Osborne-Lawrence S., Rovinsky S.A., Anderson J.G., Jung S., Birnbaum S., Yanagisawa M., Elmquist J.K., Nestler E.J. (2008). The orexigenic hormone ghrelin defends against depressive symptoms of chronic stress. Nat. Neurosci..

[250] Bali A., Jaggi A. (2016). An Integrative Review on Role and Mechanisms of Ghrelin in Stress, Anxiety and Depression. Curr. Drug Targets.

[251] Kozicz T., Tilburg-Ouwens D., Faludi G., Palkovits M., Roubos E. (2008). Gender-related urocortin 1 and brain-derived neurotrophic factor expression in the adult human midbrain of suicide victims with major depression. Neuroscience.

[252] Brailoiu G.C., Dun S.L., Brailoiu E., Inan S., Yang J., Chang J.K., Dun N.J. (2007). Nesfatin-1: Distribution and Interaction with a G Protein-Coupled Receptor in the Rat Brain. Endocrinology.

[253] Foo K.S., Brismar H., Broberger C. (2008). Distribution and neuropeptide coexistence of nucleobindin-2 mRNA/nesfatin-like immunoreactivity in the rat CNS. Neuroscience.

[254] Van Wijk D.C., Xu L., Spiegelberg L., Struik R.F., Meijer K.H., Gaszner B., Kozicz T., Roubos E.W. (2009). Ultrastructural and immunocytochemical characterization of the rat non-preganglionic Edinger–Westphal nucleus. Gen. Comp. Endocrinol..

[255] Gaszner B., Van Wijk D.C.W.A., Korosi A., Józsa R., Roubos E.W., Kozicz T. (2009). Diurnal expression of period 2 and urocortin 1 in neurones of the non-preganglionic Edinger-Westphal nucleus in the rat. Stress.

[256] Gaszner B., Jensen K.-O., Farkas J., Reglődi D., Csernus V., Roubos E.W., Kozicz T. (2009). Effects of maternal separation on dynamics of urocortin 1 and brain-derived neurotrophic factor in the rat non-preganglionic Edinger-Westphal nucleus. Int. J. Dev. Neurosci..

[257] Oh S., Shimizu H., Satoh T., Okada S., Adachi S., Inoue K., Eguchi H., Yamamoto M., Imaki T., Hashimoto K. (2006). Identification of nesfatin-1 as a satiety molecule in the hypothalamus. Nature.

[258] Goebel-Stengel M., Wang L., Stengel A., Taché Y. (2011). Localization of nesfatin-1 neurons in the mouse brain and functional implication. Brain Res..

[259] Goebel-Stengel M., Wang L. (2013). Central and peripheral expression and distribution of NUCB2/nesfatin-1. Curr. Pharm. Des..

[260] Stengel A., Goebel M., Taché Y. (2011). Nesfatin-1: A novel inhibitory regulator of food intake and body weight. Obes. Rev..

[261] Merali Z., Cayer C., Kent P., Anisman H. (2008). Nesfatin-1 increases anxiety- and fear-related behaviors in the rat. Psychopharmacology.

[262] Dockray G.J. (1980). Cholecystokinins in rat cerebral cortex: Identification, purification and characterization by immunochemical methods. Brain Res..

[263] Broberger C., Hökfelt T. (2001). Hypothalamic and vagal neuropeptide circuitries regulating food intake. Physiol. Behav..

[264] Bradwejn J., Koszycki D., Meterissian G. (1990). Cholecystokinin-Tetrapeptide Induces Panic Attacks in Patients with Panic Disorder. Can. J. Psychiatry.

[265] Vanderah T., Sandweiss A. (2015). The pharmacology of neurokinin receptors in addiction: Prospects for therapy. Subst. Abus. Rehabilitation.

[266] Schank J.R., Heilig M. (2017). Substance P and the Neurokinin-1 Receptor: The New CRF. Int. Rev. Neurobiol..

[267] Prinz P., Goebel-Stengel M., Teuffel P., Rose M., Klapp B.F., Stengel A. (2016). Peripheral and central localization of the nesfatin-1 receptor using autoradiography in rats. Biochem. Biophys. Res. Commun..

[268] Yan Q., Radeke M.J., Matheson C.R., Talvenheimo J., Welcher A.A., Feinstein S.C. (1997). Immunocytochemical localization of TrkB in the central nervous system of the adult rat. J. Comp. Neurol..

